# V1R promoters are well conserved and exhibit common putative regulatory motifs

**DOI:** 10.1186/1471-2164-8-253

**Published:** 2007-07-25

**Authors:** Robert Stewart, Robert P Lane

**Affiliations:** 1 Department of Molecular Biology and Biochemistry, Wesleyan University, Middletown, Connecticut, 06459 USA

## Abstract

**Background:**

The mouse vomeronasal organ (VNO) processes chemosensory information, including pheromone signals that influence reproductive behaviors. The sensory neurons of the VNO express two types of chemosensory receptors, V1R and V2R. There are ~165 V1R genes in the mouse genome that have been classified into ~12 divergent subfamilies. Each sensory neuron of the apical compartment of the VNO transcribes only one of the repertoire of V1R genes. A model for mutually exclusive V1R transcription in these cells has been proposed in which each V1R gene might compete stochastically for a single transcriptional complex. This model predicts that the large repertoire of divergent V1R genes in the mouse genome contains common regulatory elements. In this study, we have characterized V1R promoter regions by comparative genomics and by mapping transcription start sites.

**Results:**

We find that transcription is initiated from ~1 kb promoter regions that are well conserved within V1R subfamilies. While cross-subfamily homology is not evident by traditional methods, we developed a heuristic motif-searching tool, *LogoAlign*, and applied this tool to identify motifs shared within the promoters of all V1R genes. Our motif-searching tool exhibits rapid convergence to a relatively small number of non-redundant solutions (97% convergence). We also find that the best motifs contain significantly more information than those identified in controls, and that these motifs are more likely to be found in the immediate vicinity of transcription start sites than elsewhere in gene blocks. The best motifs occur near transcription start sites of ~90% of all V1R genes and across all of the divergent subfamilies. Therefore, these motifs are candidate binding sites for transcription factors involved in V1R co-regulation.

**Conclusion:**

Our analyses show that V1R subfamilies have broad and well conserved promoter regions from which transcription is initiated. Results from a new motif-finding algorithm, *LogoAlign*, designed for this context and more generally for searching large, hierarchical datasets, suggest the existence of common information-rich regulatory motifs that are shared across otherwise divergent V1R subfamilies.

## Background

Most mammals detect odorant chemicals using two sensory systems. The main olfactory epithelium of the nose consists of olfactory sensory neurons that express odorant receptor (OR) proteins [1]. OR proteins bind inspired odorants and generate action potentials that signal odorant information to the olfactory bulb [2]. Most mammals also possess a second chemosensory organ, the vomeronasal organ (VNO), whose chemosensory functions have been closely associated with reproductive processes [3]. The sensory neurons of the VNO express two types of receptors, V1R and V2R (VRs) [4-8]. These sensory neurons project to the "accessory olfactory bulb" located posterior to the main olfactory bulb [3].

The OR, V1R, and V2R proteins are members of the G-coupled-receptor (GPCR) superfamily. The mouse genome encodes ~1600 OR genes [9,10], ~165 V1R genes [11-13], and ~60 V2R genes [14]. The OR and V1R genes have a compact gene structure comprising a single coding exon (~1 kb) and 1–2 short exons in their untranslated regions (UTRs). Putative transcription start sites have been typically mapped to positions < 10 kb upstream of coding exons [15]. These compact genes are predominantly organized in clusters at > 40 chromosomal loci in mouse [9,13]. These clusters are rarely, if ever, interrupted by non-OR/non-V1R genes.

Each sensory neuron in the mouse olfactory epithelium and VNO expresses only one allele of one gene from the large repertoire of ORs and V1Rs in the genome [16,17]. Mutually exclusive receptor expression permits the functional specialization of individual sensory neurons in both systems; i.e., individual sensory neurons in both the main nose/VNO are specialized for the odorant-binding functions of the receptor allele expressed in that cell. Despite the fact that OR and V1R proteins do not share sequence homology, transduce signals via different G proteins and are expressed in non-overlapping cell types [6], this shared feature of mutually exclusive expression has fueled speculation that these systems may utilize a similar regulatory strategy.

Mutually exclusive expression is accomplished in other systems by several diverse mechanisms. Typically, differential gene expression is governed by *cis *enhancers that bind transcription factor combinations, which in a deterministic way, dictate whether a gene is expressed or not at a given developmental time or place. Applying this model to mutually exclusive OR transcription, such a mechanism predicts that each OR promoter has a unique *cis *regulatory code, and that each sensory neuron would express a specific combination of transcription factors sufficient to activate only one OR promoter. The fact that duplicate OR transgenes, with identical *cis *regulatory sequences, are not co-expressed in the same cells indicates that mutual exclusive OR transcription is not regulated by a strict deterministic model [18]. A second category of mechanisms includes non-deterministic or stochastic processes; e.g., competition for a single-copy regulatory complex in the nucleus. One biological precedent for a stochastic competitive mechanism is the mutually exclusive expression of trypanosome *variable surface glycoprotein *genes by a single-copy "*pol *body" regulatory complex [19]. This model proposes that a unique assembly of transcription factors in the nucleus is able to stably associate with only one gene at a time. The recent observation that active OR genes (but not inactive OR genes) interact with a unique locus in the nucleus [20], termed the "H region", that was previously characterized as having strong enhancement activity [18], is consistent with this model. Additional study of the "H region" will be required in order to elucidate its precise role in the establishment of mutually exclusive OR transcription.

Stochastic competition models predict that the competing genes share common *cis *regulatory sequences, which interact with common transcription factors that are part of the regulatory complex. A recent study of the regulatory region of one mouse OR gene revealed that a small transgene containing merely ~400 bp upstream genome sequence is sufficient to drive mutual exclusive expression of this transgene [21]. This, and other similar mini-transgene studies suggest that in general, putative "universal" *cis *regulatory elements are likely to reside close to transcription start sites. While no V1R mini-transgene experiments have yet been reported, previous comparative genomic analysis of one V1R gene cluster suggests the presence of well-conserved promoter regions that similarly lie within 1 kb from transcription start sites [15].

In this study, we developed a bioinformatic strategy to search for common putative regulatory motifs that might function in co-regulating V1R genes. We based our bioinformatic strategy on two assumptions. First, the V1R phylogenetic structure must be accommodated so that results are not biased by larger and more recently diverged subfamilies. Second, since transcription factor binding sites are typically degenerate, an information-based (as opposed to string-based) search methodology will be most successful at finding universally shared regulatory motifs. Many traditional motif-searching methods, such as *Meme *[22], are not designed to accommodate phylogenetic structure, and therefore in this context, tend to report motifs that arise from conservation within subfamilies we already knew about. One recently developed methodology designed to accommodate phylogenetic structure, *Phylogenetic Footprinting *[23], depends on a reliable input tree on which to evaluate whether a resulting motif is unexpected with respect to the known phylogenetic relationships established in the tree. The best use of this program, however, is in situations where the phylogeny is well established (e.g., where the input sequences are orthologs so that a well established species tree can be used). The V1R gene tree, in contrast, is poorly resolved due to the high divergence between subfamilies and frequent gene conversion events between paralogs [24]. As described below, our strategy included the development of a new motif-searching approach that, like *Phylogenetic Footprinting*, overcomes biases due to phylogenetic structure (i.e., due to different subfamily sizes and divergences), but unlike *Phylogenetic Footprinting*, is catered to the context of our problem in that it does not depend on input alignments/trees and utilizes information-based (not string-based) methods.

Our strategy to identify putative regulatory motifs involved in V1R co-regulation consisted of four steps:

1) We used comparative sequence analysis and 5' RACE to identify putative V1R promoter regions (~1 kb in size) that are well conserved within (but not across) subfamilies;

2) We aligned promoter sequences within subfamilies and represented the homology of each subfamily with a single *Sequence Logo *[25], thereby permitting a second order processing of these logos in which each subfamily is represented equally;

3) We developed a new algorithm, *LogoAlign*, which seeks an alignment of the above input logos that maximizes total information within small, motif-sized (e.g., 12 bp) windows, and therefore designed to report motifs well represented *across *subfamilies;

4) We analyzed resulting motifs on several statistical grounds, including their total information as compared to controls, and their positioning near transcription start sites as compared to random positions in gene blocks.

## Results and Discussion

### A divergent set of V1R subfamilies have arisen by local gene duplication

The mouse V1R gene repertoire consists of ~165 intact coding regions that partition into 12 major subfamilies (A-L) [12]. Most of these V1Rs (125) reside within large clusters at nine chromosomal locations [13] (see Additional File 1). Three of these V1R clusters consist of more than one subfamily: the A and B subfamilies are co-clustered on mouse chromosome 6, the J and K subfamilies are co-clustered on chromosome 7, and the H and I subfamilies are co-clustered on chromosome 13. Three V1R subfamilies are found at more than one locus: the C subfamily is found at two locations on mouse chromosome 6, the D subfamily is found at two locations on mouse chromosome 7, and the E subfamily is clustered on both chromosome 7 and 17.

**Figure 1:**
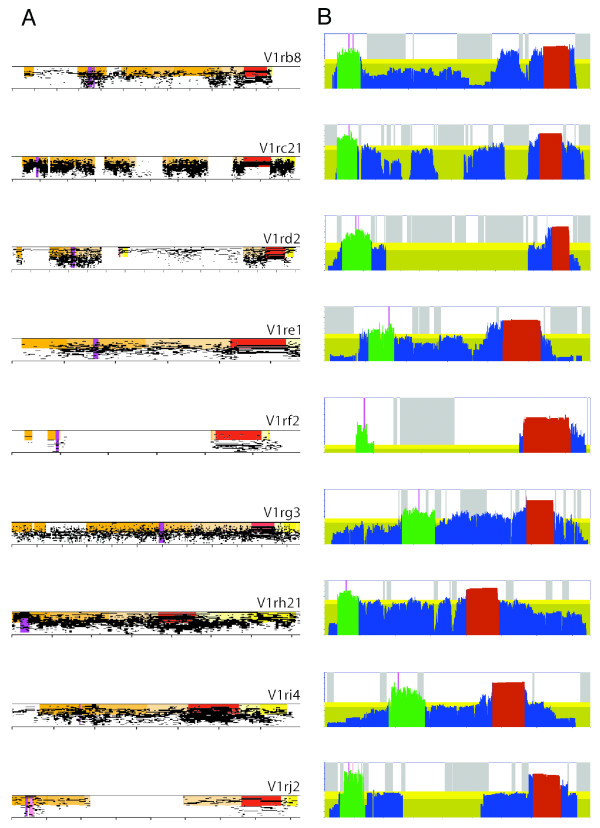
**Promoter subfamily homology in V1R gene blocks**. **A**. *PipMaker *plots [26] of V1R gene blocks showing a representative example from each promoter subfamily (subfamily is denoted in the gene name; for example, *V1rb8 *is a representative from the B subfamily). Each gene block shown was extracted from percent-identity-plots (PIPs) of self alignments of V1R loci (50–100% identity on vertical axis). Thus, the blocks shown exhibit homology at each position in the block to other V1R genes of the same promoter subfamily (i.e., at a particular position, multiple plots that cross that position indicate that multiple V1Rs in the promoter subfamily are homologous at that position). Homology to the upstream (orange), coding (red), and downstream (yellow) regions is indicated by shading. **B**. Gene block conservation scores are shown for the same representative set of V1R blocks shown in A. For each position in a gene block, the conservation score is a value between 0 and 1 (see Methods for definition of conservation scores). Coding regions (red), conservation peaks (green), and other regions of the block (blue) are shaded. Dark yellow background shading represents mean block conservation in non-repeat upstream block sequences; the light yellow band represents 0.5 standard deviations above the mean conservation score value. Grey stripes denote *RepeatMasked *positions. In both panels, homology to transcription start sites (TSS's) for each block is highlighted purple; for the V1rj2 block, an empirically determined TSS (pink), as well as homology to the TSS for V1rk1 (purple), are shown. Homologous TSS positions are derived from alignments of promoter subfamilies (see Methods). Each horizontal tick mark represents 1 kb.

V1R subfamilies have expanded by local gene block duplication [15]. Most of these local duplications occurred recently enough that the extent of the duplicated block is evident by sequence homology (Fig. 1A). Typically, duplicated blocks are about 10 kb in size; most of the duplicated sequence is upstream of the coding regions. We previously mapped the sequences of four transcriptional units from the A and B subfamilies onto their genomic blocks [15]. Each of these V1Rs had a gene structure consisting of a ~1-kb coding exon located < 2 kb from the 3' end of the duplicated block, and one or two short untranslated exons located upstream of the coding region. The putative transcription start sites (5' end of the transcriptional units) typically map < 2 kb from the 5' end of the duplicated blocks. Thus, the V1R gene block duplications seem to have efficiently captured putative promoter regions, UTRs, and coding regions, with little surrounding sequence. This observation hints that the sequence information sufficient to regulate individual V1R genes may be very proximal to transcription start sites.

### Each subfamily has conserved putative promoter regions

We previously identified putative promoter homology at the mouse chromosome-6 locus containing a cluster of V1Rs from the A and B subfamilies [15]. In this case, the putative promoter homology is shared between A and B genes. This observation raises a question as to whether this homology is universal (i.e., shared among all V1Rs), or, whether each locus might have its own promoter homology. In fact, neither seems to be the case. First, no sequence similarity is evident between A/B promoters and the gene blocks of any other subfamily. Second, not all co-clustered subfamilies exhibit shared promoter homology, as was the case for the co-clustered A and B genes: the co-clustered J and K subfamilies do exhibit cross-subfamily non-coding homology, however, neither the co-clustered H and I subfamilies or the co-clustered E and F subfamilies exhibit cross-subfamily non-coding homology. In general, we instead find that upstream non-coding homology is evident only within subfamilies, irrespective of genomic location. Thus, subfamilies distributed to multiple loci (e.g., C and D subfamilies) do exhibit non-coding homology among members at both loci (the only exception being the two clusters of E genes, which do not share obvious non-coding homology). Therefore, broad putative promoter homology is not universally shared, nor locus-specific, rather, it appears to be shared predominantly within subfamilies. For subsequent analyses, we refer to "promoter subfamilies" to indicate the ten sets of genes that share upstream, non-coding homology (AB, C, D, E1, E2, F, G, H, I, JK, where AB is the set of A and B genes, JK is the set of J and K genes, E1 is the set of E genes on chromosome 7, and E2 is the set of E genes on chromosome 17).

As a first step in identifying putative regulatory motifs shared among V1R subfamilies, we decided to restrict our search to just the putative promoter regions (and thus reduce noise by not searching entire gene blocks). As noted previously for the AB promoter subfamily [15], putative promoters in this case were evident as upstream subregions of gene blocks that exhibited increased homology. Like the AB promoter subfamily, upstream islands of non-coding, putative promoter homology are evident in other promoter subfamilies; a striking example is shown in the *PipMaker *[26] plot of the *V1rf2 *gene in Figure 1A, in which a ~500-bp island of non-coding homology is evident ~3 kb upstream of the coding region. However, for most promoter subfamilies, the gene blocks have duplicated too recently to be able to detect block subsequences that are under more stringent selection. For example, the *PipMaker *plot of the *V1rc21 *gene in Figure 1A, shows homology among C promoter subfamily members that spans the entire ~11 kb block (with breaks in homology due only to recent repeat insertions).

In order to evaluate these less obvious cases, we made a second assumption about putative V1R regulatory sequences: in addition to exhibiting higher sequence identity, these regions are likely to be homologous among all promoter subfamily members. *PipMaker *plots, like those shown in Figure 1A, contain both types of information, since the vertical axis in a plot represents percent pairwise sequence identity, and the number of plots crossing any position in the block represents the number of subfamily members with homology at that position. We transformed these two dimensions of "conservation" into a single "conservation score" at each position of each gene block. The conservation score is the average percent identity at each position based on all the *PipMaker *plots that cross that position (i.e., one plot being a pairwise comparison with one other subfamily member; see Methods); however, for each "missing gene" (i.e., for each subfamily member that does not have a *PipMaker *plot cross at that position) we include a 0% identity in this average calculation. In this way, the conservation score is severely penalized in regions where not all subfamily members exhibit homology.

Using this approach, we identify two subregions of V1R blocks that are most well conserved within promoter subfamilies (representative examples are shown in Fig. 1B; see Additional file 2 for a complete set). A prominent homology peak is generally observed across the coding regions and immediately upstream of the coding region. The non-coding portion immediately upstream of the coding region presumably includes the coding exon acceptor site, as well as translation regulatory signals. A second prominent homology peak is generally observed near the 5' end of the block, well upstream of the coding region and upstream of intervening low-homology non-coding sequence. These upstream peaks (~500–1000 bp in length) could represent promoter regions and selection of sequences containing transcriptional information.

The putative transcription start sites identified previously by 5' RACE PCR for four genes from the AB promoter subfamily map within the upstream peaks in their respective gene blocks (Fig. 1B). These four start sites are located near the middle of their respective ~kilobase-size promoter homology, but do not map exactly to homologous positions. Similarly, two previously characterized cDNAs of the D subfamily [7] initiate transcription from near the middle of their respective peaks, but not from precisely the same relative positions. Therefore, if we assume that these cDNAs accurately reveal transcription start sites (e.g., they are not derived from truncated RNA templates) and are representative cases for V1Rs in general, it would appear that V1R promoters exhibit variable relative initiation positions within these much larger regions of promoter homology.

**Figure 2:**
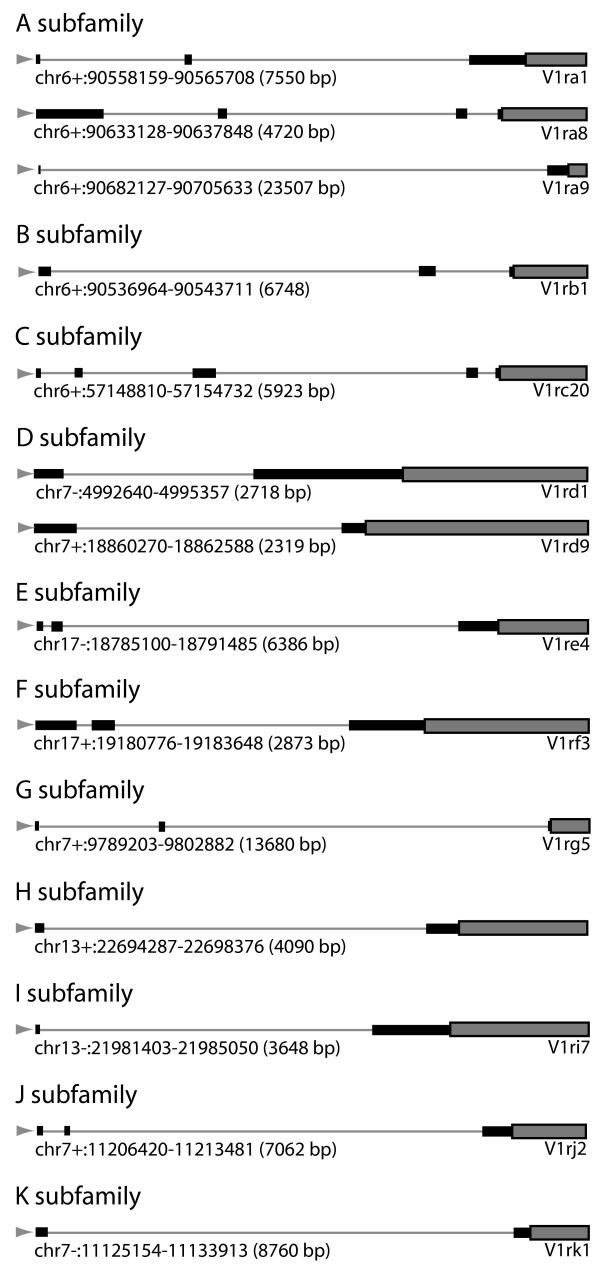
**5' Race to determine transcription start sites for representative V1Rs from each promoter subfamily**. V1R gene structures as determined by 5' RACE PCR for members of each subfamily are illustrated. Coding exons (grey-filled boxes), non-coding exons (black boxes), and upstream introns (grey lines) are denoted. The chromosomal positions represented for each V1R gene are indicated (+/- indicates orientation). Transcription start sites are at the left-most position (grey arrow). The gene structures for the AB and D promoter subfamilies were determined previously [7]; all other gene structures shown were determined herein (see Methods).

We were next interested to know whether putative transcription start sites for other promoter subfamilies likewise mapped to their respective upstream peak regions. Using mouse VNO-derived cDNA, we generated 5' RACE PCR products using gene-specific primers on at least one member of each V1R subfamily (Fig. 2). As was the case for the AB and D promoter subfamilies, transcription start sites in all cases map to upstream peaks in their respective gene blocks (Fig. 1B). We identified multiple RACE products for three V1Rs. In these cases, the putative TSS maps to within 49 bp, 41 bp, and 23 bp, respectively, with identical exon-intron gene structures. This slight variation in the length of RACE products might be due to degradation of RNA templates or alternative start sites within promoters. Nevertheless, with this limited sample, we find no evidence for alternative splicing or alternative promoters. Therefore, our comparative sequence analysis of 125 V1R genes, as well as 5' RACE results for 15 of these V1Rs, points to a single, well-conserved promoter region upstream of each gene. Further analysis of these putative promoter regions is likely to reveal promising candidate sequence elements that contribute to V1R transcriptional regulation.

**Figure 3:**
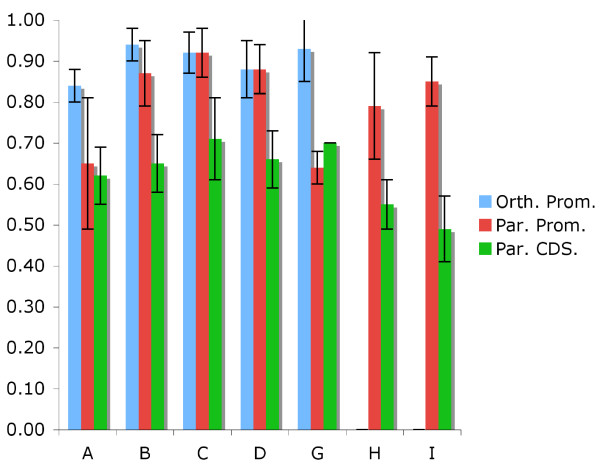
**Coding/peak versus block substitution for V1R paralogs/orthologs**. Nucleotide substitution rates within promoters of mouse paralogs (red) and mouse-rat orthologs (blue) relative to substitution rates within non-coding/non-promoter portions of gene blocks is compared to relative substitution rates within paralogous coding regions (green) for various subfamilies. Rat peaks were identified by using *BLAST [40] *to identify orthology to mouse peaks. Standard deviations in various pairwise comparisons are shown. Orthologous comparisons within the H and I subfamilies are not possible because there are no intact H and I subfamily members in rat.

We next analyzed sequence conservation within these putative promoter regions by examining nucleotide substitution rates within these regions as compared to coding and other non-coding regions. We confined our search to pairs of genes within subfamilies that exhibit non-coding block substitution rates between 15–25% so that all gene pairs considered would have approximately the same elapsed time since common ancestry. This substitution level range also permits comparisons between mouse-rat orthologous pairs, since mouse-rat orthologs are expected to exhibit 15–25% neutral substitution since the mouse-rat split [24]. We find that coding sequences exhibit average substitution levels that are ~63% that of surrounding non-coding block sequences (i.e., for every substitution observed in the block, ~0.63 substitutions are observed in the coding region) (Fig 3). We find that promoter regions ("peaks") tend to fall into two discrete classes. Most promoter sequences exhibit substitution levels that are ~80–90% that of the surrounding non-coding block sequences (i.e., for every substitution observed in the block, ~0.8–0.9 substitutions are observed in these promoter sequences). However, a subset of promoters (e.g., in the A and G subfamilies) exhibit much lower substitution levels that are comparable to levels observed in their coding regions. Interestingly, the pairs of A and G paralogous promoters that exhibit these lower substitution rates, do not exhibit lower substitution rates in orthologous comparisons (Fig. 3). This observation raises the possibility that gene conversions, which can only occur between paralogs and not orthologs, have contributed to homogenization of A and G promoters in mouse. Gene conversions might be favored in order to maintain sets of broad promoter sequences that would then compete approximately equally well for universal transcription factors. It is also noteworthy that each of the promoter regions ("peaks") exceeds 500 bp in size with few obvious breaks in homology. Such large and unbroken regions of homology seem unlikely to be the consequence of strict conservation of transcription factor binding sites, yet seem to be feasible target sizes for gene conversion events.

### Identification of cross-subfamily motifs using information-based techniques

To this point, we have identified upstream, non-coding sequences that exhibit elevated conservation scores and transcription start sites. These putative promoter regions are hereafter referred to as "peaks" (see Additional file 3 for sequences), whereas the remaining non-coding regions of a gene block are referred to as "blocks". We next searched for "motifs" shared among peaks of the ten promoter subfamilies. "Motifs" are defined in terms of nucleotide frequencies and a motif's quality is measured in terms of information content. The most common way to depict a motif is by a *Sequence Logo *[25], in which the base frequencies at each position in the motif are summarized graphically by the height of each letter. The units of a logo plot are bits of information. A motif is more information-rich (i.e., contains more cumulative bits of information) when the base frequencies across the motif deviate more from expectation (i.e., randomness). In this regard, a particular position in a set of aligned sequences need not be absolutely conserved to be information-rich. Indeed, according to information theory, a position in an aligned group of sequences that always is one of two bases can contain more information than a position that exhibits a clear majority of sequences with a single base, if in fact, the remaining sequences do not exhibit bias [25]. Thus, information profiling is a more refined method than percent identity for assessing the likelihood of a given base position containing part of a functional code. This is especially likely to be the case for transcription factor binding sites, since these sites are often degenerate and not absolutely conserved.

Some established motif-searching methods, such as *Meme *[22], are not suitable for this particular problem because they have no knowledge of the hierarchy of the dataset. Specifically, this dataset has two hierarchical levels due to very recent relationships between genes within subfamilies, yet very distant relationships between subfamilies. Thus, without knowledge of this hierarchy, algorithms like *Meme *report within-subfamily sequence identities we already knew about, as opposed to less conserved cross-subfamily homology of interest. Another motif-searching strategy, *Phylogenetic Footprinting *[23] solves this problem by utilizing an input tree that specifies the hierarchy of the dataset. However, this method is inappropriate in the context where input sequences are not homologous, too distantly related to produce a reliable phylogenetic tree, or where the phylogeny is uncertain due to concerted evolution. Since it is not possible to produce a well resolved input V1R tree, and because we wished to design our algorithm for the analysis of unrelated input sequences (e.g., co-regulated genes that are not related/alignable) and also to use information-based (versus string-based) searching, we developed a solution catered to our particular problem.

Our solution is to abstract away from individual sequences by first generating a representative *Sequence Logo *for each promoter subfamily, each logo (~500–1000 nt) being derived from an alignment of the individual peak sequences of a single promoter subfamily. This logo reflects all of the information contained within the individual peaks, yet permits a second order analysis to identify motifs shared between these representative logos, thus, across promoter subfamilies, without any one promoter subfamily being weighted more heavily (e.g., because of its size). The set of ten logos resulting from an alignment of the peak sequences for each of the ten promoter subfamilies, is provided in Additional file 4.

**Figure 4:**
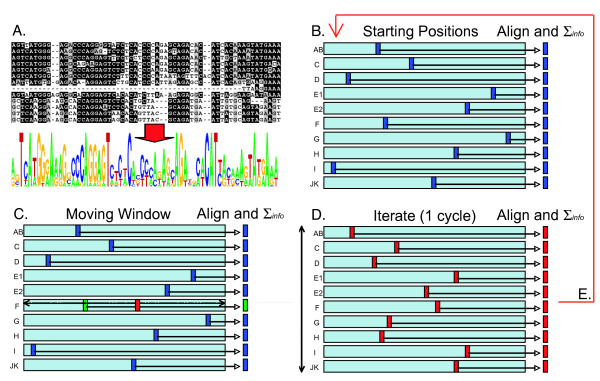
**Schematic of *LogoAlign *"hill-climbing" methodology**. **A**. Each of the ten promoter subfamilies is aligned in order to produce one input *Logo *per promoter subfamily. **B**. A random starting position of motif-sized (12-bp) windows is established for each of the input *Logos*, and total information is calculated based on the alignment of these windows. **C**. Beginning with a randomly selected *Logo*, the window is moved to every possible position in the *Logo*, and for each position, the total information is calculated based on the alignment of this window to all of the other stationary windows. The window is moved to the position at which the total information is maximized (red). **D**. In a randomly determined order, each *Logo *undergoes exactly the same window-sliding procedure as described in C above, and each time, the window is set to the position where information is maximized. One cycle is completed after every *Logo *has undergone one window-sliding procedure. **E**. Multiple cycles are executed, each time using the final resting positions from the previous cycle as starting positions for the new cycle, and each time using a random order of cycling through the ten *Logos*, until a complete cycle is completed during which none of the windows are moved. The resulting motif (a local maximum of total information) is kept as the result of one trial (2000 trials were conducted in this study).

**Figure 5:**
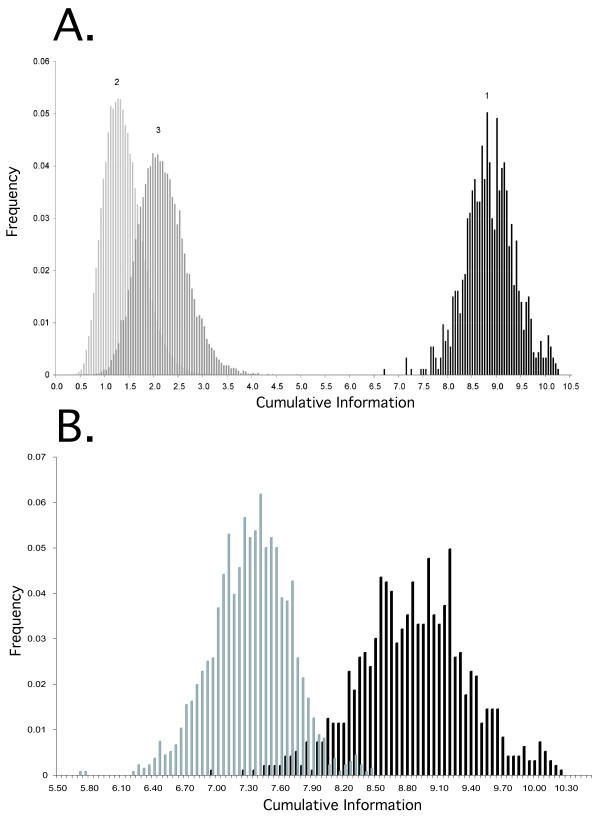
**Distributions of cumulative information in *LogoAlign *results**. **A**. The cumulative information (gap penalized) for the 936 non-redundant motifs identified by *LogoAlign *(1) is compared to 65,000 alignments of randomly chosen 12-bp windows from each input *Logo *with a maximum of 20% gaps per position per input *Logo *(2) and 20,000 biased samples in which the most information-rich 12-bp window from one randomly chosen input *Logo *is aligned to randomly chosen gap-free 12-bp windows from the other nine input *Logos *(3). The *LogoAlign *results range from 13.6 standard deviations to 22.6 standard deviations above the mean of the unbiased distribution. **B**. The distribution of results produced by *LogoAlign *(black) is compared to results from a search of random sequences (gray) with similar base composition and phylogenetic structure (see Methods). All sequences used to produce these distributions were trimmed (i.e., end-gaps were removed) in order to prevent over-accumulation of gaps within the scrambled control set. The result is bimodal (*one-tailed t-test*; *p-value *<< 0.0001), and the best motifs found within V1R promoters are significantly more information-rich than those identified in the control (*Z-score *= ~8.0; *p-value *= 10^-15^).

In order to next find motifs shared among these ten logos (thus, across subfamilies), we designed an algorithm, *LogoAlign*, that performs a heuristic search for logo similarity within short, motif-sized windows (see Fig. 4). Briefly, this algorithm begins by randomly selecting a motif-sized window (we used 12 bp) in each of the ten input logos, combines the windows into a new motif-sized logo, and then calculates the total information contained in this new motif-sized logo. The algorithm then selects one of the ten input logos, and searches all 12-bp windows in that logo and identifies the 12-bp window in that logo that produces a new motif-sized logo with the maximum of total information when aligned with the other nine stationary windows. *LogoAlign *cycles through each of the other nine logos (in a random order) in a similar manner, each time identifying the 12-bp window in the logo being processed that maximizes total information in the alignment. The algorithm continues until window positions do not move for one complete cycle. The result of one complete trial is therefore a "local maximum" of information (i.e., a maximum achieved by a heuristic path) for an aligned set of 12-bp windows; this local maximum is referred to as a "motif" (a new 12-bp logo or frequency matrix derived from the alignment of the final resting position of the ten windows). Multiple trials, each with a set of random starting positions and cycling order, identified a distribution of information-rich motifs (Fig. 5).

As described in the Introduction, *LogoAlign *was developed specifically for this particular application because it does not depend on being able to align all taxa (i.e., an input phylogeny is not required) while still being able to consider phylogenetic heirarchy in the dataset. We note that two other motif-searching algorithms have been recently developed in consideration of these same issues. The algorithm *Phylocon *[27], like *LogoAlign*, performs an information-based motif search on Sequence Logos created from multiple alignments. However, *Phylocon *uses an algorithm that we believe is suboptimal in our context for two reasons. First, *Phylocon *makes use of local, un-gapped, sub-optimal, multiple alignments, rather than gapped, global, multiple alignments. Using local alignments can be preferable if the sequences being aligned are not closely enough related to produce high-quality global alignments. However, the subfamilies in our dataset have broad contiguous regions of exceptionally high homology, with which we can produce high-quality global multiple alignments. Global alignments can provide an additional layer of information, since the existence of gaps can indicate that an otherwise well-conserved region is not likely to bind a universally-utilized transcription factor (our aim) if the motif is interrupted in a subset of sequences. Second, *PhyloCon *searches the solution space using a deterministic greedy algorithm than will always progress to the same local maximum, but may never find the best global solution. Since *LogoAlign *does not progress deterministically, it has an increased probability of identifying the best global solution with multiple iterations. A second, even newer program, *PhyloGibbs *[28] is also similar to *LogoAlign *and *Phylocon *in terms of its suitability for hierarchical datasets with incomplete phylogenies. This program uses a "simulated annealing" algorithm [29], and unlike *Phylocon*, is non-deterministic and can utilize global (or local) alignments. Thus, while *PhyloGibbs *differs from *LogoAlign *in specifics of its design, it generally approaches the motif-searching problem in a very similar way and may therefore be equally well-suited for our context. We are currently testing the relative sensitivities and computational efficiencies of several motif-searching strategies in various contexts as part of a broader assessment of *LogoAlign *(Stewart and Lane, in preparation).

### Statistical validation of candidate motifs

We assessed resulting motifs based on three criteria. First, we expect that multiple iterations of *LogoAlign *using randomly determined starting points should converge on a common set of information-rich motifs, as opposed to finding a different answer each iteration. Second, we expect that biologically meaningful motifs should contain more information than motifs identified in searches on random control sequences. Third, we expect that transcriptionally relevant motifs should more likely be positioned near transcription start sites than elsewhere in gene blocks, and/or exhibit common positioning relative to each other from gene to gene.

#### Motif Convergence

We conducted 2000 trials of *LogoAlign*, each iteration with a different starting point (i.e., initial window positions) and path (i.e., order of processing from one input logo to another). The resulting 2000 motifs could immediately be collapsed into 936 non-identical motifs (53% convergence); for example, the best motif was found 70 times. We next partitioned the 936 non-identical motifs into 64 clusters of similar motifs, allowing us to ignore weaker versions of essentially identical higher-scoring motifs. Therefore, from 2000 iterations, we observed > 97% convergence to merely 64 non-redundant solutions, suggesting that we might have sufficiently sampled the sequence space to find a global maximum.

#### Significant Information Content

We conducted three control experiments to assess the significance of the total information of V1R promoter motifs identified by *LogoAlign *(Fig 5). The first control establishes the noise level for randomly aligned sequences; the maximum total information in this case is ~3 bits, whereas the minimum information context in the 2000 iterations of *LogoAlign *has more than double this information content. Not surprisingly then, *LogoAlign *always progresses towards a more information-rich solution. The second control establishes the relative contribution of individual subfamilies. In this control, we establish that a biased distribution in which the most information-rich window from one logo is aligned with randomly selected windows from the other nine logos produces motifs only slightly above noise and significantly less than the information content in the experimental results.

The most important control is to ask how *LogoAlign *performs on random sequences. To conduct this control, we shuffled V1R sequences not only in such a way to preserve base composition, but also to preserve the number, size, and diversity of subfamilies (see Methods). Thus, our control was a set of sequences with exactly the same properties as the experimental set, only with putative motifs scrambled. We find that the distribution of information in the control set versus the experimental set is bimodal (*1-tailed t-test*, *p-value *<< .0001), indicating that motifs identified in V1R promoters are more information-rich than those identified in random sequences. Note that while the majority of motifs identified in V1R promoters contain more information than the maximum information-rich motifs identified in the control, we presume based on the overlap of these distributions that the least information-rich V1R promoter motifs identified in this study are probably not biologically significant (i.e., contain an amount of information expected to occur by chance in random sequences). However, the best motifs (> 10 bits) contain significantly more information than those found in the control (*p-value *= ~10^-15^), and ~81% of identified V1R promoter motifs contain information that is > 3 standard deviations above the mean for the control (*p-value *< 0.001) (Fig. 5B).

#### Non-random positioning

**Figure 6:**
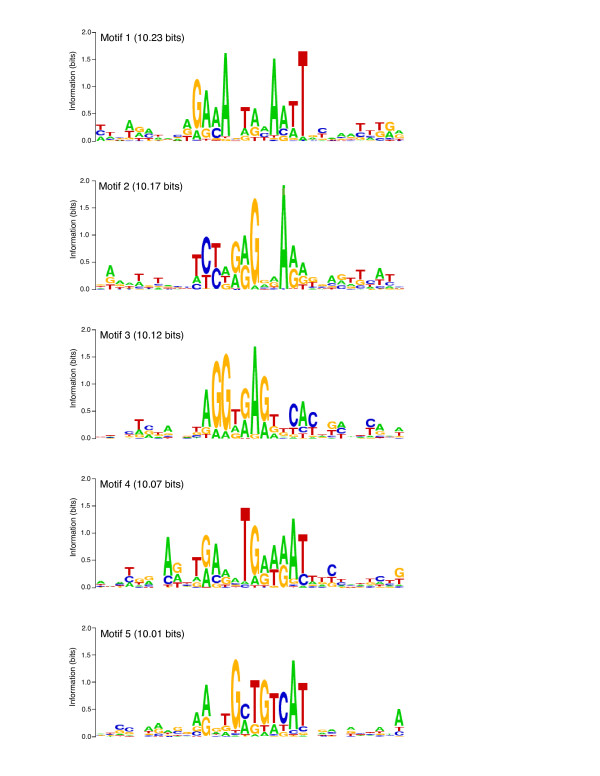
**Sequence *Logos *for the top five motifs found by *LogoAlign***. Sequence *Logos *for the five motifs with total information > 10 bits (gap penalized; see Methods) are shown. The motif (underlined) is shown in context with the surrounding alignment of the input *Logos *to illustrate the motif signal within background noise.

**Figure 7:**
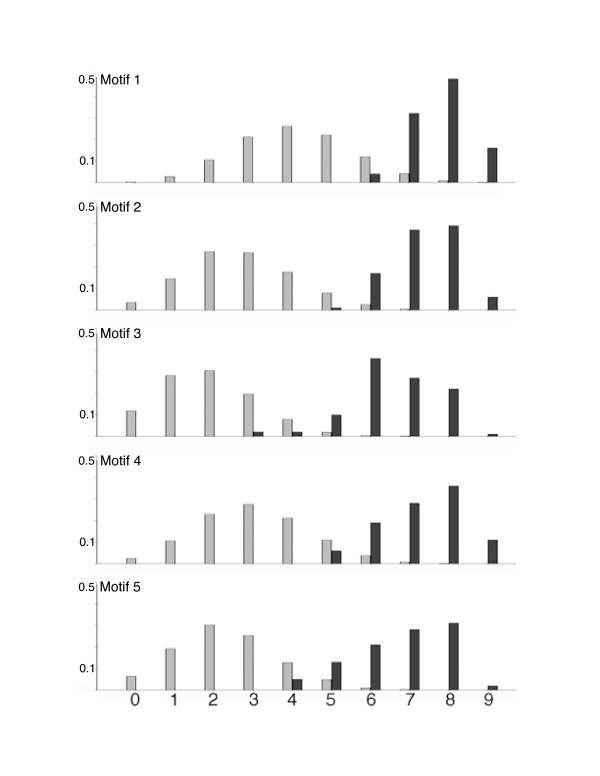
**Frequency of occurrence of motifs near transcription start sites (TSS's)**. For each of the five motifs, we compared the frequency of the motif in randomly-selected 9 gene samples (1 gene from each promoter subfamily) occurring within 250 bp of putative TSS's (black) versus random 500-bp windows in gene blocks (grey). A total of one thousand 9-gene samples were taken to produce the distribution (see Methods). The frequency of random occurrence in gene blocks for Motif 1 is higher (and less resolved from the test set) because Motif1 is especially AT-rich, and thus is expected to occur more frequently by chance in gene blocks, which are also AT-rich. The five motifs are present near TSS's in an average of 7.1 genes per 9-gene cross-subfamily sample; background frequency of the five motifs within gene blocks average 2.8 genes per 9-gene cross-subfamily sample.

We next analyzed motif occurrences within V1R gene blocks using *Sequence Walking *techniques [30]. The rationale for this test is that some sequence motifs (e.g., AT-rich motifs), even if information-rich among V1Rs, might occur frequently in background sequence (e.g., V1Rs reside in AT-rich isochores with an average of ~62% AT-base composition). Therefore, some statistical measure is appropriate to ascertain that identified motifs are enriched specifically within V1R promoters, as opposed to random widespread occurrence elsewhere in the gene blocks. For this analysis, we focused on the five best motifs (Fig. 6). Generally, we find that these motifs tend to be located in better conserved parts of peaks, and the motifs found within peaks tend to be better conserved than motifs found elsewhere in gene blocks (not shown), suggesting that occurrence in promoter regions, but not in non-peak portions of blocks, are under the most stringent selective pressure. We find that these motifs occur within 500 bp of putative transcription start sites with significantly greater frequency than within random 500 bp windows of their gene blocks (Fig. 7). Note that this latter analysis assumes that putative transcription start sites in conserved V1R promoter subfamilies are located at homologous positions to empirically-determined start sites determined by RACE (see Methods). As discussed previously, while this is probably not precisely the case, the 500-bp window used in this analysis seems sufficiently large to accommodate the expected variability in relative start positions. If all V1R transcription start sites were more precisely known, we expect we would observe an even greater relative enrichment of motif occurrences near TSSs, since this knowledge would presumably permit the use of a smaller window size in the analysis.

We next focussed on only those 15 V1R genes in which transcription start sites were empirically determined by RACE in order to investigate more subtle patterning of our five most information-rich motifs identified in the study. The most compelling observation is that the highest-scoring occurrence of *motif 3 *overlaps the 5' exon-intron junction for 7 of 15 V1Rs where gene structures were determined by RACE, suggesting that this motif contains splice donor information for these upstream exons. In one case (V1R *H13.22697*) of the 15 RACE products analyzed, *motif 1 *is located precisely at the empirical transcription start site; however, for the other V1Rs, the best version of this motif is found as far as 413 bp upstream and 731 bp downstream of empirical transcription start sites (median distance is 90 bp upstream of the TSS), and therefore it seems unlikely that this motif generally contains transcription initiation information. If *motif 3 *(putative donor splice site) is excluded, the best occurrence of the other four motifs is found just upstream of empirically-determined transcription start sites in 33/48 cases (~69%), within the 5' exons in 7/48 cases (~15%), and within the upstream intron (downstream of both the TSS and 5' exon) in 8/48 cases (~17%); therefore, it seems likely that these motifs generally contain transcriptional information, as opposed to, for example, exon or splicing information.

We also explored the possibility that pairs of motifs might require critical spacing with respect to each other. We do find examples of motif pairs that exhibit surprisingly similar spacing across the promoters of multiple subfamilies. For example, the spacing between *motif 2 *and *motif 4 *is consistent within 5 bp for 39 different V1Rs across five subfamilies. However, we do not observe conserved relative spacing that encompasses all, or even nearly all V1Rs, suggesting that stringent spacing among motifs tested is not required, as might be predicted for binding sites of transcription factors cooperating/interacting in a regulatory complex.

Finally, we compared our five most information-rich motifs to a library of known transcription factor binding sites (Matrix Family Library Verson 6.3) [31]. The best occurrences of *motifs 1 *and *3 *within V1R promoters frequently contain an "AAGTT" that matches the core binding site for the *MYT1 *zinc finger transcription factor; however, only ~a third of these motifs are recognized by the Genomatix algorithm [31] as a sufficient match to the *MYT1 *site. In general, while we find that our motifs bare resemblance to a number of transcription factor binding sites represented in the database, we do not identify a compelling example in which the conserved core of a binding site is also a conserved core within any of our five best motifs. Nevertheless, since few transcription factors have been investigated by ChIP-on-chip experiments to fully explore the range of actual binding sites in the genome, and indeed, in cases where transcription factor binding in the genome has been characterized in this comprehensive way, it appears bound sites can deviate significantly from consensus sequences [32], we only very tentatively suggest that our five motifs do not resemble known transcription factor targets.

## Conclusion

In summary, we have used a combination of existing and new bioinformatic tools to discover that every V1R subfamily has a broad and well conserved promoter region that contains empirical transcription start sites. We speculate that the high level of conservation over such a large sequence territory might have resulted from gene conversions that would tend to broadly homogenize these sequences. Homogenization in turn, could permit all V1Rs of a particular subfamily to compete equally well for common transcription factors or complexes. We have developed a new information-based motif-searching strategy that permits sequence comparisons between different subfamilies (and eliminates bias due to subfamily over-representation), but is not dependent on the alignment of sequences across subfamilies, in order to identify candidate transcription factor binding motifs that are represented in all V1R promoters and present across all V1R subfamilies. The best motifs contain > 10 bits of cumulative information, which is significantly more information than those identified in random sequences. Three of these motifs have relatively high GC content (46%, 47%, and 44% in motifs 2, 3, and 5, respectively, as compared to 38% GC content in background sequences at these loci), and the best motif is identified near transcription start sites in 94% of all V1R promoter regions (108/115 V1Rs in which promoter peaks and transcription start site homology was identified; see *Boxshade *[33] alignment of motif occurrences in Additional file 5). These observations suggest that these motifs are excellent candidate binding sites for V1R co-regulatory factors, a hypothesis we are now testing experimentally.

Our algorithm, *LogoAlign*, has potential for use in a broader context of gene co-regulation. Two widely used approaches, exemplified by *Meme *and *Phylogenetic Footprinting*, are appropriate in distinct contexts. *Meme *(and similar approaches) is best used when analyzing sequences of comparable distances (e.g., unrelated sequences), but is not suitable when there is varying degrees of homology within the sequence set, because it does not correct for phylogenetic relatedness. *Phylogenetic Footprinting *is best used when analyzing sequences with varying degrees of homology (it is designed to correct for phylogenetic relatedness), but is not suitable when some of the sequences are unrelated, because it depends on an input tree that establishes phylogenetic relationships for all input sequences. Our algorithm, *LogoAlign*, as well others such as *PhyloCon *and *PhyloGibbs*, applies to a niche where there exists both varying degrees of homology *and *sets of unrelated sequences for which no phylogeny can be established. An example of this particular context would be in the evaluation of regulons elucidated by microarray studies, where both related and unrelated target genes may be part of a downstream battery of genes under the control of a particular transcription factor. We are currently conducting a broader comparative evaluation of the *LogoAlign*, *Phylocon*, *PhyloGibbs*, and other related motif-searching strategies using synthetic controls in order to more rigorously assess relative sensitivities and context-dependent efficiencies of these various algorithmic approaches.

## Methods

### Genome sequences

All genome sequences were taken from the UCSC Genome Browser [34]. Mouse V1R genome sequences are taken from the March, 2005 assembly and rat sequences are from the June, 2003 assembly. The 125 mouse V1R intact coding sequences from 12 subfamilies (designated A through L) within the 9 major genome clusters used in this study were identified previously [12]. The complete list of V1Rs, subfamily designations, and chromosomal locations is shown in Additional File 1.

### 5' RACE PCR and estimation of transcription start sites for all V1R genes

RNA was isolated from vomeronasal tissue dissected from two-month old mice using Trizol (Invitrogen). RACE-ready cDNA was prepared using the BD SMART RACE cDNA Amplification Kit (BD Biosciences). V1R primers were designed against sequences that are well conserved within subfamilies, in order to increase the likelihood of obtaining RACE products for multiple V1Rs in a single PCR reaction. The following (GSP1) primers were used to target genes from V1R subfamilies (in some cases, a second nested primer, GSP2, was necessary):

C-subfamily GSP1 5'-GTCAGTTGACAGGAGATCAGGTCTGTGGG-3'

D-subfamily GSP1 5'-AGGAGAATGAAGGTATTGGCCACAGC-3'

E subfamily GSP1 5'-CTGATGGTGATGGCCTGGAAGACACTCAA-3'

F subfamily GSP1 5'-CACAGTAGAGTTAAGAAGTTGGCTACAAGC-3'

F subfamily GSP2 5'-CTTTAGTCTGCACCTCATGTAGTAAAGGC-3'

G subfamily GSP1 5'-CAAGTGCTCTATGATCAGGTCTTTGGGCGC-3'

H subfamily GSP1 5'-GAAGGATTAGGATGTTTGTAAAAGCCAAGTG-3'

I subfamily GSP1 5'-GTGTTTACAAAAGCCAAGTGGATGAGAAT-3'

I subfamily GSP2 5'-CCCATGATGAAAGTATATAAATGTCTCACAAATAGC-3'

J subfamily GSP1 5'-GTTGACATATTAACTATTGTCAGGTGCAT-3'

J subfamily GSP2 5'-GGTGCATAAAAACTGAATCTATCAAC-3'

K subfamily GSP1 5'-CATTGACAAGCATTAGATGTGTGAAGATC-3'

L subfamily GSP1 5'-GTCATTGTCTGAGGGATTCCTTTGCAGAG-3'

L subfamily GSP2 5'-CTATAGGCCTTGTCCTGACCCCAGTGAGC-3'

5' RACE products were successfully obtained and sequenced for the C, E, F, G, H, I, J, and K subfamily members (but not D and L). To infer transcription start sites for representatives of the D subfamily, we used previously mapped RNAs for the *V1rd1 *and *V1rd9 *genes [7]. Representative RACE products are shown in Figure 2. RACE products not shown in Figure 2 include cDNA sequences that do not exactly match a genome sequence (e.g., we identified a C subfamily member with 96% identity to V1rC32), or represents an unannotated V1R gene (e.g., we identified a three-exon E subfamily cDNA that maps to chr17:18290161–18295277; we identified a four-exon G subfamily cDNA that maps to chr7:10215569–10220549). In these cases not shown, the putative TSS maps to within respective homology peaks (see below), as is the case for all RACE products shown in Figure 2. In most cases, only a single clone was isolated for each V1R, however, multiple independent clones were identified for V1Rk1 (2 clones), the unannotated H-subfamily V1R at chr13:22694287–22698376 (3 clones), and the unannotated E-subfamily V1R at chr17:18290161–18295277 (3 clones).

### Inferring Transcription Start Sites (TSSs) for all V1R genes

As described above, we mapped 5' RACE-determined TSSs for at least one member of each subfamily. We extrapolated TSSs from V1Rs whose TSS was empirically determined by RACE to homologous positions within well-conserved promoters of other V1Rs of the same subfamily. The error in predicting actual TSS positioning using this method might possibly be substantial (> ~100 bp), since we observe that even well-conserved V1R promoter regions can initiate transcription from different relative start positions (see "Each subfamily has conserved putative promoter regions" discussion in text). Therefore, our analyses of relative positioning of motif occurrences is conducted at low resolution (i.e., using 500-bp windows, see below) in order to accommodate this expected error in TSS extrapolation

### Defining gene blocks and homology "peaks"

We used the *PipMaker *tool [26] to detect pairwise sequence homology within subfamilies, as opposed to a multiple sequence alignment program, in order to maximize sensitivity and to detect the largest extent of block of homology between genes. For each position in each gene block, we parsed the *PipMaker *plots to derive a promoter subfamily "conservation" score of that position with a value between 0 and 1. These scores represent the average percent identity of the position to corresponding positions in the blocks for all other members of the promoter subfamily; if the position did not align with the block of a promoter subfamily member, a 0% identity (strong penalty) was used for that particular position in the average. Thus, a high "conservation" score indicates both a high percent sequence identity with other promoter subfamily members at that position as well as a tendency for this homology to be present in all other promoter subfamily members.

An arbitrary method was empirically derived in order to generate systematic peak definitions that agreed well with obvious peaks as identified by eye. First, we calculated a moving average of conservation values across 500-bp windows and identified a global maximum value (peak center) in the block. Next, peak shoulders were defined as the first position, both upstream and downstream of the peak center, that satisfy the following three criteria: 1) the conservation score at the position is below threshold, 2) the 500-bp moving average around the position is below threshold, and 3) 50% or more of the conservation scores within 50 bp of the position are below threshold; the threshold in all three criteria is defined as the value 0.5 standard deviations above the mean conservation score for the entire non-repeat block homology upstream of the coding region. A complete set of V1R gene blocks showing peak definitions are provided in Additional file 2. For almost all genes, peaks were homologous and alignable from gene to gene within promoter subfamilies; for ten genes, no peaks were identified or peaks were identified that were not homologous to other peak sequences in the promoter subfamily, and these sequences were excluded from initial motif searches. Peak sequences (typically ~500–1000 bp in length) from each promoter subfamily were aligned using *ClustalW *[35] with default parameters.

### The LogoAlign algorithm

The alignments of "peak" sequences from each promoter subfamily were used to derive one *Sequence Logo *[25] per promoter subfamily. We used these *Logos *as inputs to a new algorithm, *LogoAlign*, that finds an alignment of the input *Logos *that maximizes cumulative information over short, motif-sized windows. The algorithm proceeds in the following manner. First, a random window position is selected for each input *Logo *and the cumulative information of the aligned windows is calculated for this initial alignment. To compute the cumulative information, the base frequencies at each position in the motif-sized windows are averaged to derive a new set of base frequencies, and then the information at each position is computed using this new frequency matrix. The cumulative information of the alignment is calculated as the sum of the information values at each position in the motif-sized windows. Next, starting with one of the *Logos*, its window is moved to each possible position and the cumulative information is recomputed with respect to this window and the stationary windows in the other *Logos *(effectively, the *Logo *is shifted to all possible positions relative to the other stationary *Logos*, and the cumulative information is calculated for each position). The highest scoring window is assigned as the new window position for that *Logo*. The moving window procedure is repeated in turn for each of the other *Logos*, each time assigning a new window position that maximizes cumulative information in the window alignments. One cycle of the algorithm is completed once each input *Logo *has undergone the window sliding procedure. The algorithm then begins a new cycle (with a different, randomly determined order through the ten input *Logos*). This cycling continues indefinitely until an entire cycle is completed in which none of the window positions move. The final window positions on the *Logos *give a local maximum of cumulative information that is retained as a motif. This "hill-climbing" approach, summarized in Figure 4, is similar to the one used in the *Malign *algorithm [36].

To search for our candidate motifs, we ran 2000 trials of the *LogoAlign *algorithm with random initial seed alignments. In our study, the dataset reported was generated using a 12-bp window, having previously explored a range of window sizes between 8–16 bp and empirically establishing that a 12-bp window was sufficient to capture the best motifs. The 2000 iterations resulted in 936 non-identical motifs. To further summarize this set as a smaller set of representative motifs, we employed a greedy clustering algorithm based on defining a *Manhattan distance *between base frequencies for pairs of motifs (similar to "*Quality Threshold*" clustering methods; [37]). We empirically defined a clustering radius so that the algorithm tended to group *Logos *that had the same most-frequent bases at aligned positions (i.e., the "same" motifs, as evident by eye). Clustering resulted in the 936 non-identical motifs being partitioned into 64 clusters.

The *LogoAlign *motif searching algorithm we used was only one of several motif elicitation techniques we developed and utilized on the V1R peak sequences. One alternative approach was a variant of *LogoAlign *that used a greedy search algorithm similar to the methodology in *Consensus *[38], as opposed to the hill-climbing technique described above. Furthermore, we ran *LogoAlign *with different parameters, such as using relative entropy (that takes background base composition into consideration) instead of information. Neither of these variations to *LogoAlign *significantly altered our results.

### Evaluating candidate motifs for information content

We evaluated total information content of resulting motifs as compared to three controls. First, to estimate background noise level for total information of motif size 12 basepairs, we generated 65,000 alignments of windows randomly selected from the input *Logos *(permitting a maximum of 20% gap frequency per position in the input *Logos*; 20% gap tolerance allows for one gap in the *Logos *for the smallest promoter subfamilies that contain only five peaks) (Fig. 5A). Second, to estimate the contribution to total information from any one subfamily, we produced a biased set of 20,000 *Logo *alignments in which each sample contained the window of highest cumulative information for one randomly selected input *Logo *aligned with gap-free random windows from the other nine *Logos *(Fig. 5A). Third, we ran *LogoAlign *on a set of random sequences in order to investigate the level of information in motif occurrences that arise from chance (Fig. 5B). For this control, we generated the random sequences by scrambling V1R peak alignments (with end-gaps trimmed) in order to preserve base composition. Our method of scrambling involved systematically shuffling every gapped peak-sequence within a subfamily in exactly the same way, so that even though all motifs would be disrupted, the phylogenetic relationships within subfamilies were maintained. Therefore, the randomized control sequences used for this test consisted of the same gene numbers, subfamily sizes, percent identities, and base composition as the experimental set.

### Evaluating candidate motifs for positional bias

We investigated the biological significance of the five most information-rich motifs by asking three questions pertaining to the occurrences of these motifs within gene blocks: 1) Are motifs more likely to occur near transcription start sites as compared to elsewhere in gene blocks?; 2) Are motif occurrences generally well conserved portions of gene blocks?; 3) Are motif occurrences similarly positioned from gene to gene with respect to each other or to transcription start sites? To address each of these questions, we first analyzed motif occurrences by applying a *sequence walking *method [30] to scan each V1R gene block for sequences that closely matched the best motifs. We arbitrarily defined an occurrence of a motif as a sequence with at least 9 bits of *individual information *[39]. To determine the frequency of motif occurrences near transcription start sites (TSS's), we took 100 samples each containing nine genes, with one gene randomly selected from each of nine promoter subfamilies (the E2 promoter subfamily was excluded because E2 TSS's have yet to be identified), and counted the number of genes in the 9-gene sample that had a motif occurrence within 250 bp of the TSS in either direction (500-bp vicinity). This sampling approach permits an analysis of the likelihood that a motif is proximal to TSS's without introducing bias arising from large, well-conserved subfamilies that might lead to an over-estimation of this probability. To determine the frequency of occurrences in the control sets, we took 10000 samples each containing nine genes, one randomly selected from each promoter subfamily, and counted the number of genes in the 9-gene sample that had a motif occurrence within a randomly selected 500-bp window within their gene blocks (i.e., non-coding, non-promoter regions). Results from this analysis are shown in Figure 7. To investigate a correlation between motif occurrences and conservation within gene blocks, we calculated average conservation scores (and standard deviations) within blocks/peaks, and calculated *Z-scores *for motif occurrences within blocks/peaks. To investigate whether motif occurrences exhibit similar relative spacing (+/- 5-bp tolerance) from gene to gene, we tabulated all pairs of motif occurrences (and motif occurrences relative to TSS's) for every possible relative positioning (between -500 to +500 bp).

## Authors' contributions

RPL conducted all comparative genome sequence analyses and 5' RACE PCR, and provided conceptual direction in the development of bioinformatic approaches. RS developed, tested, implemented, and applied the LogoAlign algorithms used in this study. Both authors wrote, reviewed, and approved the final manuscript.

## Supplementary Material

Additional file 1 ***Table of V1R gene names, locations, and subfamily designations***. Excel spreadsheet itemizing all V1Rs used in this study. Click here for file

Additional file 2 **Conservation scores, peak definitions, putative transcription start site locations, and motif occurrences for the complete set of V1R gene blocks**. Gene block conservation scores and sequence walks for 124 intact V1R genes are found at . To view a gene block, first select a motif directory, then click on a V1R gene name (e.g., "V1ra1"). Coding regions (red), conservation peaks (green), and other regions of the block (blue) are shaded. Gray stripes denote RepeatMasked portions of the gene block. Empirical TSS's determined by 5' RACE are shaded light blue; homology to transcription start sites determined for another subfamily member are highlighted pink. Each horizontal tick mark represents 500 bp. Sequence walker motif occurrences are indicated by vertical lines with the height of the lines indicating individual information of each motif occurrence. Click here for file

Additional file 3 ***V1R peak sequences***. Text file in *fasta *format of all putative promoter regions ("peaks") for all V1R sequences used in this study. Click here for file
