# Supplementary material for: V1R promoters are well conserved and exhibit common putative regulatory motifs
Source: BMC Genomics. 2007 Jul 25;8:253. doi: 10.1186/1471-2164-8-253 (PMC1955453; doi:10.1186/1471-2164-8-253)
Supplement: Additional file 2 — Conservation scores, peak definitions, putative transcription start site locations, and motif occurrences for the complete set of V1R gene blocks. Gene block conservation scores and sequence walks for 124 intact V1R genes are found at . To view a gene block, first select a motif directory, then click on a V1R gene name (e.g., "V1ra1"). Coding regions (red), conservation peaks (green), and other regions of the block (blue) are shaded. Gray stripes denote RepeatMasked portions of the gene block. Empirical TSS's determined by 5' RACE are shaded light blue; homology to transcription start sites determined for another subfamily member are highlighted pink. Each horizontal tick mark represents 500 bp. Sequence walker motif occurrences are indicated by vertical lines with the height of the lines indicating individual information of each motif occurrence. [file 1471-2164-8-253-S2.pdf]

V1ra1	1	AGTCACTAAAATT-NNNNNNNNNNNNN-TGAGTAAGTGCCC-TGAGGAGAAAAT-NNNNNNNNNNNN
V1ra2	1	AGTCACTAAAATT-NNNNNNNNNNNNN-TGAGTAAGTGCCC-TGAGGAGAAAAT-NNNNNNNNNNNN
V1ra5	1	AGTCACTAAAATT-NNNNNNNNNNNNN-TGAGTAAGTGCCC-AGAGAAGGAAAT-NNNNNNNNNNNN
V1ra6	1	AGTCACTAAAATT-NNNNNNNNNNNNN-TGAGTAAGTGCCC-AGAGAAGAAAAT-NNNNNNNNNNNN
V1ra7	1	AGACACTAAACTT-CCCAGGGAAAGGC-NNNNNNNNNNNNN-NNNNNNNNNNNN-NNNNNNNNNNNN
V1ra8	1	AGACACTAAACTT-NNNNNNNNNNNNN-NNNNNNNNNNNNN-NNNNNNNNNNNN-GATAGCTGTTAT
V1ra9	1	GAAGAATAAAATT-CCCAGGGAGAGGC-TGGGTAAGTGCCC-NNNNNNNNNNNN-GATGGCTGTCAT
V1rb1	1	TAAAATAACAAAT-NNNNNNNNNNNNN-TGGGTAAGTGTCC-TGCAATGGAGAC-NNNNNNNNNNNN
V1rb2	1	AGACACTAAAATA-TCTAAAGTAAATG-TGGGTAAGTGTCC-TGCAATGGAGAC-GATTGCTGTTAT
V1rb3	1	NNNNNNNNNNNNN-NNNNNNNNNNNNN-NNNNNNNNNNNNN-NNNNNNNNNNNN-NNNNNNNNNNNN
V1rb4	1	AGAAACTAAAATT-ATCAAAGGAAAGG-TAGGGAAGTTTTC-NNNNNNNNNNNN-NNNNNNNNNNNN
V1rb7	1	GGACACTAAAATT-NNNNNNNNNNNNN-TTGGTAAGTGTCC-NNNNNNNNNNNN-GATGGCTGTTAT
V1rb8	1	AGACAGTAAAATT-NNNNNNNNNNNNN-TGGGTAAGTTCTC-TGCAATGGAGAA-GATGGCTGTCAT
V1rb9	1	AGACACTAAAATT-NNNNNNNNNNNNN-TGGGTAAGTGTCT-TGCAATGGAAAA-NNNNNNNNNNNN
C6.57316	1	TGACACTAAAAGT-TCTAGAGGGAGAC-TAGGGAAGTTCAC-TGACATGATGAC-AACTGATTACAT
C6.57589	1	GGAAAGTTCACTT-TGTAGAGGGAGAT-TAGGAAAGTTCAC-TGAGATAATAAC-NNNNNNNNNNNN
C6.58635	1	GGAAAGTTCACTT-CCTAGAGAGAAAA-TAGGAAAGTTCAC-NNNNNNNNNNNN-NNNNNNNNNNNN
V1rc1	1	GGAAAGTTCACTT-TCTAGAGGGAGAT-TAGGAAAGTTCAC-TAAGATGAAGAT-ACATGATGACAT
V1rc16	1	GGAAAGTTCACTT-TGTAGAGGGAGAT-TAGGAAAGTTCAC-TGAGCTAATGAC-NNNNNNNNNNNN
V1rc17	1	TGACATTATAATT-TCTAAAGGGAGAT-TAGGAAAGTTCAC-AGAGCTGTTAAT-AGGAGGTGACAT
V1rc18	1	GGGAAGTTCACTT-TCTAGAGAGAGAT-TAGGGAAGTTCAC-TGAACTGGTGAT-NNNNNNNNNNNN
V1rc19	1	GGGAATATAAATT-TCTGGAGGGAGAT-TAGGGAAGTTTAC-TGAAGTGATAAC-ACCTGATGACAT
V1rc20	1	GGGAAGTTCACTT-TCTAGAGGGAGAT-TAGGGAAGTTCAC-TAAGCTGAAGAT-AGCTGATGGCAT
V1rc21	1	GGAAAGTTTACTT-TCTAGAGGGAGAT-TAGGAAAGTTTAC-TAAGCTGAAGAT-AGGAGCTGTTAC
V1rc22	1	TGAAAATACAGTT-TCTAGAGGGAGAT-TAGGAAAGTTCAC-TGAGCTGATAAT-AGGAGGTGACAT
V1rc23	1	AAAAAAAATAATT-TCTAAAGGGAGAT-TAGGGAAGTTCAC-TGAGCTAGTGAT-NNNNNNNNNNNN
V1rc25	1	GGAAAGTTCACTT-TTTTAAGTGAAAT-TAGGAAAGTTCAC-NNNNNNNNNNNN-AGCTGATGCCAT
V1rc26	1	GGAAAGTTCACTT-TTTTAAGTGAAAT-TAGGAAAGTTCAC-NNNNNNNNNNNN-CACTGAAGTCAT
V1rc27	1	AGAAAGATTACTT-NNNNNNNNNNNNN-TAGGGAAGTTCAC-NNNNNNNNNNNN-AACTGATGACAT
V1rc28	1	TGACATGATAATT-TCTAGAGAGAGAT-TAGGTGAGACCCC-TGACATGATAAT-AAGAGCTGTTAT
V1rc29	1	GGGAAGTTAACTT-TCTAGAGGGAGAT-TAGGAAAGTACAT-TGAACTGATGAT-AACTGATGATAT
V1rc3	1	AGACAAAAATATT-TCTAAAGGGAGAA-TAGAGAAGTTTAC-AGAGCTGATGAC-AGCTGATGACAC
V1rc33	1	GGAAAGTTCACTT-TGTAGAGGGAGAT-TAGGAAAGTTCAC-TGAGATAATAAC-NNNNNNNNNNNN
V1rc5	1	GGAAAGTTTACTT-TCTTAGGAAAGTT-TAGGAAAGTTTAC-TGAGCTGATGAC-AGCTGATGACAT
V1rc6	1	GGAGAGAATAATT-ACTAGAGGGAGAT-TAGGGAAGTTCAC-TGACATGGTAAT-NNNNNNNNNNNN
V1rc7	1	AGAAAGAATACTT-TCTAGAGGGAGAC-TGGGGAAGTTCAC-TGACATGATAAC-AGCTGATGACAT
V1rc8	1	TGAAAATACAGTT-NNNNNNNNNNNNN-TAGGAAAGTTCAC-TGAACTGATAAC-AACTGATAACAT
C6.66890	1	TGACACCATAATT-TCTAGAGGGAGAT-TAGAGAAGTTCAC-TGAGCTGATGAC-AGCTGATGACAT
V1rc10	1	AGAAAGATAACTT-TCTAGAGGGAGAT-TAGGGAAGTTCAC-TGAGCTGATGAC-AGCTGATGACAT
V1rc12	1	TGACAATACAGTT-TCTAGAGGGAGAT-TAGGGAAGTTCAC-TGAGCTGATGAC-AGCTGATGACAT
V1rc14	1	TGACAGTACAGTT-TCTAGAGGGAGAT-TAGGGAAGTTCAC-TGAGCTGATGAC-AGCTGATGACAT
V1rc15	1	AGAAAGATAACTT-TCTAGAGGGAGGT-TAGGGAAGTTCAC-NNNNNNNNNNNN-AGGAGATGTTAT
D7.18707	1	AGAAAGTGAAAAT-TCTGGGGATAAAT-TAGGTGAGATTCC-GAAAATGATGAT-GAGTGATGTCAG
D7.18779	1	AGACACAATAAAT-TTTTAGGGAAAGT-TAGGTGAGATTCC-GAAAATGATGAT-AAAGGATGTGAT
V1rd13	1	AGAAAATAAAAAT-TCTGGGGATAAAT-TGGGAAAGATCAG-GAAGCTGGAGAT-GGAAGATGTCAT
V1rd16	1	TGGAACTTTAATT-TCCAGAGAGAAAA-GAGGAAAGTACTC-NNNNNNNNNNNN-GAAGGATGTCAT
V1rd17	1	TGAAAGTAAAAAT-TCTGGGGATAAAT-NNNNNNNNNNNNN-GAAGGTGAAAAT-AGGAGCTGTTAT
V1rd18	1	AGAAAGTAAAAAT-TCTGGGGATAAAT-NNNNNNNNNNNNN-AAAAATGGAGAC-GAAGGATGTCAT
V1rd20	1	AGAAAGTAAAAAT-TCTGGGGATAAAT-NNNNNNNNNNNNN-AAAAATGGAAAT-GAAGGATGTCAT
V1rd9	1	AGAAGGTAAAAAT-TTCAAAGGAAGAC-CAGGTGAGATTCC-AAAAATAGAGAT-GAAGGCTTTCAT
V1rd1	1	AGGAAGTGAAAAT-TTCAAAGGGAGAG-AAGGTGAAAGCAT-GGAAGTGAAAAT-NNNNNNNNNNNN
V1rd11	1	AGAAAGTGAAAAT-TTCAAAGGGAGAG-NNNNNNNNNNNNN-GAAAATGGTGAT-CAATGATTTCAT
V1rd2	1	AGGAAGTGAAATT-TTCAAGGGGAGAG-AAGGTGAAAGCAT-TAATATAATAAT-AGGAGCTGGCAT
V1rd4	1	NNNNNNNNNNNNN-NNNNNNNNNNNNN-NNNNNNNNNNNNN-NNNNNNNNNNNN-NNNNNNNNNNNN
V1rd6	1	AGAAACTGTCATT-TTTGAGGGGAAAG-CAGGTGAGATTTC-NNNNNNNNNNNN-GAAGGATGTCAG
E17.18294	1	GAAAACTGCAATT-TCTGGAGTAAAGA-GAGATGAGGTCAC-TGAGCTGAAGTT-AAGTGGTGTCAT
V1re1	1	AGACAGAAAAGTT-TCTGGAGGGAAAT-CAGGTGAGATGAA-TGAGATGAAAAT-AAAAGCTGTCAT
V1re2	1	NNNNNNNNNNNNN-NNNNNNNNNNNNN-NNNNNNNNNNNNN-NNNNNNNNNNNN-NNNNNNNNNNNN
V1re3	1	GAAAAAAATAATT-TCTAGAGTGAAAC-TTGGTGAATTAAC-TGAGCTGAAGGT-AAGTGTTGTCAT
V1re4	1	NNNNNNNNNNNNN-NNNNNNNNNNNNN-NNNNNNNNNNNNN-NNNNNNNNNNNN-NNNNNNNNNNNN
V1re5	1	AAAAATTACAATT-TTCTGAGGGAGTT-TTGGTAAATGCTC-TGAGAAGAAAAT-CAGTGCTGTCAT
V1re7	1	NNNNNNNNNNNNN-NNNNNNNNNNNNN-NNNNNNNNNNNNN-NNNNNNNNNNNN-NNNNNNNNNNNN
V1re8	1	AGGTAGTGCAATT-ACTAAGGAAAAGT-CAGGTGAGATTCT-TGATATGAAAAT-AGTTTCTGTCAT
V1rf1	1	TGAAATAATAATT-TCCTGAGCAAAGG-NNNNNNNNNNNNN-TGAAATAATAAT-NNNNNNNNNNNN
V1rf2	1	TGAAATGATAATT-TCTGAAGGGAGAT-GAGATGAGGTCAT-TGAAATGATAAT-AGATGAGGTCAT
V1rf3	1	TGAAATTTTAATT-ACCTGGGGAAAAG-GAGGTGAGGTCAC-TGAGATGATAGC-AGGTGAGGTCAC
V1rf4	1	TGAAATAGTAATT-TCTAAGGGGAGAT-GAGATGAGGTCAC-TGAAATAGTAAT-AGATGAGGTCAC
V1rf5	1	TGAAATAATAATT-CCTGAAGGGAAGG-TAGGAGAGATTAG-TGAAATAATAAT-NNNNNNNNNNNN
E7.8826	1	AAAAATGAAAATT-TTTTAAGGGAAAT-GAGGAGAGGCCAC-AAAAATGAAAAT-CAGTGCTGTCAT
V1re10	1	TGAAATGTAAATT-TCTGGAGGGAGGT-GAGGTGAAGTCAG-TGAAATGTAAAT-CAGTGCTGTCTT
V1re11	1	GGAAACTAAAATA-TCTGGAGGAAGGT-AAGGTGAGGTCAC-TGGAATGGAAAT-CAATGCTGTCAT
V1re13	1	AGAAACTTTAATT-TCTGGAGGGAGGT-GAGGTTAAGTCAC-TGAAATGAAAAT-CAGTGCTGTTAT
V1re9	1	AAAAACGAAAATT-TTTTAAGGGAAAT-GAGGAAAGGTCAC-TAGATTGGAAAT-CAGTGCTGTCAT

```
G7.10220     1 TGAAATTATACTT-ACTTGGGCAAAG-TGGGAAAAGGCAT-AGAACTGAAGAC-AGGTGCTGTCAA
V1rg1        1 AAAAACTAAAGAT-NNNNNNNNNNNNN-GAGGTGAGTGATC-TGAGTTGAAAAT-AGAGGCTGTCAT
V1rg11       1 NNNNNNNNNNNNN-NNNNNNNNNNNNN-NNNNNNNNNNNNN-NNNNNNNNNNNN-NNNNNNNNNNNN
V1rg12       1 NNNNNNNNNNNNN-NNNNNNNNNNNNN-NNNNNNNNNNNNN-NNNNNNNNNNNN-NNNNNNNNNNNN
V1rg2        1 TGACAAAATAGTT-TCTAGGGAAAAGT-TAGGAGAAGCCCC-TGAGCTAAAAAT-AGACGCTGTCAT
V1rg3        1 NNNNNNNNNNNNN-TTTAGGGTAAGGG-GAGGTGAGTGACC-AGAGTTAAAAAT-AGATGCTGTCAT
V1rg4        1 TGGCAGAAAAGTT-TTTTGGGAAAGAA-GAGGTAAGTGACC-TGAGTTGAAAAT-AGATGCTGTCAT
V1rg5        1 AAAAAAAAAACTT-TTTTGGGAAAGAA-GAGGTGAGTGTCC-TGAGTTGAAAAT-NNNNNNNNNNNN
V1rg6        1 TGACAGAAAAGTT-TCCAGGGGCAAAG-GAGGTGAGTGACC-TGACTTGAAAAT-AGAGGTTGTCAT
V1rg7        1 AGAAAAGACAAAT-TTCAGGGGCAAGG-CAGAAAAGTTCCC-TAAATTGATAAT-AGATGCTGTCAT
V1rg8        1 AGAAAATGCAGTT-TCCAGAGGCAAGG-NNNNNNNNNNNNN-TGAGTTGAAAAT-AGATGCTGTCAT
V1rg9        1 AGAAAATGCAGTT-TCCAGAGGCAAGG-NNNNNNNNNNNNN-TGAGTTGAAAAT-AGATGCTGTCAT
V1rl1        1 NNNNNNNNNNNNN-NNNNNNNNNNNNN-NNNNNNNNNNNNN-NNNNNNNNNNNN-NNNNNNNNNNNN
H13.21659    1 AGAAAAAGAAGAT-ACTGGAGATAGAT-CAGGTGAGTACAT-TAAGCTGGAAAT-AGGTGAGTACAT
H13.21773    1 NNNNNNNNNNNNN-NNNNNNNNNNNNN-NNNNNNNNNNNNN-NNNNNNNNNNNN-NNNNNNNNNNNN
H13.22206    1 AGGCAGTACAAAT-TTCTGAGCAAAAC-CAGGTGAGTACAT-NNNNNNNNNNNN-AGGTGAGTACAT
H13.22286    1 NNNNNNNNNNNNN-TTCTGAGCAAAAT-CAGGTGAGTACAT-NNNNNNNNNNNN-AGGTGAGTACAT
H13.22697    1 AGACAGAACAAAT-TTCTGAGCAAAAC-CAGGTGAGTACGT-TAAGGTGGAAAT-NNNNNNNNNNNN
V1rh10       1 AGACAGTACAAAT-TTCTGAGCAAAAC-CAGGTGAGTACAT-TAATCTGGAAAT-AACAGATGTCAT
V1rh11       1 AGAGAGTACAAAT-NNNNNNNNNNNNN-TAGGTGAGTACAT-TAAAGTTAAAAT-AGGTGAGTACAT
V1rh12       1 AGACAGTACAAAT-NNNNNNNNNNNNN-CAGGTGAGTACAT-TAAGCTGGAAAA-AGGTGAGTACAT
V1rh13       1 AGACACTACAAAT-ACCAGAGAGAGAT-CAGGTGAGAACAT-TAAGATAAAGAT-AGGTGAGAACAT
V1rh14       1 AAACAGTACAAAT-NNNNNNNNNNNNN-CAGGTGAGTACAT-TAAGCTGGAAAT-AGGTGAGTACAT
V1rh16       1 AGACAGTACAAAT-TTCTGAGCAAAAC-CAGGTGAGAACAT-TAAGCTGGAGAT-AGGTGAGAACAT
V1rh17       1 AGACAGTACAAAT-TTCAGAGCAAAAC-CAGGTGAGTACAT-TAAGCTGGAAAT-AGGTGAGTACAT
V1rh18       1 AGACAGTACAAAT-TTCTGAGCAAAAC-GAGGTAAGAGCTT-TAAGCTGGAAAT-NNNNNNNNNNNN
V1rh2        1 AGACAGCACAAAT-TTCTGAGCAAAAC-CAGGTGAGTACGT-TAAGGTGGAAAT-NNNNNNNNNNNN
V1rh20       1 TGAAAATGTAATT-ACTTGAGAAAGAG-GAGGAAAAGTCAT-TGACATGGTGAT-NNNNNNNNNNNN
V1rh21       1 TGAAAATGTAATT-TCTTGAGGCAAAC-GAGGGAAAGTCAT-TACAATGAAAAT-NNNNNNNNNNNN
V1rh3        1 AGACACTACAAAT-TTCTGAGCAAAAC-CAGGTAAGTACAC-TAAGCTGGAAAT-NNNNNNNNNNNN
V1rh4        1 AGATAGTATAAAT-TTCTGAGCAAAAC-GAGGTAAGATCTT-TAAGTTGGAAAT-NNNNNNNNNNNN
V1rh5        1 AGACAGTACAAAT-TTCTGAGCAAAAC-CAGGTGAGTACAT-TAAAGTGGAAGT-AGGTGAGTACAT
V1rh6        1 AGACACTACAAAT-TTCTGAGCAAAAC-CAGGTGAGTACAT-TAAGCTTGAAAT-AGGTGAGTACAT
V1rh7        1 AGACACTACAAAT-TTCTGAGCAAAAC-CAGGTGAGTACAT-NNNNNNNNNNNN-AGGTGAGTACAT
V1rh8        1 AGGCAGTACAAAT-ACTGGGGAGAGAG-CAGGTGAGTACAT-TAATCTGGAAAT-AACAGATGTCAT
V1rh9        1 AGGCAGTACAAAT-TTCTGAGCAAAAC-CAGGTGAGTACAT-NNNNNNNNNNNN-AGGTGAGTACAT
I13.21724    1 NNNNNNNNNNNNN-ACTAGGGAGAAGG-CAGGTGAGTGCTG-AGGAATGAAAAC-GAGTGCTGTCCT
V1ri1        1 NNNNNNNNNNNNN-TCCAGAAAGAAAG-CAGGTGAGTGTCC-NNNNNNNNNNNN-NNNNNNNNNNNN
V1ri10       1 NNNNNNNNNNNNN-ACCAGGGAAAAGG-CAGGTGAGTGCTG-AGAGATAAAGAC-GAGTGCTGCCAT
V1ri2        1 NNNNNNNNNNNNN-TCTAGGGAGAATC-TAGGGGAGGTGAC-TGAGCAGAAAAC-NNNNNNNNNNNN
V1ri3        1 AGGCACTGTAATT-NNNNNNNNNNNNN-CAGGTGAGTGCTT-TGAACTGAAGCT-AGGGGAGGTGAT
V1ri4        1 AGGCACTGTAATT-TCCAGGAAGAAAG-CAGGTGAGTGCCC-TAGAATGGAGAT-GAGAGCTGACAT
V1ri5        1 AGGGACTGTAATT-TCCAGGGAGAAAG-CAGGTGAGTGTCC-TAGAATGAAGAT-GAGAGCTGACAT
V1ri6        1 NNNNNNNNNNNNN-NNNNNNNNNNNNN-NNNNNNNNNNNNN-NNNNNNNNNNNN-NNNNNNNNNNNN
V1ri7        1 AAAAACTTTAATT-ACCAGGGAGAAGG-CAGGTGAGTGCTG-TGCAATTAAGAT-GAGTGCTGCCAT
V1ri8        1 AGACACTGTAATT-ACCAGGGAAAAGG-CAGGTGAGTGCTG-NNNNNNNNNNNN-GAGTGCTGCCAT
V1ri9        1 AGACACTGTAATA-ACCAGGGAGAAGG-CAGGTGAGTGCTG-NNNNNNNNNNNN-GAGTGCTGCCAT
I13.11096    1 GGAAAAAGCACAT-ACTTAGGAAAAAG-TAGGAAAAAGCAC-NNNNNNNNNNNN-AATGGCTGTGAT
I13.11171    1 GGAAAAAGCACAT-ACTTAGGAAAAAG-TAGGAAAAAGCAC-NNNNNNNNNNNN-AATGGCTGTGAT
V1rj2        1 AGAAAGAGAAATT-ACTTAGGAAAAAG-TAGGAAAAAGCAC-TCAGATGATAAT-AATGGCTGTGAT
V1rj3        1 GGGAAATGCAAAT-TCTTGGGGAAATG-NNNNNNNNNNNNN-GGAAATGCAAAT-GAGTCCTGTCAT
V1rk1        1 NNNNNNNNNNNNN-ACTTGGGGAAAGG-NNNNNNNNNNNNN-NNNNNNNNNNNN-AATGGCTGTGAT
```
