# Supplementary material for: V1R promoters are well conserved and exhibit common putative regulatory motifs
Source: BMC Genomics. 2007 Jul 25;8:253. doi: 10.1186/1471-2164-8-253 (PMC1955453; doi:10.1186/1471-2164-8-253)
Supplement: Additional file 3 — V1R peak sequences. Text file in fasta format of all putative promoter regions ("peaks") for all V1R sequences used in this study. [file 1471-2164-8-253-S3.doc]

>peak_AB_76

TGTGCCTCTCTTTCTCTAAACTTTCCTATCACTTCTTTTTAAATTTATTTGTAAATTTTACTCACGGCAAGAATCCTGAAGCTGAGCATATGGAAATTAAGGCATACAGAAGCAATTCTACCTGGAATCCTGACATTCTACCTGAGTACTGTTCCAGCAAACGCTGCCAATTAACACTGTAAGGCATAAGCCTCAGAGCTGATGGTAAGTCATGGGAGGCCCAGGAGTTTCTCCTCCCAGAGCAGAAACTATTGCTAAATATGAAATGGTTTGGGATTGATTATACCTCAGGTTCAAAATGAGCAAAGTTAGAGTTGTCTAATTGGTGCAAACTTTCTGATCCCTCCCGAAGGTATTGCAAGATAACTCAGATAAAGTTTGTGCCCAGCCCTCCCCTGATGGCTGTTATCCAGATCACAGGGAGTAAGATCAAAGGCAAGGAAGATGCTCCACTGGAGTGCCAGGTCTCCATGCTCCACAGTGAGAACTTGTCACGTTCTACGGACACTAAAATTACAAATTGCTCACATGCATTAAAGACTCCCTTAATCCTGTGCAGGATCTCCCTAGGCATAGAGTCCTATCCTGCCATGTCTATATTGGTAAGTGTCCTGCACCCAAATGCAGAATACCAGGAAGGCTGAGGTCAGAGGAGTTTCCAGGCTACAAGGAGGTTTTCAGTGTCTACATTGGAGACCCTGAGCTCTGATGGCTCTTCAGCACAGAGGACTATGTTCTGCATGGGTAACAAACATGTTTTCTTAGGATATCCATTTTCTGTAACTCCTAAAATTCATCAATTTTGCACACCTCTGGAATATCCATAGATTTTTGGTTTGCATTCATTTTT

>peak_AB_77

TTCAGGTGCCCATGACTGCCTCACCAAAGACCTCTAGCTCTGCCTCTCTCCCTCTACACTTTCCTATCATTTCTTTTTAAATTTAATTGTAAATGTCACTCACAGCTGCAATCCTGAAGCTGAGCATATAGAAATTAAGACACATAGAACCAATATTACATGGAATTTAGATACTTCATCTGAGGGTTGACCCTGCAAAAGGTTGCCTGTTAACATTGAAGGACAAGAGCCTCAGAGCCAATGGGAAGTCATGGGAGGCAGAAGAGTCTCTCACCCCAGAGCAGACACATCACAAAGTATGACATGTTTTGGGATTGATTATACCTGAGTTGTCTAATTGGTGCAAACTTTCTGATCCTTCCCACAGGTATTGCAAGATAATTCAGATAAAGTTTGTGCCTAGCCCTCCCCAGATAGTTGTTATCCAGATCACACAGAATAAGATCATAGCCAGGGAAGATGCTCCAGTGGGTTGCCAGGTCTCCATGCTCCACAATGAGAACTTTTCATGTTCTACAGACACTAAAATTACAAATTGCTCACATGCATTAAAGACTCCCTTAATCCTGTTCAGGATCTCCTCTGGCACAGAGTCGTCTTGTCATGTCTATCTGGGTAAGTGTCTTGCACCCAAGTGTGGAATCACAAGGTGGTATGGGCCCAGGCAGGCTGATGGTGGAAGAATCCTCAGGCTACAGCAAGGTTTTCAGCATCTGCAATGGAAAAGCTGAGCTCTGACATCGCCTCAGCACAGAGGACTCTATTCTGCATAGGTAATGAACGTGTTTTCTTCTTAGGATATTCATTTTTTGTAACTCCTTAAATCCATCATTTTTGCACACTTCTGGAATATCCATAGATTTTTTGGTTTGCATTCATTTTTAAAAAAGTAAATAACTCTCTGTCTCAGCACTTAAACCACACAGTGAATTTCTTGACTTATTCTTTCATACAGATCT

>peak_AB_78

TCATTTCTACATTTAATTTAAGATCATTTTTAGTTTTTACTAAGAGAAGAAAATCTGGAAGTCATAGCACACAGAACAAAAATTTCTTGGAAACAGGTCCTCCATGTGTTGAAGGACCCTGGAAGAAGCAGCCTGCCACTCACTGTGTAGATTTCATCAGAAACATGGAAGCTCAAGGAAGGCACCAGGAGTCTCACTGCTAGCAGATGAATTGTGCAGAAGTTAAGTTTATATTCATTTTGCCTGAGAAAAAAATTAGCAAGTTTGTGTTGACTACATTGTGTCTTGTTCTAACTTAGAGGTGTATCAAGGGAGCTCATTGAAAAGTTTGGGCCCAGCTCACACAGGACAGATGTTACCCAACAGAGTCCAGTCAACTCAACAGGTAAAATGTTCCAGTGGGATCCCACATGTCCCTGCTCTCCAGTGGGAACTTGTCACTGACAGTCACTAAAATTGCAGGCTGCAGAGATGCTTTAAAGACTCCCTGTACCTAAGCCTGTGCAGGATCTCCTCAGCTCAGACTCCACCTGCCCCACCCCTTCCATCTGAGTAAGTGCCCTGAGTAAGCACAGAATCAGAAGGTGGCTGGGTCCCAGGCAGGCTGTGGGAGTTGCAGGGGTAGTTTAAGTTTTTGCCATGGGGATGCTGAGTCTGAAGGATGCTCAGCACAGGGTACTGTATGCTGCACCAAGTGCATTTTCATCCTGGGGTATTCAATTT

>peak_AB_79

TCTCCCTTCATTTCTATATTTAATTTAAGATAACTTTTACTTTTTACTAAGAGAAGGAAATCTGGAAGGCATAATACACAGGACAAAAATTTCTTGGAAACAGGTCCTACATGTGTTGAAGGATGCTGGAAGAAGCATACTGTCACTCACTGTGTAGATTTAATCAGAAACATGGAAGCTCAAGGAAAGCACCAGGAATCTCACTGTTAGCAGATGAATTGTGCAGTAGAAGTTAAGTTTGTATTCATTTTGCTCGAGGAGAAAATTAGCAAGTTTTTGTTGACTACATTGTGGCTTGTTCTAACTTGCACGTTTGCCGAGGAAGCTCAGTGAAAAGTTTGGGCAGAGCTCACACAGGACAGATGTTACCCAACATCACAGAGTCAACTCAACAGGTAAAATGTTCCAGTGGGATCCCACATGTCCCTGCTCTCCAGTGGGAACTTGTCACTGACAGTCACTAAAATTGCAGGCTGCAGAGATGCTTTAAAGGCTCCCTTCACCTAAGTCTGTGCAGGATCTCCTCAGCTCAGACTCCACCTGCCCCACCCCTTCCATCTGAGTAAGTGCCCTGAGTAAGCACAGAATCAGAAGGTGGCTGGGTCCCAGGCAGGCTGTGGGAGCTGCAGGGGGAGTTTCAGATTCTGCCATGGGGATGCTGAGTCTGAAGGCCCTCGGCCGAGGGTACTGTGTGCTGTATCAAGTGCATTTTCTTCCTGAGGTATTCATTTACTCAAGGTCGTAAACTTACCTAATTTTTATAAGATGTCTGTTAATTTTAATTTCAATTTATTTTTCTGAGCTAATTACTAAATTTACTT

>peak_AB_80

GTGCCTCTCTCCCTCTTCAGTTTCCTATAATTTCCTTTTAAATTTACTTGTAAGTTTCACTCAGAGCTTGAATCCTGAAGCTGAACATATGGAAATTAAGGCTCATAGAACCAATACTACACGGAATCCAGATATTCCACATGGGGACTGACCCAGCAGAAGGCTGCCTGTTAAGACTGCAGGGCGAAAGCCTCAGAGCTGATGGGAAGTCATGGAAGGCCCAGGAGTCTCTCACCCAAGAGCAGACACATCACAAAGTATGGAATGGTTTGGGATTCATTATACCTGAGCTTCAAAATGAGCAAAGCTAGAGTTTAGTTTGTGCGGATTTTCTGATCCTTCCTACATGTATTACAAGTTAACTCAGACAAAGTTTGTGCCCAGTCCTCCCCAGGTGGCTGTAATTCAGGTCACAGGGATTAAGATCAAAGGAAAGGAAGATGCTCCACTGGGATGCCATGTCTCCCTGCTCTCCAGTGAGAACTTGTCACATTCTACAGAAACTAAAATTACAACTTGCTGAGCTGACATGCATTAAAAACTCCCTTAATCCAAGCCTGTGTAAGATCTCCTCATGAAGAGTCCATGTCTATCTGGGTAAGTGTCCTGCACTCAAGTGCAGAATCACAAGTGGTTTGGTCCCAGGTAGGCTGAGAGAGAAATTGTTCCTGAGCTATAGGGAAGTTTTCAGCATCTGCACTGGAGAAGCTGAGCTATGACATCCTTTCAGTACAGGGGACTGTGTTCTGCATGGGTAATGAATGTGTTTTCTTCTAAGGATATTCATTTTCTGCAACTCCTAAAATTCATCAGTTTTGCACAGTTCCATAGAGTTTTGCTTTG

>peak_AB_81

ACTTTACTTCCATATTTTCTTTAAATTCATTTTTACAGTTTACTAAGAGAAGGAAATCTGGAAGTCATAGGTCACAGAATCAAGATTTCTTGAATACAGGTTCTCCATGTCTTGAAGGAACCTGGATCAAGCATCCCACCACTCACTGTGTAGATTTAATGAGAAACATGGAAGTTCAAGGAAGGCACCAGGAGTAACACAGTTACGCAGATGAATTATGCAGTAGAAGTTTAGTTTGTACTCATTTTGCCTGAGGAGAAAATTAGCAAGTTAGTGTTGACTCCGTTGTGTTCTGATCTAACTTGCAGGTGTGCCAAGGATGCTCAGTGAAAAGTTTGGGCCCAGCTCACACAGGACAGATGTTACCCAACAGAGTAGAGTCAACTCAACAGGCAAAATGTTCCAGTGGGATCCCACATGTCCCTGCTCTCCAGTGGGAACTTGTCACTGACAGTCACTAAAATTGCAGGCTGCAGAGATGCTTTAAAGGCTCCCTTCACCTAAGCCTGTGCAGGATCTCCTCAGCTCAGACTCCACCTGCCCCACCCCTTCCATCTGAGTAAGTGCCCTGAGTAAGCACAGAACCAGAAGGTGGCTGGGTCCCAGGCAGGCTGTGGGAGGAGGAGCTCTGGAGCTGCAGGGGGAGTTTCAGCCTCTGCCATGGGGAAGCTTAGTTTGAAGGACCTCAGTACAAGGTACTGTATCCTGCATCAAGTGCATTTTCTTCATGGAATATTCATTTTCTTAAGGTCCTAAACTTACCCATGCTCAGCCtaattttataagatatcaattaattttaatttcaatttatttttC

>peak_AB_82

ATTTCTTTTTAAATTTACTTGTAAATGTCACTTGCAGCTGGAATCCTGAAGCTGGGCATATGGAAATTAAAGTACACAGAACTAATACTTCATGGAATTCAGATATTCCACATGAGGACTGAGCTAGCCAAATGCTGCCAATTATCACTGTAGGGCATGAGTCTCAGAGCTCATGGAAAGTCATGGGAGGCCCAGGAGTCTTTCACCCCATAATAGTTTCACATCACAAAATATGAAATGGTTTAATATTGATTACACCCGAGGTACAAAATGAGCAAAGTTAGAGTTGTCTACTTGGTGCAAACTTTCTGCTCTCTCTCGAGGGTATTGCAAGATAACTCAGATAAAGTTTGTGCCAAGCCTTCCCCAGATGGCTGTCATTCAGATCACAGGGAGGAAGATCAAAGGTGAGGAAGATGCTCCACTGGGATGTCAGGTCTCCCAGCTCTCCAGTGAAAACTTTCACCTTCTACAGACAGTAAAATTACAAATAGCTGATGCACATTAAAAAACTCCCTTAATCTAAGCCTACATAGGCTCCCCTCAGGCACAGAGTCCTTTCCTGCCATGTCCATCTGGGTAAGTTCTCTGTACCCAAGTGCCACAGAATCACAAGGTGGTTCAGTCCTAGGCAGACTGACTTCGAAGTGGTTCCAGGGCTACCGGGTGGTTTTCAGCATCTGCAATGGAGAAACTTAGCTCTGACATCCCTTCAGCACAGGGGACTCTGTTCTGCATGGGTAATGAATGAGTTTTCTTCTGAGCATATTCTTTTTCTGTAACTCACAAAATTCTTCAGTTTTGCACACTTCTGGCAAATCCA

>peak_AB_83

TTCCTATCTTTTCTCTTTAAATTTACTTGTAAATGTCACTCAAAGCTTGAATCTTAAATCTGAACATACGGTAATTAAGGCACACAGAACTAATACTACATAGAATCCAGATGATCCACTTGAGGACTGATCCAGCCAAGGCTGCCTGCTAACATTGGAGGTCAGGAGACTCAGAGCAGAAGGGAAGTTATGGGAGACCCAGGGGTATCTCACCCCAGAGCAGACACATCACAAAGTATGAAACCGTTTGGGACTGATTATACCTGAGGTTCAAAATGAGCAAAGTTAGCGTTATGTAATTGGTGCAACTTTTTGATCCCTCCCGTGGGTATTGTAAGATAACTCAAATAAAGTTTGTTCCTAATCCTCCCGGATTGCTGTTATACACATCACATGTGGAAGAGCTTACATTAGAGTAAGATCAAAGGCAGGAAAGATGCTCTACTGGGATGCCAGGTCTCCATGCTCTACAGAGAGAACTTGTCATGTTTTAAAGACACTAAAATAACAAATTGCTCACATGCATTAAAGACTCCCTTAATTCTGTGCAGGATCTCCTCAGGCACAAAGTACTCCCTGCCAAGTCCATCTGGGTAAGTGTCCTGCACCCAAGTGCAGAATCAGAAGGTAGTCTGGTGCCAGACAGGCTGAGGGAGGATGAGTTCTAGGTCTACAAGGAGGTTTGCATCATCTGCAATGGAGACACTGAGCTCTGATGTCTCTTCAGCACAGGGGATTGTGATCTGCATGGGTAAAGAATGTGTTTTCTTCTTAAGATATTCATTTTCTGTAACTCCTAAAATTCATCAGTGTTGCACACTTCTGGAATATCCATAGATTTTTGGTTTGCCTTAATTTTTCTAAAGTAA

>peak_AB_84

TTAAATTTACTTGTAAATGTCACTCAAAGCTTGAATCTTAAATCTGAACACATGGAAATTAAGGCACACAGAAGCAGTACTATATGGAATCCTGATTTTCTGAGAACTGACCCAGGAAAGGCTGCCAGCTAACACCATAGGATGAAAGCCTCCGTGGTAAGTCATGGGAAGCCCAGAGTCTCTCACCCCAGAGTAGACACATCACAAAATATGAAATCATTTAGGACTAATTATACCTGAGATTTAAAATGAGTAAATTTAGCATTATATAATTGGTGCAACTTTCTGATCCTTCCCACAGGTATTGCAAGATATCTCAGATAAAGTTTCTGCCCAGTCCTCCCCAGATGGTTGCTATCCACAACACAGGGGGACAGATCAAAGGTGAGGAAGACGCTCCATTGTGATGCCAGGTCTTCATGCTCCACAGAGAGAACTTGTCATGTTCTACAAACACTAAAATAACAAATTGCTCACATGCATTAAAGACTCCCTTAATTCTGTGCAGGATCTCTTCAGGCACAAAGTCCTCCCCTGCCAAGCCCATCTGGGTAAGTGTCCTGCACCCAAGTGCAGAATCAGAAGGTAGTCTGGTGCCAGACAGGCTGAGGGAGGATGAGTTCTCGGGCTACAAGGAGGTTTTCAGCATCTGCAATGGAGACACTGAGCTATGATGGCTCTTCAGTACAGGGGATTGTGATCTGCATGGGTAAAAAAATGTGTTTTCTTCTTAAGATATTCATTTTCTGTAACTTCTACAATTCATCAATGTTGCACAGTTCTGGAATATCCATAGATTTTTGGTTTGAGTTCATTTTTCTAAAGTAACT

>peak_AB_85

CTAAGGTCTGGAAAGTTGGAAGTCACGGGGTACAGAAACACGATTTCTTGGATGCAGGTTCTCTCTGTGTTGAAAGATCCTGGAAGACACAGCCTGCCACTTAATATAGATTTAATCAGAAACATGGAAGCTCAAGGAAGGCACCAGGAGTAGCACAGTTACGCAGATGAATTATGCAGTAGAAGTTTAGTTTGTAGTCATTTTGCCTGAGGAGAAAATTAGCAAGTTTGTATTGACTCCATTGTGTTCTGATCTAACTTGCAGGTGTGCCAAGGATGCTCAGTGAAAAGTTTGGGCCCAGCTCACACAGGACAGATGTTACCCAACAGAGTAGAGTCAACTCAACAGGTAACATGTTCCAGTGGGATCCCACATGTCCCTGCTCTCCAGTGGGAACTTGTCACTGACAGTCACTAAAATTGCAGGCTGCAGAGATGCTTTAAAGGCTCCCTTCACCTAAGCCTGTGCAGGATCTCCTCAGCTCAGACTCCACCTGCCCCACCCCTTCCATCTGAGTAAGTGCCCTGAGTAAGCACAGAATCAGAAGGTGGCTGGGACCCAGGCAGGCTGTGGGAGGAGGAGGAGCTCTGGAGCTGCAGGGAGAGTTTCAGCCTCTGCCATGGGGAAGCTGAGTTTGAAGGTCCTCAGCACAGGGTACTGTATGCTGCATCAAGTGCATTTTCTTCATGGAATATTCATTTTATTAAGGTCCtaaaattacttaaattttatctcatttaatttttatttcaatttatttttCTGATCTATGTACCAAATTTACTTTGTCATCCATAA

>peak_AB_86

GGAATATAAGAAAACACAATCAACACTGCCTTGAAAACAGAGAAGATCAACCCAGGGAAAGGCTGCCTGACAAAACTTGTATAGAGAGGACCCTCAGGTGCACTGGGAAATTATGTGGAGACAAGGAGTCCTGCATCCCATTAGAGAGCCATCACAGTGAAGGAAACTACTCAGAATTAAGTCTAAGGAGTAAAACAAGAAAAGTCAGGTTGTGTATTTTGTTGCAAACTTTCGGATGTGGCAGATGTGGCAAGGGAGCTCAGATAAAGTTTTTGCTCACCCTAACATGATAGCTATTATCGAGGACAGGGTGACATAAAGGTGGGAATGATGCTCCACACTGGGATTTTTATGCTCTTCACCTGGAACTTGTCACCTTAGAAAGACACTAAACTTACAAGCTGTAAGGATGCATTAAAGACTCCCTTCACCTAAGCCTGTGCAGGATCTCCTTGGGCAGAGTTCTCCCCTGCTATGTCCATCTGGGTAAGTGTTTTGCACCAGGGGCAGAATCAGAAGGTAGTCAGGATCCAGGCAGGCTGAAGGAGAAGGAGCTCAGGGGCTGAAGGGAGGTTTACAGCATCTGCAGAGGAAACCTCCGAGCTCTGATGGCCCCTCAGTACAGGGCACTGTGTTTTGCACAGCAACAAGGGTGTTTTCTTTTCCAGATATTTATTTCTCTGATATAAAATCTCTTAATATTCTATTTAGACTTTCCTTCTTTTTTAAAAAAACTAATGATCCACATTA

>peak_AB_87

TTAAGGAAACTACTCAGAATTAAGTCTAAGGAGTAAAACAAGAAAAGTCGGGTTGTGTATTTTGTTGCAAACTTTCTGATGTGGCAGATGTGGCAAGGGAGCTCAGATAAAGTTTTTGCTCACCCTCACAAGATAGCTGTTATCGAGGACAGGGTGACAGAAAGGTAGGAATGATGCTCCACACTGGGATTTTTATGCTCTTCACCTGGAACTTGTCACCTTAGAAAGACACTAAACTTACAAGCTGTAAGGATGCATTAAAGACTCCCTTCACCTAAGCCTGTGCAGGATCTCCTTGGGCAGAGTTCTCCCCTGCTATGTCCATCTGGGTAAGTGTTTTGCACCAGGGGCAGAATCAGAAGGTAGTCAGGATCCAGGCAGGCTGAAGGAGAAGGAGCTCAGGGGCTGAAGGGAGGTTTACAGCATCTGCAGAGGAGACCTCCGAGCTCTGATGGTCCCTCAGTACAGGGGAATATGTTTTGCACAGCAACAAGGGTGTTTTCTTTTCCAGATATTTATTTCTCTGATATAAAATCTCTTAAAATTC

>peak_AB_89

ACCAACACTAAATGGAAATCTGTTGCTCAATGGGAGGGCTGGCCCAGGGAGAGGCTGTCAGTCAACACTGTTGGAAGAGGATCCTCAGAGCCCTGAGAAGTAATGGGAGAGGCACAGGAGTCACACATCTTAAAGAGAGGCATTAGGAAGAATAAAATTGGTCCCAATTAAGTCTAAGGAGTAAAGTGGGGAAAGTTAGTGTTGTCTATATGACATAAACTTTCTGATCCCTCCCACAGGTGTGGTAAGGCAACTCAAAGTTGACCTCAGCCCTCTACAGATGGCTGTCATCCAGGACAGTGAGAGTGAGAACAATAGGGAGGTAGGATGCTCCACTGGGATGCCATGCTTCAGTGCTCCCCAGCAGGAGCTCGTCATCCTCAACAGACATGAAAGCTGAAACCTGTAGAGAGTTAAAGACTCCTTTCACCTAAGCAGGATCTCCTCAGTCACAGAGACCCCGGTTCCACGTCCACCTGGGTAAGTGCCCTGCACCCAAGTGCAGAATCAGGGGGTGGTCAGGTCCCAGGCAGGATGAGGTTTTCAGGATCTATAGAGGAAATACTGAGATATGTTGGCCCCCCACCATAGGGGACTATGTTCTACATGGGTAAAGTGCATGTTTTCTTTTTAGGGTATTTCCTGAAACCTCTAAAACTTAGTAAACTTCCCATTGGAATCATCAAAGTTTTATATTTACATTTTGTTTTAA

>peak_C_48

gTATGCTCTGTAAGAAAAATCATTTACCCTCAGTGTGTTTGGAGAAGGTGACATTTTCTTAATTTTCTCAAAGGAGCTGTTACTTTCTAGTTGAGATCCTAAAGCCACTGTCAAGTATGGCAGACACCATGAATGCTTTGGAAGCAGGACGGATATCTTGGGGAATCTAGATTGTTTTTCTATTTCTTTTAAATGAGACATCATTACTTTTACTTTTTTTTTCCCACAAGGACTGGGATCAGTTAATCCACACAGACAGGTGTGGCCTGGAGAGAATAGCTCACACACGGGAAACCCAGAGTCAGCTTTTCCCACTCTACTGTTCTCCTGGAAGTAGGAAAGTTTACTTTTCTATTTGATGATGCCACTTTGTAACAACTTCAATGTCAGAACTGCCTTTTCCAGATGACAAGATAACTGTAGGTCTAGAGGGAGATAAGCTGAGGACAGTTCTTCTGTATAACTACTTCTTAGGATCTCATTCTACAAGGATGTTTATGGGAGGTCCAGTCGGTGAGTGGTTCTTCTTCTTTTTAAAATTCTCTTCATTATAGTGAGAGCAGTAGGTGGTAGAATGAAGCAGGATTCTGTAATAAATGATTAGAGCATTTTCTACTTACAATTTTACTTCTGAGTTATATTGTATCATAAGTAAACTATAGATGCTTGCAGACTTAAGAATGTTGGTGTAATGATTAATACAAGAAAACAGATTTCACTGTCTGATCTCTTCTACATGTGAGAGTATAGAAAGCCTAGAAGAAGGATGGATAGAGACAGGAAAAGCTAATAAGCTGAAG

>peak_C_49

CTATATTTTACAGTCCCGCAGCCTGATTTCTTCTGAAAATAACTGTTATACTATCTCCTGTGTGTGTGCTCTCTATTAAACATCACTTAACCTTAGTGTGTTTGAATGAGATGACACTTTTCTAATTTTCTCTAAAGAGCTGTTAATTTCTAGCTGTGACACTGCAGTCACTGTCATATACTTATAGAGGGCACCAGGAATGCTTTGGAAGCAGGATAGATATCTTGGGGGAATATAAATTGATATTCTACTTCTTTTAAATGGGATATTATTATTCTTACTTTTGTTTTGTTTACAAGGTTTGGGATCAGTTAATTTACATACACAGATATGGCCAAGAGAGAAATCTCACACATGGGAAACCCAGAGCTCAGCTGTTCCTATACTACTGTTCTCCTGGAATTAGGGAAGTTTACTTTTCCATTTGATGTTTACAGTTTATGACAATGTTAAGGCCAGAAGTGCCTTCTCCAGATGAAGTGATAACTATGGGTCTGGAGGGAGATAACCTGATGACATTTCTCCCATATAACTACTTCTGAGGGCCAACTTCTTCAAGGATGTTCTGGGAAGCCCAGATGGTGAGTGGTTTTGTTTTGTTTTCTCTTTATTATAGTGAGACAGGAGGTGGTAGAATGAAAGAGGATTCTTTAATAAATGAGTAGAGAATTTTCTGCTTACAATTTTACTTTTGAGCTATATTGTATCATAAGTAAACCAGAGATGCTTGCAGATATGAATACCAGTGTATTGATTTATAAAAGAACACTGATTACACTCAGTTTGGTCTCTACTATATGTGACAGTATAGAGGGCCTAGCAGGATGGATAGAGACAGGAAAAGCTAACAGGCTGCAGATGATCATTGAGTATTATATAGTAAATGAAAATGTCACTACACTCACTACTTTTTATAATGAATATATGCTGGCATATATTTAAAAATTAACTTTATCTCAGATCAATCAGACAAAAGATTGTA

>peak_C_50

CCCACAACCCGATTTCTACTGAAAATACAGTCATATTACCCCATGTTTGTTCTCTGTAAGAAAAATCCTTTACCCTTAGTGTCTTTGGAGGGGGTGACATTTTTCTAATTTTCTCAAAAGAGCTGTTAATTTCTAGGTGAGACACTGAATGCACTGCAATGAAAGTATGGAGAGTATTATGAATGCTTTTGAAACAGGAAGGATGCCTTGGGGAATCTAAATTGTTTTTATACTTCTTTTAATTTGGATATTATTATTCTTCCTTTTTTGTTCACAATTACTGGGAACAGTTAATCCACATACTCAGTTATTGCCAGGAGAGAATATCTCACACTTGGGAAACACAAGGCTCAGCTGTATCTACTCTACTGTTCTCCTGGAGTTAGGGAAGTTCACTTTTCCTTTTGATGGTCCCGCTTTGTAACAATGTCAAGGTAAAAAAAAAATGCCTTCTCCAGATGACACGATAACTGTGGGTCTAGAGGGAGATAAGCTGATGGCATTTTTCCATATAACTACTTCTTAGGATCTCCTTCTACAAGGATGCTTATGGGCAGTGCAGACAGTGAGTTTTTTTTTTTTTTAAATTCTCTTCATTATAGTGAGAGCAGTAGGTGGCAGAATGAAGTAGGAGTCTTTAACAAATGATTAGAGAATTTTCTGCTTACAATTTTACTTTTGAGTTATGTTGTATTTTAAATAAACCAGCGATAAGAATGTTAGTGTAATGATTAATAAAAATACAGATTTAAGTCAGTTTGATCTCTTCTATACAACAGTAGACAAGGTGTAGCAAATGGGTGGATAGAGACAGGAAAAGCTAATAAGCTGAAGATTGACCACTGAGCATTATATATTAAATGGAAATATA

>peak_C_51

TTTCTTTTCTGTGTGCTTTGTAAGAAAAATCATTTACCCTAAGTATGTTTGGAGGAGGTGACATTTTCTTAATTCCTGAAAAAGGGATGTTAATTTCTATGGAAGATCTCAAGTATGTAAGACACCATGAAAGCTTTGGATGTGGGTCAGATACCTTGGGAATGTAAATGTTTTCCTGCTTCTTTTAAATGGAATATTATTTTTGTTACTTTTGTTTTGTTCCCAAGAACTGGGATCAGTTAATCTACACAGATCTGGCCTAGAGAGAATATCTTACACACGGGAGACCCAGAGATCATTTGTTCCTACTCCACTCTTCTGAAATTAGGAAAGTTCACTTTTCCATTTGATGATACAACTTTGTAACAACCTCACGGTCATAACTGCCTTCTCCATATGACATGAACACTATGGGTCTAGAGGGAGATAACATGATGACATTTGTTCCTTATAACTACTTCTTAGGACCTCATTCCACATGAATGTTCATCAAAGGCCCAGCTGGGTGAGTGTTTCCTTTTCAGTTCAGTATAATGAGAGCTGTAAGAAGGTGGTAGAATGAAGCTGGATTCTATAATAAATGAGTAGAGAATTATCTGCTTAGAATTTTACTTTTTAAGCTAAATTGTATTATAAGCAAACATAAGACATACTTGTAGACATATAATATCTGTATAACAATTAATAACAGAACATATATTTTACTCATTTTGATCTTTTATTATACATGTGACAGTAGAGGTGGATATTTGGAAGAGGACAGGGTCTAATAGAAGGAGGGAAAAAGAAAAGGCTCATAAGATGAAGATGATTAATATGTATTATATTTTAAATG

>peak_C_52

CCAATTTCTTGTAATAACACATTTACGTTACCTCACCTCTGTGTGCACTCTAAGAAAGATTGCTTTCCTCAAATGTGTTTGAATGGGCAGCCATTTTCTTCATTTTAGCAAAAATGCTGTTAATTTATGGGTGAGACCTTCAATCCACTGTGAAGTATGTAAGGAGGCTACCACAACACAAATGTTTTGGAATCAGAACAGATACCCTAAGGAATCAAAAAGGTTTTGCTACTTCTTTTAAGTGTGATACTATTATTGCTATTTTTGTTTTGTTCACAAAGACTGGGGTCACTTAATCCACCCAGACAGTTATAGCCTGGAGAGACTATCTCACACACGGAAACCCAGAGCTCACCTGTCACTAAGCCATTGTTCCCCTCAAATTAGAGAAGTTTACTTTTCCATTTGATGGAATCACTTTGTGACAACCACAAGGTCAGAACTGACTTCTCTGGATGCCATGATAACTACTGGTCTAAAGGGAGAAGAGCTGATGACACTTCTTCCTTATAACTGCTTCTTAGGGCCTCATTCTACATGGATGTTTTTCAAAGGCCCTGGTGAGTGAGTGTTTCTTTTTGTCTTCATTAATAGTGAGAGCTGAAAGAAGATAGTAGAATGAAGCAGGATTTTCTGTCATAAATGATTAGAGAATTCTCTGCTTATAATTTTACATTTGAACCATACTATATCATAAACAAGTACAACAGATTCTGCAGACAAAAATATTAGTGtaataatttataaaataatataGATTTCATTTACTACAATATCAAGAATATATATATATATAAGAAGGAGCTGGAAAAGCATAGGTGC

>peak_C_53

AGCCATGCCCACTATCAACTGATTACATCTCTGTCCCCTTAGCCCCATTTCAGCCCCATAACATTTATATTACCACACTTCTGTGTTTAATACAAGAAAGATATTTTTCCTCAAGTGTGTTTGGAGGAGGTGAACTTTTCTTAATTTTATCAGAATCTCTGCTATTATCTAAATGAGACCCTGAAACCAATGTCAATTAAGCATGGAGACCATGAGGAAAGTTTGGGAAGCAAAGCAGATACCTTGGGTAATCTAAATTGTTATTCAACTTCTTTTAAATAGGGTATTATTACTTTTACTTTTGTTTTGTTCACAAGGACTGGGATCAGTTAATCCACATAGAGAGATATAGCCCGGGGAGAATACCATACCCAATAGAAACCCAGAGCTCTGTTGTGCATACTCCACTTTTCTGAAATTAGGGAAGTTCACTTTTCCATTTAATGGTACCTCTTTGTAACAACCTCAAGGTCAGAAATGCCTTCTCCAGATGACATGATGACTGTGGGTCTAGAGGGAGACAAACTGATAACATTTCTTCCTTATAACTACATCTTAGGATCTCCTCCTACAAGGATGTTTATGGAAGGCTCAACCGGGTGAGTATTCCTTTTTCTCTTCATCATATTGAGAGCTATAAGTATGTGTTAGAATAGAGCAAGATTTTCTTTATTAAGTGCTTAGATAATTCTCTTGCTTAGAATTCTGCTTTTGAGCCAAGTGTTTCATGAGCAAACACAGCACATCCTTGTAGACATAGAACTACCAATATAATGATTTTTGAAATAACATATATTTTGATTAATATGATCTCTGATTGTGACACTAAAAGTGAGctattc

>peak_C_54

aacttgaaggtttgacatgtttcatggtgtcttacatttATAATACTTTCTAGGTATGGTCTCTTATCAACTGAGCTACATCCCGATAAACAGTCCTGTTTCTCCTGATAAAAGAATTTCATTACCTCACTTCTGTGTGCTGTAAAGAACGATTACTTTTCCTAAGTGTATTTGGAGGAGGTGACTTCTTCCTAATTTTATTAAAAGTGCTGTTAATTTCTAGATGAAACCCTGAAACCTCTGTCAAGTAAGTACGGAGGGGACCCATGGAGGTTTGGGAAGCAGGAAGGACACCATGAGAAATCTAAAATGTTTTCCTACTTCTTTTAATGGGGGCATCATTATTATTGTTATGCTTGTTTTGTATAGGACTAGGATCGGTTGATCCACATAGACACATATGGCCCAGAGAGAATAGCTGACACATGGGAAACCCAGAGTTCAGCTGTTCCTAATTCACTGTTCTCCTGGAATTAGGAAAGTTTACTTTTCTCTTTGAAGGTACCACTTTGTGACAATGTCAAGGTCAGAATTTCCTTCTCCAGATGACATGCTAATTAAGGGTCTATAGAGAGATGAGCTGATGACATTATTTCCTTATAACTAGTTTTGGGGCCTCATGCTACACAAATGCTTGTCAAAGGCACAACCTGGTGAGTGTTTCTTTTCCTCTTCATTATAGTGAGCTTTAAGTTAGTGTTAGAATGAAGCAGAATTTTCTCTACAAAGTGATTAAAAAATCCATTGCCTGGAATATTATTTTTTGAGGCCTAGTATGTAATAAGCATTCAACAGATGCTTATAGACATAAAGGTACTAGTGTAATAATTTATAAAAGAACATAGATTTCATTTCTTTTGATCTCTGATTATGTACATGACAGAAGGAAGCTATTGGAAGGAGGGCAGGTTCTTAGTAGAATGATGGAGAGACAGGAAAGGTTAATAAGGTGAAGGTGATCAATGGGTATTATGGGTGAATATGTCATGAAGCCCATCACGTTTTACAATAGGGATCCATTCATGTGCATGTTTAAAAGTAGCTCCTAGTGCACCATTTTAAGCAGCACATATAACATAGGATTCTCCTGAGAGTGTACTCTAATAATTCTAAGGTCATGTCTCCACCTAGATCTTGCCAGCACTGTTAGTTTTCCACCCCTGTAACAAGACACTGAAATACTTAAGAGTGTGAAAAACAAAGCACACAGGTATGTTGGTGTGCACAAGTGCACTTATACTATCAGGTGACATTCAAGTTGTAATGTGTATTTTCTACTTTTCTTTAGTTAACCTGATGTATGTGTTTAATTATATAAAGTGTAAGGTAGGAATATACAAGAAATATTAACTAATTTAGAGTTTATCAGTAATTTTCTGGTTTGTTAAGTTTGCATATGCTTAGTAATTCTTAGGAAAGTTT

>peak_C_55

aagaaaGAATACTTTCCTCAAGTGTGTTTGGACAGGGTGAACTTTTCTTAATTTTATCAAAGGCACTGTTAATTTCTAAATGAGACACTGAAGCCACTGTCAATTTAGCATAGAGACCGTCTCACAAGTTTAGGAAGCGGGACAAATACTTTGGGGAATCTAAAGTGTTATTCAACTTCTTTTAAACAGTGTATTATTCTAACTTTTGTTTTGCTCTCAGGACTGAGATCAGTTAATCCACATAGAGAGATATGGCCTGGGGAGAATACCACACCCAATTGAAACCCAGAGCTCAGCTGTGTTTACTCCATTCTTCTCCTGGAATTGGGGAAGTTCACTTTTCCATTTGATGCTGCCACTTTGTTAGGACCCTAAACTCAGAAGTGCCTTCTCCAGATGACATGATAACTATGGATCTAGAGGGAGACAAGCTGATGACATTTCTTCCTTATAACTACGTCTTAGGGTCCCATACTACAAGGATGTGTATGGAAGGCTCAGCAGGGTGAGTATTTTTTTTCTCTTCATTATAGTGAGAGATATACATATGTTTTAGAATGAAGCAGGCTTTTCTCTATTAAGTGATTATAGAATTCTCTTGCTCAGAATTTTACTTTTGAGCCAACTGTATCATGAGAAAACACAACAGGTCCTTGTAAGCACAAAAATATGAGCATAAAGATTTTAAAAATACTTTATATTTTGATTAATATGATCTCTGATTATTCATGTGTTACTAAAAGTGAGCTATTGGAAAGAAGACGCGGCCCAGCAGATGGATGGAGAGAGTGGAAGGAAAGTCTCAGAAGGTGAATATCATCTATGGGTATTACAT

>peak_C_56

cccacatACATATACTTTCTAGGCTTCGACTTTGTCATTGAGCAACATCCCAGCCACATGTCACAGTTTCTCCTGATAATACATTTACATTACCTCACTTCTTTGTGCTCTGTAAAAAAGATTACTTTTTCTAAGTGTGTTTGGAGGATTGACATCCCATTTTTTTTAAAATGAGAGTTGCTAATTTCTAGGTGAGACACCAAAGCCACTGTTAAGTAAGTGTGGAGGGAACCATGAATGTTTTGGAAGTAGGAAGGATACCTTGGGGAATCTAAAATGTTTTCTGCTTCTAAGAGAGATATTACTATTGTTACTTTTGTTGTGTTCCCATGGACTTGGATCAGTTAATCCACAAAGACTCATATGACCTGGAGAGAATAATTTACACATGGGAAATATGGAGCTCAGCTGTTCCTACTCTACTGTTTCCTTGGAACTAGGGAAGTTCACTTTTCTATATGATGGCACCATGTTGTAACAACCTCAAGATCAGAACTGCCTTCTCCAGATGACATGGTAATTCTGGAACTAGAGGGAGATGACCTGATTACATTTCTTCCTCATAACTACTTCTTGGGGCCTCATTCTACAAGGATGTGTGTCAAAGGAAAAGCTGGGTGAGTGTTTCTTTACTCTTCATTACAATGAGAGCAGTAAGTAAGTGGTAGAAGGAAACAGGGTATTCTGTAATAAATTATTAGAGAAGTCTCCGCTTAGAATGTTACTTTTGAGCCATAATGTATAATAAGCATTCAACAGATGCTTGTAGTCATAAAGGTATCTGTGGTAACCATTTATAAAAGAACATAGATTTCACTTAATGTGGTCTCTGATTACATATGTAACACCAGAAGGGGGCTAGTGGAAAGAGGATAGGGGCCTAGCAGAACAATGAAGAAAGAGACAGGAAAGGCTAAAGGGTAAAGTTTATCAATGGGTATTATATTTTGAATGAACATATCATGAAATCCATCATATTTACAATGAATGTGCATGTAAAATGTCACTCCAGATGCAATAATTTAGAGAACATGTCTGACAAAGCTTCTCCTGAGTGTGTACTCTATGAACTCCAGTATTGAAGTGCGTTGGTTTTAAATTCCTCTCACAGAACACTGAAACACCTTAAAGGGGGAAAACAAAACACACACACTCATTGGTGTACAGGAGTACTTTGATAGTATTAAGTTACATTTCAATTGTGCTTTGCTTTTTCTCATTTTACTTCTTTATCCAGTTTGCTGACTGTATGCATTTAATAAAGTGAAACTTAAGCTAGGATTATACAAGAAATATTCACTAATTTAGTGTTT

>peak_C_57

gaaaCCAAGAGCACAGCTGTTCCTACTCAACTATTCTCTGGAATTAGGGAAGTTAACTTTTCCATTTGATTGTACCACTTTGTAATCACCTCAAGGTCATAATTGCCTACCTGGCATGCCTACATGACATGGTAACTGTGGGTCTAGAGGGAGATGAACTGATGATATTTCTTCCTTATTTCTACTTCTAGGAGCCTCATTCTACAAGGATGTTTATCAAAGGATAAGCTTGGTGAGTGTTTCTTTTTCTCTTCATTATAGTGACGGCTGTAAGTACATGGTAGAAGGAAACGGGATTTTCTGTAAAATGAGGTTAGAGAACTTCTGATTAGAGTATTGCTTTTCAGCGGTACTGTTTAATAAGCATACATCAGAAGCTTTTAGACATAAAAGATATATGTGTAATGATCTAGGAAAGTACATAGATTTCACTTAATGTGATCTCTGATTATATGTAACAGTAGAAACGGCTATTGCAAAGAGTAGAGGTGCCTAGAAGTAGTATGGGTAGAGAGAAAGAAAAGCTAATAGAGTGAAGACTATCAATGGGTATTATGTATTGAATGAACACGCCATGAAATGAGTGCACATTTATATATGTGTAAAAAGTATTTGCTGATGTAATATTTTTAGGAGCACATCTGA

>peak_C_58

GCTTTCCCCAAGCATGTTTGATGGAGGTAACATTTTTATTATTTTAGCAAACGAGATGTTAATTTTTAGGTGAATCCATGAAGCCACTGTCAAGCACATATGGAGGACGCCATGAATGTTTTGGAAGCTGGATGGATACCTTGGGGAATGTAAAATGATTTTCTACTTAATTTAAATGGAATATTATTGTTGTTAGTTTTACTGTTCACAAGGACTTGTATCAATTAATCCACACACACACACAGTTAATGCCTAGAGAGAACAGCTCACAGCTGGAAAACTTGGAGCTCAGCTGTTTCTACTCCACTGTTCTCCTGGAATTAGGAAAGTTCACTTTTCCATTTGATGGTACCACTTTCTAACAACCAAAAAGTCAAAACTGCCCTCTCCAGATGACATGATAAATATGGGTGTAGAGGGAGATGAGCTAATGACATTTCTTCCATATAACTACTTCTTCGGGTCTCATTCTGCCTAGATGTCTTTCAAAGACCCAGGTGGGTGAGTGTTTCTTTTTCTATTCATTATAGTAAGAGCTGTAAGTAGGTGTTAGAATGATTCTGAAATAAATGATTAGAAATGTCTCTGCTTAGAATTTTACTTTTGAGTTATATTATACCATAAGCAAATAAAAGAGATGCTTTCAGACATAAAATTATCAGTGTAATGGTTAATAACAACATGGATTTTACTTAATTTGATTTCTTACTTTGTATGTGACAGTAGAAGTGGGTATTGGGAAA

>peak_C_59

ACTCTGTCATTGAGCTACATCCCAGCCACATGTCACTTTCTCCTGATAATGTAGGTAGAGTACTGTATCACTGTGTGCTATGTAAGAAAGATTACTTTCCTGAATGTGTTTGGAGGAGGTGGCATTTTCCTAATTTTAACACAAGAGCTATTAATTTCCAAGTGAGACACTGAAGTCATTGTCAAGTAATTATGGAGGAGGATCATAAATGTTTTGGAAGCAGGATGGATGCCTTGGGGAATCTAAAATGTTTTCTACTTCTTTTAAGTGAAATATTATTATTGTTGCTTTGTTGTGTTCACAAGGACTGGGATTAGTTAATTCTCACAGACAGGTGCAGCCTGGAGAAAACAGCTCACACAGGAGAAACCTAGATCTCAGCTGTACCTATCCTACAGTTCTCCTGGAGTTAGGAAAGTTCACTTTTCCATTTGAGGGCACCACATTGTAACAACCTCAAGGTCACAACTGACTTCTCCGGAGGACATAATTATTATGGGACTACAGGGAAATGAGCAGATGCCATTTCTTCCTTATAACTACTTCATAGAGACTCATTCTACAAGGAAGTTTGTCAAAGGCCAAGCCTGGTAAGTGTTTCTTTTTTATTCTTCACTATGGTGAAAGCTGTAAGTAGGTGATAGAAGGAAGCAGGACATTCTGTATAAAGGATTTGATAAGTCTTTGCCTAGAATATCACTTTTGAGACAAACTGTATAACAAGCATTCAATAGATGGTTGTAGACATGAAGGTATCTGCTGTAACCATTTATAAAAGAACATAGATACCACTTAATGTGATAAACTGATTATATATATGACAGTATAAGGAGCTATTGGAAAGAGGACAAGAGCCTAGCAGAATGATGGAGAGAGAGATAGGAATGGTTAAAGGGTGAAGTTCATTAATACATGTTATATAT

>peak_C_60

CTCTCTCACTGAGCTACACACACAGCACAGTTTCTCCTGATGATACAATTATAGTACCTCACTTCTGTGTGCTGTGTAAGAAAGATTACTTTTCTTAAATGTGTTTGAAGGAGGTGACATTTACTTTATCACAAGAACTGCTCATTTCTATGAAGCCAATGAAGGCACTGTCAAGTGGAAAAAAAAAACAGGAAGGATACCTTGGGGAATCAAAATGTTTTCTACTTCTTTTACATAAAATAATGTTGTTTGTTGTGTTCCCAAGTACTGGGATCAGTTATTTCACACAGACACATATGGCCTGTAGAGAGTAGCTCACACATGGGAAACTTAGAGCTCACCTGTTCCCACTTTTTAAAATTATTATTAGGGAAGTTCACTTTTCTATTTGGTGGTACCACTTTATAACAACCTCAAGGTCAGAACTTCCTTCTCCAGGTGACATGATGAGACTACAGGGAGATTAACTGATGACATTTCTTCCATATAACTACTTCCAAGGGCCACATTCTACAAGGATGTTTGTCAAAGGCCAAGCCTGGTGAGTGTTTCTTGTTCCTCTTCATTATAGTAAGAGCTGTAAGTAGGTGGTAGAAGGAAGCAGAATATTCTGTCTAAAGGATTAGAGAAGTCTCTATTTAGAATATTACTTTTGTGCCATACTGTGTAGTAAGCCTTCAACAGATACTTGTAGACACGAAGGTACCAGCTGTAAACATTTATAAAACAACATAGGTTTCACTCAATGTGATCTCTGTTTATCTATATGG

>peak_C_61

CTCCCATCTTTTTGCTTTGTAAGAAAAATGGCTTTCACCAAGTATGTTTGGTAGAGGTAACATTTTCATAATTTTAGCAAAAGAGATGCTAAATTTTAGGTGAATCCATGAATCCACTGTCAAGTATGTATAGAGGATACCATGAATGTTTTAAAAGCAGGATGGATACCTTGTGGAATGTAAAATGTTTTTCTACTTTTTAAAAATTGGATATTATTGTTACTTTTGTTTGTTCACAAGGACTTGTATCAATTAATCCACACACACAGTTAATGCCTACAGAGAACAGCTTACACCTGGGAAACCCAGAGCTCAGCTGGTTCTACTCCACTGTTCTCCTAGAATTAGGAAAGTTCACTTTTCAATTTGATAGTGCCACTTTCTAACATCCTTAAAGTCAAAGCTGCCTTCTCCAGATGACATGATAAGCATTCGTGTAGAGGGAGATGAGATAATAACCTTTCATCTATATAACTACTTCTTCAAGTCTCCTTCTGTATAGATGTCTTTCAAAGGCTCAGATGGGTGAGTGTTTCTTTTTCTCTTCATTATAGTGAGAGCTGTAGGTAGTTGTTAGAATGAAGCATGATTCTGTAATAAATTGTTAGAGAAGTCTCTGCTTAGAATTTTACTTTTGAGTTATACCAAAAGCAAATAAAAGAGATGCTTTAAGACATAAACTATCAGTGTAGTGATTAATAACAGGACATAGATTTCACTTAATTTGATTTCTTAATATATGTGAGAGTAGAAGTGGGTATTGGGAAGAAGACAGGGACCAGCAGGAAGATGAACAGAGACAGGAAAGTCTCATAGGGTGAAGATGATCAACCAGtattttatattaaataaaaatGTCATCAAA

>peak_C_62

TGTCAAGGCATGCACTCTACCAGCTGAGCTACATCCCAGTCACTGAATTGAATTTCTGCTGATAATACATTACCTCACATATGTGTGCTCTCTAAGAAAGATTACTTTTCCTGAGTGTGTTTGAAGGAGTTAATATTTTCTTAACTGTATCAAAAGAGCTGTTATTTCTAGGTGAGACCCCAAAGCCACTGTCAATTAAACATGGAGACCTCCATGAATGTTTTGAAAGAAGGAAAAATACTTTGGGGAAGCTAAAATGTTTTGGGGAAGCTAAAATGTTTTATTACTTTTTAAAGTAGGGTGTTGTTATTGTTACTTTTGTTCTGCTCATGAGGACTGGGATCACTTAATCCACACACTGCCTGGAGAGAATATCTCACACATGGGAAACCCAGAGCTCAGCTGTTCCAACTGTACTGTTCTCCTGAAATTAGAGAAGTTGACTTTTCCATTTGATGGTGCAACGTTGTAACAACCTCAAGGTCAGAACTGACTTTTACAGATGACATGATAATTATCTGTCTAGAGAGAGATGAGCTGATTCCATTTCTTCCTTATAACTGCTTCTCAGGGCCTCATTCTACATGAATGTTTATCAAAGACCCAGCTAGGTGAGTGTTTCTTTTATTCTTCATTATAGTGAAGAGGTGTTAGAATGAAGCATGGTTTTCTGAAATAAGTGATTAAAGAATTCTCTGCTTAGAATTTTACATTTGAGCCATACAGTATCTTAAGCCAATAAAACAAGTGCTGTAGACATGGAAATATCAGGATATATATATATACATACATACATAATTTcacacacacacacacacacacacacacacacacacaca

>peak_C_63

TCTATCAACTGGACTACATCCCAGTCTCAGTAGCTGAGTTTCTCTTTTTAATATATTGCCTCACTCCTATGTACTCATTAATAACCAATACTTTTTCCTAAAAGGATTTGGAGGAAGTGACATATTCTTAATTTTACCTTTTGAGTTGTTAATTATTAGGTGAGACCCTAAAGATACTGTGAAGTAAGCATGGAAGGGACAATGAAAGTTTTGGAAGTAGGAAGAATACCATGGGAATCTAAAATTGTTTTCTACTTCTTTTCCGTGGGATATGATTAATGTTAGTTTTGTTTTGTTCACAATGACTGGGATCACTTAATTCACACAGACAAATATAGAATGGAGAGAATAGTTCACATGTGGGAAATCAAGAGCTCAGGTGCTCCTACTCTAATGTTTTCCTGGAATTAGGGAAGTTCACTTTTCCATTTGATAGTGGCACTTCCTAACAACCTCCAGGTCAGAACTGCCTTCTCCAGATGACATGATAAAAATGGGTCTAAAGGGAGATGAGCTAGTGATATTTCTTCCTTAAAACCATTTCTTAGGGCCTCATTCTACAAGGATGTTTGTGAAAAGCCCATATGGGTGAGTGTTTCCTTTTCTCTTCATTATAGGTAAAGCTGTAAGTTAAGTGGTAAAAGGAAGCAGGGTTTTCTGTAAAAAAAATAATTAGAGAATTCTCTGCTTAGAATTTTACATTTGGGCTGCTAGTATAACAAACATTCAACAGGTGCTTGTAGACATAAGGTTATCAGTGTAATCATTAATAGTAGGACAGTATGATCTCTGGTTATATATGTGACACTAGCATAGAACTGTTTAGAAGAGGACCAGGACCTGGCAGAAAAAGAGAGAGAAAGACTAGGCTAGTAGGATGAAGGTGATCAATGAACAGTATATATTCACTAAGCATAATGTAGTCTAGCACTCTTTACAATGAATAAAGTTCATATGCATATAAAAT

>peak_C_64

AGGCATGCTGTCTATAAACTAAGCTACATCGCAGCCCCAATAGCCCAGTTTCTCCTGATAATACATTGTCTCACTCCTGTGTGCTCACAAAGAATGATGACTTTTCCTAAAAGAATTCAGAGTAGGTAACATATTCTTAATTTTATATATATAGACCTGTTAATTGTTAGGTGAAACCGTGAATATACTACCAAGTAAGCATGGAGGGAACTATCAATGTTTTAAAAGAAGGAAGGATACCATGGGGAATCTAAATTTGGTTCCTACTTCTTTTAAGTGGCATATATTGTTGAAAATTTTGTTTTGTCCATAATGACTAGGAACAGTTAATCCATACAGAAACATATAGAATGGAGAGTACAGCTTACACATGGGGAACCAAGATCTCAGGTGTTCCTACTTTACTGTTTTCCTGAAATTAGGGAAGTTCACTTTTCCATTTGGTAGTGGCACTTCCTAATGGCCTCAAGGTGAGAACTGCCTTCTCCAGATGACATGATAACAATGGGTCTAGAGAGAGATGAACTGGTGATATTTATTCCTTATAACTACTTCTTAGGGCCTCATTCTACAAGCATGTTTGTCAAAGGCCGAGCTGGGTAAGTGTTTTCTTTCCTCTTCATTGTAGTCAGAGTGGTAAGTAGGTGGCAGAAAGAAGCAAGATTTTCTGTAATGATTAGAGAAATCTTTGCCTGTAATATTACTTTTGAGCCATTCTGTACAACCAGCATTCAACAGATGCTTGTAGACATAAAGGTACCAGTGTAATGATTAATATAAGAGCATATATTTGTCTACGTATGATCTCTGGTTATTTACGTGACACTAGAATGGGACAATTCAGATAAGAAACAGGGCCTACCAGAGAGAGATATAGAGAAATAAGGAAAAGGAAAAAAGCATTAGGTGATCAATGTGTAT

>peak_C_65

CTTTctaggcatgtactctatgaactgagctatatctcaagcccACAGCCCAGTTTCTCCTGAAAATACAGTTACATGTTCTCACTTCTATCTGCTCTGTAAGAAACATTTCTCTCCTAAGTGTGTTTGGAGGAGGTGACATTTTCTTAATTTTATCAAAAGAGCTGTTAATTTCTAGGTGAGATCCTAAAGCCATTGTCAAGTATGTATGGAACTGACCATAAGTGATTTAGAAGAACAAAGGATACCATGGAGGAATCTAAAATGTTTTTTCTATTTCTCTTAAGTGGGTATTAATGTTGTTACTACTGTTTTGTTCACAGGAACTGGGATCAATTAATCTACACAAACAGATGTGGCCTCCAGAGAATAGCTCACATGTGGAAAACTCAAAGCTCAGCTGTTCCTACTCTACTGTTTTCTTGGGATTAGGAAAGTTCACTTTGGCATTTGAGCATAGCACTTCCTAACAACCTCAAGGTCAGAACTGCCTTCTCCAGATGACATAACTATGGGTATACAGGGAGATGAACTGATAACATCTCTTCCTTACTACTTCTTAGGGCCTCATTCAACAAGGATGTTTGTCAATGGCCCAGCTGGTGAGTGTTCTTTTTCTTTTCATTATAGTGAGAGCTGTTTATAGTGGGTAAAATAGGAACAGGATTCTGTAATAAATGATTATAGAATTCTCTGCTTAGAATATTACTTTTGAGCCATACAGTAAAATAAGCATTCAACAGATGCTTGTAGACATAAATGGGTCAGTGTAACAATTTATAAAAGAACATAGATTTATCTTAATGTGATCTCTGATTGCATATGTTACAGTAGAAGGGACTATTGGAAAGGTGATAGGGCCTATCAGAAGGATGGATAGAGAGATAGGAAAGGCTAATGGGTGAAAGTAATCAATGGCTAATATGTATTGAACAAACATGCCCTGAAACCCATCACTTTTTAAAAATGAATGTATACAGGTGTACATGTAAAAGTAGGTCCAGATGCAGTATTTTAAGGAGCATTTCTGACATAGGATTCTCCAGAGTTTGTACTCTAATAATTCTAAGAATGTGGTTCTACCTAGGTATTGCCAAATGTGTTAGTTTTCTATTCCTGTAACAGAACACTCAAATACCTTAGAGGATGATAAAGAAACACTCTTATTGGTGTGCAGGATTACCTTCATAGAATTAAATTATCTTTAATTGTATTTTCTCATTTTACTACTTTTGTCAAGTTAACTGGCTGTATGCATTTAAGTGTAAGAACCTTAAGCTAGGA

>peak_C_66

GCATGCTGGCTATCAACTGAGCTACCTCACAGCACCAACCGCCCAGTTTCTCCTGATAATACATTGTCTCACTCCCAAGTGCTCAGTAAAAACAATTACTTTTTCTAAGTGTATTTGGAGGAGGTGACATTTTCTTAGTCTGATCAAAGAGCTGTTAATTTTTAGGTAAGACCCTGAACATACTGTCAAGTACGCATGGAAAGGATCACAAATGTTTTAGAAGCATGAAGTATACCATGGGAAATCTAAAATTGTTTTCTACTTTTTTAAAGAGGGATATTAGCATTGATAGTTTTGTTATTTTACAATGACTGTGATTGGTTAACCCACAGAGACAGATATAGAATGGAGTGAATAGCTCACACATGAGGAACCCAGAGCTCAGCTGCTACAACTCTATTGTTCTCTTGGAATTAGGAAAGTTCACTTTTCCATTTGATACTTCCACTTCCTAAGGTCAGAGCTGACTTCTCTAGATGACATTATAATTGTGGGTCTAAAGGGAGATGAGCCGATGACATCTCTTCCTTATAACTACTTCTTAGGGCTTCATTCTACAAGGATATTTGTCAAAAGCCCAGCTGGGTGAGTGTTTCATTTTCAATCCATTATAGTGAACTGTAAGTAGGTGGTAGAAGGAAGTAGGATTTTTTGTAATAAATGTTTACAGAAGTCTGTGTTTGTAATATTACTTTTGAGCCACTTTGTATAACCAGCATTCAACTGTTGCTTGTAGACATAAAGTACCAGTGTAATGATTGATATAAGACCATAGATTTCTCTAACTATGACCTCTGCTTATAAAAGTGACACTAGAACAAGACTATTCAGAAGAGGAGCAGGGTCTAGCAGATAGAGAGAAAGAAAAGGATAATAGGGTGAAGGTGATCAATGGGTATTATATATTCACCGAACGTATAATGCAGGCCATGACTTTTTACATTGAATGTATTTCATATGCATTTAATAAGTTGCTCCAGATGCAGCACTTATG

>peak_C_67

CCATATTTTTGCTCTGTAAGAAAAATGGCTTTCACCAAGTATGTTTGGTAGAGGTACCATTTTCATAATTTTAGCAAAAGAGATGCTAAATTTTAGGTGAATCCATGAATCCACTGTCAATGTATAAAGGATACCATGAATGTTTTGAAAGCAGGATGGATCCCTTGTGGAATGTAAAATGTTTTTCTACTTTTAAAAAATGGGATATTATTGTTACTTTTGTTTGTTCACAAGGACTTGTATCAATTAATCCACACACACAGTTAACACCTAGAGAGAACAGCTTACACCTGAGAAACCCAGAGCTCAGCTGGTTCTACTCCGCTGTTTTCCTACAATTAGGAAAGTTCACTTTTCAATTTGATAGTGCCACTTTCTAATATCCTTAAAGTCAAAGCTCCCTTCTCCAGATGACATGATAAGTATTCGTGTAGAGGGAGATGAGATAATAACCATTCATCTATATAACTACTTCTTCAGGTCTCATTCTGCATAGATGTCTTTCAAAGGCTCAGATGGGTGAGTGTTTCTTTTTCTCTTCATTATAGTGAGAGCTGTAGGTAGTTGTTAGAATGAAGCATGATTCTGTAATAAATTGTTAGAGAAGTCTCTGCTTAGAATTTTACTTTCGAGTTATACCAAAAGCAAATAAAAGAGATGCTTTCAGACATAAACTATCAGTGTAGTGATTAATAACAGGACATAGATTTCACTTAATTTGATTTCTTATTATATGTGAGAGTAGAAGTGGGTATTGGGAAGAAGACAGGGACCAGCAGGAAGATGAACAGAGATAGGAAAGTCTCATAGGGTAAAGATGATCAACCAGTATTATATATTAAAAAAAAGTC

>peak_C_68

ATTGAGCTACATCCCAGCCACATGTCACTTTCTCCTGATAATACAGGTAGTGTACTGTATCACTCTGTGCTATGTAAGAAAGATTACTTTCCTGAGTGTGTTTGGAGGAGGTGGCATTTTCCTAATTTTAACACAAGAGCTATTAATTTCCAAGTGAGGCACTGAAGTCACTGTTAAGTAATTATGGAGGGGGATCATAAATGTTTTGGAAGCAGGAAGGATGCCTTGGGGAATCTAAAATGTTTTCTGCTTCTTTTAAGTGAAATATTATAATTGTTGCTTTGTTGTGTTCACAAAGACTGAGATCAGTTAATCCTCACAGACAGATGCAGCCTGGAGAAAATAGCTCACACAGGAGAAACCTAGATCTCAGCTGTACCTACCCTACAGTTTTCCCGGTGTTAGGAAAGTTCACTTTTCCATTTGAGGGCACCACATTGTAACAACCTCAAGGTCAAAACTGACTTCTCCAGAGGACATAATTATTATGGGACTACAGGGAGATGAGCTGATGCCATTTCTTCCTTATAAATACTTCATAGAGACTAATTCTACCAGGATGTTTGTCAAAGGCCAAGCCTGGTAAGTCTTTCTTTTTTTTTTTTTTCACTCTGGTGAAAGCTGTAAGTAGGTGATAGCAGGAAGCAGGACATTCTGTATAAAGGATTAGATAAGTCTTTGCCTAGAATATTACTTTTGAGACAAACTGTATTCTAAGCATTCAATAGATGGCTGTAGACATGAAGGTATCTATCTGCCTTAACCATTTATAAAAGAACATAGATATCATTTAATGTGATAAACTGATTATATATGTGACAATATAAGGAGCTATTGGAAAGAAGACAAGAGCCTAGCAGAATGATGGAGAGAGAGAGATAGG

>peak_C_69

tcacctgtgcctataacttgaaggtttgacttatttcattgtgtcctaaatACAGAATACTTTCTAGGCATGTAATCTATGAACTGAGCTATATCCCAGGCCCACAGCCCAGTTTCTCCTGAAAATACAGTTACATTTTCTCACTTCTATCTGCTTTGTAAGAAACATTTCTCTCCTAAGCGTGTTTGGAGGAGGTGACATTTTCTTAATTTTATCAAAAGAGCTGTTAATTTCTAGGTGAGAACCTAAAGCCATTGTCAATTATGTATGGAACTGACCATAAATGATTTAGAAGAACAAAGGATACCATGGAGGAATCTAAAATGTTTTTCTGTTTCTCTTAAGTGGGTATTAATGTTGTTACTACTGCTTTGTTCACAGGAACTGGGATCAATTAATCTACACAGACAGATGTGGCCTCCAGAGAATAGCTTACATGTGGGAAATTCAAAGCTCAGCTGTTCCTACTCTATTGTTTTCTTGGGATTAGGAAAGTTCACTTTTGCATTTGAGCATAGCACTTTCTAACAACCTCAAGTTCAGAACTGCCTTCTCCAGATGACATAACTATGGGTCTAGAGGGAGATGAGCTGATAATATCTCTTCCTTATAACTACTTCTTAGGGCCTCATTCTACAAGGATGTTTGTTAATGGCCAAGCTGGTGAGTGTTCTTTTTCTTTTCATTTTACTGAGAGCTTATTATAGGGGGTAGAATAGGAATAGGATTCTGTAATATATGATTATAGAATTCTCTGCTTAGAATATTACTTTTGAGCTATACAGTACAATAATCGTTCAACAGATGCTTGTAGACATGAATGGGTCAATGTAACAATTTATAAAAGAACATAGATTTATCTTAATGTGATCTCTAATTGCATATGATACAGTAGAAGGGGCTATTGGAAAGGCGATAGGGCCTAGCAGAAGGATGGATAGAGAAATAGGAAAGGCTAATGGGTAAAGGTAATCAATGGCTAATATGTATTGAACAAACATGCCCTGAAACCCATCACTTTTTTTTACAATGAATGTATATGGGTATACATGTAAAACTAGGTCCAGATGCAATATTTTAAGGAGCATATCTGATATAGGATTCTCCAGAGTTTGTACTCTAATAATTCTAAGAATGTGATTCTACCTAGGTATTGCCAAATGTGCTAGATTTCTATTCTTGTAACAGAACACTGAAATACCTTAGAGGGTGATAAAGAAACACTCTTATTGATGTGCAGGATTATCTTCATAGAATTAAATTATCTTTAATTGTATTTTGTATTTTCTCATTTTACTACTTTTGTCATGTTAACTGGCTGTATGCATTTAAGTGTAAGAAACTTAAGCTAGG

>peak_C_70

AAGAAAAATGGCTTTCCTCAAGTATGTTTGCTGGAGGTAACATTTTTATTATTTTAGCAAAAGAGATTAATTTTTAGGTGAGTCCATGAAGCCACTGTCAAGCACATATGGAGTATGCCATGAATGTTTTGGAAGCTGGATGTATACCTTGGAGGATGTAAAATGTTTTTCTACTTCTTTTAAATGGGATATTATTGTTGTTAGTTTTACTGTTCACAAGGGCTTGTATCAATTAATCCATACATATAGTTAATGCCTAGAGAGAAAAGCTCACATCTTGGAAACTCAGAGCTCAGCTGTTTCTATTCCACTGTTCTCCTGTAATTAGGAAAGTTCACTTTTCCACTTGGTGGTGCCACTTTCTCACAACCATAAAGTCAAAAGTGCCCTCTCCAGGTGACATAAGTATGGGTGTAGAGGGAGATTGGCTAATGACATTTCTTCCATATAACTACTTCTTCGGGTCTCATTCTACATAGATGTCTTTCAAAGATCCAGGTGGATGACTTTCTTTTTCTCTTCATTATAGTTACAGCTGTAAGTAGGTGTTAGAATGATTCTGCAATAAAGGATTAGAGAAGCCTCCGCTTAGAATTTATTTTGAGTAATATTATACCATAAGCAAATAAAAGAGATGCTTTCAGTCATTAAAGTATCAGTGTAATGGTTAATAACACATGGATTTCACTTAATTTGATTTCTTATTT

>peak_C_71

catgcactctaccgactgacctacattccagccccAAACCCCATTTCTACTGAGGATACAGTTCCTTTACCTCACCACTGTGTTCTCTGTAAGAAAGATAACTTTTCCTAAGTGTGGTTGGAGGAGGTGACATCTTTGTAATTTTACTGTCAGTGTTATTAATTTATAGAAGAGACCCTGAACTATGGAGGCTACCAGGAATATTTTGTAAGCAGGAAGGATCCTTTGGGTAATCTGAAATGTCTTTGTACTTCTGTTAAAGGAGATGTTATCATTGTTAGCTTTGTTTTGTTCATAAGGACTGGGATTAATTAATCTACACAGACAGATATAGCCTGGAGAGAATAGCTCACACATGGGAAAGCCAGAGCTCAGCTGTTCCTACTCTACTGTACTCCTGGAAGTAGGGAAGTTCACTTTTCCATTTCATGGTGCAACTTTGTAACAACCTCAAGGTCAGACCTGCCTTCTCCAGATGACACCATAGCTATGGGTCTAGAGGGAGGTGAGGTGATGGCATTCCTTCCTTATAACTACTTCTTAGGGCCTCATTCTGTAAGGATTTTCATCAAAGCTCCAGCTGGTGAGTGTGTCTTTTTCTTTTCATTAATATTGAGAGCTGTAAGTAGGTGGTAGAATAAGAACAAGATTCTGTAATAAATGTTTAAAGAATTGCATGTTTAGAATTTTACTTTTGAGCCATTCTGTAGCATAAGCAAACAAAAAAGATTCTTATAGACAAAGAAATATCAGTGTAATGATTCATAAGTAACATAGATTTCACCTAATATGGTTTCATGACAGTAGAAGGGGGCTGTTGGCAAAAGTATGGGTGTCTGGCAGAAGGTGGATAGAGACAGGAAAGGCTAGTAGGGGGAAGACAAGCAATGGGTATTATATATTGAATGAAAATGTCATGTAATCTATCACTTTTTAAAATTGGATATACACTGATAGATATTTAAAAGTAAACCCAAATGGAAATTTTTAACCAGTATATCTCACTATGAATTATCTTGAG

>peak_C_72

ctgatgcacatcccagccccACAGCCCTTTTCTACTGACAGTACAGTTGCATTACTTCACTACTGTGTTCTCTGTAAGAAAGATAACTTTTCCTAAGTGTGTTTTTAGAAGGTGATGTGTTTGTAATTTTAGCATCACTGTTATTAACTTCTAGATGACATCCTGAAGTATGGAGGCTACTAGGAAGGTGTTGCAAGCAGGAAGGATACCTTGGGGAATCTGAAATGTCTTTGTACTTCTGTTAAAGGAAATGTTATTATTGTTACTTTTGTTGTGTTCACAAGGACAGGGATCAGTTAATCCACACAGACAGATATGGCCTGGAGAGAATAGCTCACACATGGGAAACCCAGAGCTCAGCTGTTCCTACTCTACTGTTCTCCTGGAAGTAGGGAAGTTCACTTTTCCATTTGATGGTGCCACTTTGTGACAACCTCTAGGTCAGAACTGCCTTCTCCAGATGACACCATAACTATGGGTCTAGAGGGAGATGAGCTGATGACATTCCTTCCTTATAACTACTTCTTAGGGCCTCATTCTGTAAGGATGTTCATCAAAGGCCCAGCTGGTGAGTGTTTCTTTTTCCTTTCATTAATAGGGAGAGCTGTAAGTAGGTGGTAGAATAAGAACAGGATTCTGTAATAAATGTTTAAGAATTCTATGCTTAGAATTTTACTTTTGAGCCATTCTGTAACATAAGCAAGCAAACAGAACAGATTCTTGTAGACATAGAAATATCAGTGTAATGATTCATAAGTAACATAGATTTCACCTAATGTGGTTTTAAGATATTAAATACATGACAGTAAAAGGGAACTGTGGAAAGTATGGGGGTCGAGCAGAATGGTGGGTAGAGACAGGAAAGTCTCTCTTAGATCTACTCTGGATATTTCCAA

>peak_C_73

catgcactcaactgactgacatacatcccagccccACAGCCCATTTCTACTGAGGATTCAGTTCCTTTACCTCACCACTGTGTTCTCTGTAAGAAAGACTACTTTTCCTAAGTGTATTTGGAGGAGGTGACGTATTTTTAATTTTAGTATCAGTGTTATTAATTTCTAGATAACACCCTGAAGTATGGAGTCTACGCGAAATTTTTTCCAAAAAAGGAGGACTCCTTGGGGAATCTGAACTGTCTTTGTACTTCTGTTAAAAATAAATGTTATTATTGTTACTTTTGTTATGTTCAAAAGGACTGGAATCAGCTACTCCATATAGACAGATATAGATATAAGCCTGGAGAGAATAGCTCACACATGGGAAAGCCAGAGCTCAGCTGTTCCTACTCTACTGTTCTCCTGGAAGTAGAGAAGTTCACTTTTCTATTTGACGGTTCAACTTTGTTACCATCTCAAGTGAAAACTCTCTTCTCCAGGTGACACCATAATTATGGGTCTAGAGGGAGATGAGCTGATGACATTCCTGCCTTATAACTACTTCTTAGGACCTCATTCTATAAGGATGTTCATCAAAGGCCCAGGTGGTGAGTGATTCTTTTTCTTTTCATTAATAGTGAGAGCTATAAGTAGGTGGTACAATAGGAACAGGATTCTGTAATAAAAGGTTAGAGAATTTCTTGTTTAGAATTTTACTTTTGAACCATTCTGTAGCATAAGCAAGCAAACAGAACAGATTCTTGTAGGCATAGAGATAGCAGTGTAATGATTTAAAAATAACTTAGATTTTACCTAATATGGTTTCAGGAatatatatatatatatatatGTTATGACA

>peak_C_74

catgcactctgccaactaacatacatcccagccccAAAGCCGATTTCTACTGACAATACAGTTTCATTACCTCACCACAGTGTTTTCTATAAAAAAGAATACTTAAGTGTTTTTGAAGGAGGTGACATTTTTGTAATTTTAACACCAGTGTTATTAATTTCTAGATGTTACCCTGAGGTATGGAGGCTACGGGAATATTTTACAAGAAGACAGGACACCTTGGGAGAATCTGAAGTGTCTTTGTACTTCTGTTAAAGGAAATATTATTGTTACGTTTGTTGTGTTCACAAGGACTGGGATCAGTTAATCCACACAGACAGATATGGCCTGGAGAGAATAGCTCACACATGGGAAACCCTGAGCTCAGCTGTTCCTACTCTACTGTTCTCTTGGAAGTAGGGAAGTTCACTTTTCCATTTAATGGTGCAACTTTGTAACAACCTCAAGGTCAGACCTGCCTTCTCCAGATGACACCATAACTATGGGTCTAGAGGGAGATGAGCTGATGACATTCCTGCCTTATAACTACTTCTTAGGGCTGCATTCTGTAAGGATTTTCATCAAAGGCCCAGCTGGTGAGTGTTTCTTTTTCTTTTGATTAATATTGAGAGCTGTAAGTAGGAGGTAGAATAGGAACAGGATTCTGTAAAAATGTTTGGAGAATCTCTGTTTAGAATTTTACTTTTGAGCAATTCTGTAGCAGAAACGAACACAACAGATTCTTGTAGACATAGGAAAATCAGTGTAATGATTCATAAATAATGTAGATTTCACCTAGTATGTTTTAAGAATATTATATACATGACAGTCAAAGGGGGCTGCTGGGGAAAGTATGGGGTCTAGCAGAACGGTGGGTACAGACAGGAAAGGCTCTATTAAATCTATTCTGAGTATTTCAAAAATATCTG

>peak_C_75

agccccATAGCCCATTTCTACTGACAATACAGTTGCATTCCTTCACCACTGTGTTCTCTGTAAGAAAGATAACTTTTCTTAAGTGTGTTTTGAAGGAGGTGACGTTTCTGTAATTTTAGCATCTGTGTTATTAATTTCTAGATGTTACCCTGAGGTATGGAGGCTACAGGGAATGTTTTACAAGAAAGTGGGACACCATGGGAGAATCTGAAATGTCTTTGTACTTTTGTTAAAGGAAATATTATTGTTACTTTTATTGTGTTCACAAGGACTGGGATCAGTTAATCCACACAGACAGATATGGCCTGGAGAGAATAGCTCACACATGGGAAAGCCAGAGCTCAGCTGTTCCTACTCTACTTTTCTCTTGGAAGTAGGGAAGTTCACTTTTCCATTTGATGGTGCAACTTTGTAACAACCTCAAGGTCAGAACTCCCTTCTCCAGATGACATCATAACTATGGGTCTAGAGGGAGATGAGCTGATGACATTCCTGCCTTATAACTACTTCTTAGGGCCCCATTCTGTAAGGATTTTCATCAAAGGCCCACCTGGTAAGTGTTTCTTTTTCATTTTCATTAATATTGAGAGCTGTAAGTAGGAGTTAGAATAGGAACAGGATTCTTTAAAAATGTTTGGAGAATCTCTGTTTAGAATTTTACTTTTGAGCAATTCTGTAGCATAAACAAATACAACAGATTCTTGTAGACATAGGAACATCAGTGTAATGATTTATAAATAATGTAGATTTCAGCTAATATGGTTTCCAAAGGTAAGACTGTTGAGATCAATTGCAAAAAGTGTGTGGATGCTGATGATGTAAAATA

>peak_D_91

ATTCCTCAATCCATTATCTTGAGTGTAATTAAAGAATTTGTTCTTTCCTGATAAATTTCTATAAAAACTGGTACAGTCTAATATAATAATAAATTTCCATGGAGGTCTCAGAAAATGAGTCATGACCGATCTCGAACCACATAAACCTTGGAATAACCCAAAAACTCCTGATGGGGAACACAGGCTCAGTTCTTTGATTTGTCAGACTAAACTGCCCTTGTCCACACATGGGAAAATACAAGATTCCAGACTGCCTATTGATTATCTGTCTGGAAGGACTTGATGTCAGCCAGATTTTACAAGGTGAAAGCATATAATGTAATGCACACACACACACCCCTGAATATAAACCTAACATATCTCTTGATCTCCCCTAAAGAGCATCCTCTCTATAACTGATGCCAACACCACTTTACCACTTAAATACTCCCTAAGGAAACAAGGAAAACTTCTTGTAACTTTTGCTCTACTACTCATTATGATATAATAAAATCTTGGGCATCATCAATAATTACCAGGAAGTGAAATTGGTGATTTGCCTTAGGGATAAATGCATTTTGTTTCATTAAAAGTTTTCCAATATACCTATACAGTCATTTTCAATAACACGACAGATGACAAGGGAATGGACTCTCAGAAGTATTTCAGGATCTGACACTCCCCCTTTATGAGCTCTGATATCAGGCTTCATTCAGCAATCTGCACAGGACTTTATGGGAGAGGTAGGCACTTCTTTGAGTCTGTCTTAGGAAATGGATACACAAAGATGTTCTTCAAGGGGAGAGGAACTCAGAGCTATGTTTCTTCAGTAACTACTCAGGAGCTGGCATACATTTCTGTGAGCAACACCATATTTGCTGGTCCTAAGCAGGTGAGAAGTCTGCAAGTTTAAATTTCTGCTTAAAGTTCTCTTCTCCCTCCTAAAATCCAAGGTGTCCTTTAAAGTTAACATTTGTCATTACAATGTCTTTCTCTCCCTAATTTAACTAATTATGTCAATGTTTTCATTTGTCTAAAGACATGTCTATTTCATGGCTCAGCATGTATATGTAAATGTACTTTAAAGTGACATGTATATATTAGATAACTGGTTTTTAGTTGTTAGATGGAGGTTTATTCTTCCTATAGGGGAAAAAAATAAAGGTCCAGAAAAAATGTGAGCTATATAAAAGAAGTATACAAATAATCTGTGTGTACAAAGCATTTAATAGTGATGGTGTTAATGTTTTTTATTGTGGTTCACCAAAGAAGAAGATGACTCAGATATGTGCCCAGCACCAAATTCCTCAGAAAAACAAATAAAACTCAGACTGTTATGAATCAACAGAATGCTAGGATCCCTCAGAATTTTTTTCCCAAATGAATGAATACAAGTGAAATTCAAGGTTCTTCTGTGTGAACATAAAACTAGCACATTTTTAAAATAATACATTGGTTTTTATCATCATGATGAGTAGTCTGAAAATATATCAGCATTATATGTATTAATTTGGGGAGAATATTTCATTCACTCTTTTTTTTATAAAGATtttatttact

>peak_D_92

TTTGTCAGACTAAAATGCCCTTGTCCACACATGAGAAAATACAAGATTCCAGACTGCCTGGTGATGATATGTCTGGAAGGAATTGATTTCAGCCAGATTTTACAAGGTGAAAGCATATTATGTAATGCACACACACACCCCTGGATATAAACCTAACATATCTCTTGATCTCCCCTGAAGAGCACCTCTTTATACCTGATGACAACACCACTTTACCACCTAAATACTCCCTAAGGAAACAAGGAAAACTTCTTGTAACTTCGGCTCTACTACTCATTATGATATCACAAAATCTTGGGCATCATCAATAATTACCAGGAAGTGAAAATGGTGATGTGCCTTTGGGATCAATGCATTTTCTTTCATTAAAAGTTTTCCAATATACCCATACAGTCACATTCAATAACACGACAGATGACAAGGGAATGGACTCTCAGAAGTATTTCAGGACCTGGCACTCCCCCTTTATGAGCTCTGATTCATTCAGCAATCTGCACAGGACTTTAGGGGAGAGGTAGGCACTTCTTTGAGTCTGTCTTAGGAAATGGATACAAAAAGATGTTCTTCAAAGGGAGAGGAACTCAGAGCTATATTTCTTCAGTAACTACTCAGGAGCTGGCCTACATTTCTGTGAGCAACACCATATTTGCTGGTCCTAAGCAGGTGAGAAATCTGCAAGTTTAAATTACTACTTAAATTCTCTTCTCCCTCCTAAAATCCAAGGTGTCCTTTAAAGTGAACATTAGTCATTACAATGCTTTTGTCTTTCTCTCCCTAATTTACCTAATTATGTCAATGTTTTCATTTGTCTAAAGACATGTCTATTTCATGGCTCAGCATGTATATGTAAATGTACTTTAAAGTGACATGTATCCATTAGATAACTGGTTTTTAGTTGTTAGATGGAGGCTTATTCTTCCTATAGGGGAATAAATAAAGTCAGGTCCAGAAAAAGCGTAAGCTATATAAAATAAGTATACAAATAGTCTGTGTGTGCAAAGCATTTAATAGTGATGGTGTTAATGTTTTTTATTGTGGCTCACAAAATATTTAGATGACTCAGATACGTGT

>peak_D_93

ACTAATCATTATGATATCACAAAATCTTGGGCATCATCAATAATTACCAGAAAGTGAAAATGGTGATTTGCCTTTGGGATAAATGTGTTTTCTTTCATTAAAAGTTTTCCAATATACCCATAGAGTCATATTCAATAACACCACAGATGACAAGGGAATGAACTCTCTGAGGGATTTCAGAACTTAACACTGTCCCTTTATGAGCTCTGATATCTGGCTTCATCCAGCAATCGGCACAGCACTTTATGGGAGAGGTAGGCACTTCTCTGAACCTGTCTTAGGAAATGGATGCACAAAGATGTTCTTCAAAGGGAGAGGAACTTAGAGCTACATTTCTTCAGTATCTGTTCAGGAGCTGGCCTACATCTCTGTGAGCAACACCATCTTTGCCGGTACTAAGCAGGTGAGAAATCTGTGAGTTTAGATTTCTGTTCAAAGTTCTCTTCTCCCTCCTACAATCCACGATGTCTTCCAAAGTGAAAATTAGTCATTAGAATGGTTTTATCTTTCTATCCTTAATTTTCCTAATTATGTCAATGATTTCATTAGTCTAAAGACATGTCTATTTCATGGCTCAGCACTTATTTGAAAATGTA

>peak_D_94

ctattgtgaatagagcaggatgaacaggggtggttctctttattagataatgattgctcagttcATATCCTTCTCTGTATGTGCAAAATCGATTCTTTACATTTAAAATTCTATAGAAAATATCACAGTCTATTATGCTTCTAATAAGCCTCCATCATATATCAGAAAATGAGTCATGACCTACCTCAAAGCAAATGGACATTGGAATAGCCCAAGGATGTATGGAGTAAACACAGGCTTCTGTTGCTTGACTTGTCAAACTAAAGTGCCGTTTCTGAGCAGGGAAATAAGTACAGGAGTACAGAATACGTGTTGATGGCTTTCTGATGTCAGCACAATTTTGCAACAGTGAAAGCATGCAGTGTAATACAGTCCCTGGAAACACAGCTAACAAATCTCTTGATCTTCCCTGTAGTGCACCTCTCCACACCTGATGACAACACCAGTTCAGATTTAGATTTCCCCAAAGGAAACCAGGAAAACTTTTTCTAACTGCTGCTCAACTACTCACTATAATCTAACAAAGTCTTGGGCATCATCACTGATTACCAGAATGAAAATATGCTGATTTGCACTGGGGATAAGTGGTTTTACTCCTTTTAGAGGTATTCCAGAATCTGCATACAGACATGCCCAATAACAACACAGATGACACAGTAATGGACTCCCTGAAGGATGTCAGGACCAGAAACTGTCATTTTATAAGCTCTGATATCTGGTCCCCTTTAGCGATCTGCACAGTACTTCCTAGAAAACATGGACAGGAAGTCATTTCTTTGGATCTGTCTTAGGAAACAGATACACAGTGAAGATCTTTGAGGGGAAAGGAAATGGGAGCTGTTTCTGCAGTAACTATCAGGAACTGGCCTTCATCCCTGTGAGCAACCCCACCCTTGTGGAGACTGGGCAGGTGAGATTTCTGCAGCTTTATGTTTCTGCTTAAAGTTCTCTTCTCCTTCCTAAGATCTAGTGTATTCTGAAACCAGTATTAATCATTA

>peak_D_131

TCAATTCAGAACTTAGAATCTCTCAAAGGAAACTTGACTAGTCATTAAGATCTAACAAACTCTTGGCATCATCCTTAATTACCAGAAAGTGAAAATGATGATTTGCTCTGGGGATAAATGTTTTTTCTTTCATTAGAGGTATTGCAACATCCCCATACAGACACAATAAATAATATCACAGATAACACAGTAATGGGCTCTCTGAGTGATGTCAGGGCCACACACTGTCTCTTCATGAGCTCTGATAGTGGGTCTCAGTCAGTGATCAGTGAATCACTTCATGGAAAATATGGGGCAGGTAAGGATTTCTTTTGGATCTGTCTTCAGAAATAGTTACACAGAAAAGGTCATCAAATGGAGTGGAATTCAGAACTCTGTTTCTGCAGTAACTCTCAGAAGCAGGCCTCCATCCCTGTCAGCATCTCTATCATTGATAAATGTAGGTGAGATTCCTGCAATTGTAGATATTTGCTTAGACTAGATTTCTTATGCAATCAATGGTGTTTTGTAGAACCAACATTAGTCATTAGAATGACTTCCTTTTTATCTCCAGTTTACCTAATCATGTCAGTCATTTCCTTTGTGTAAAGAAATGTCTATTTCTTGGCTCAGGATTCATCTGAAAAATTCCTTCCATCTGACGACTATATGTGAGAGGACAGGTTTTTAGCTGTAGGATGATGGCTTATTTTTTTCTATG

>peak_D_132

GTCTGTTATGCTTCTAATAACTCTCCATGACTTTCTCAGAAAATGAATCATGATCTACCTGAAAACAAATAGATACTGGAATAATCCAAGGATTTACAGAGGACACATGGGCTTCAATTTTTCAGTTTGTCACACTACAAAGTCTTTGAGTATAAATGGAAATATACAAGACTACACATCACATATTGATGGTCCATCTGAAAGGATGTGATATTAGGATGATTTTGCAAGAAGAAAGGGATAAGGTATAATGCCCTGCGCCCTGCCCCCCAGGAAATACATGTAAGAAATTTCTTGATCTCCTCTGGAGTGTGTCTTTCTACTCTTGATGACAACATCAATTCAGAATCTCTCAAAGGAAACATGACTAATCATTAACAAACTCCTGGAATCATCCTTAATTACCAGTAGTGAAAATGATGATTTGCTCTGGGGATAAACGTTTTTTCTTTCATTAGAGGTATTGCAACATCCCCACACAGACACAATAAATAATACAACAGGTAACACAGTAATGGGCTCTCTGAGTGATGTCAGGACCAGACACTGTCTCTTTAGGAACTCTGATATCTGGCTTCAATCAGTGATCAGCACACAACTTCATTGAAAATATAGGGCAGGTAAAGATTTCTTTGGATCTGTCTTAGGAAATAGCTCTGCAGAAAAGTTCATCAAAGGGCATGGAATTCAGAACTGTGTTTCTGAAGTAACACTCAGAAGCAGGCCTTCATTCCTGTGAGCATCACCATCATTGATGAATGTAGGTGAGATTCCTGCAATTGTAGATTTTTGCTTAGGCTTCATTTCTTTCTTCTGCTATTAATGGTGTTTTGTAGAACCAACATTAATAATTAGATTGACTTCATTTTTGTCTCCACAGTTTACCTAATCTTGTCAGTCATGTCCTTAGACTAAAGAAATGTATATTCCATGGCTCAGGATTCATCTGAAAAATTCCTTCCATGTGATGAGTATATGTGAGAGGACAGGTTTTTAACTGTAAGATGAATGCTTATTTTTTCCTATGGACAGCAAAATAAACCAAGTAAGTTTCAGACTAAACATGATCTTTCTAAAACTCAGTTTCAAAAAGTCTTTGTGTGCAAAGAATTAAACTGTAATTGTTTTAATGGTTTCAGTTGTGGATGACAAAAACTGCAGATGAATCAATTATGTGTACAGCACCTAATTCCTTAGAAAAACAGATGAAGTTAGaaaatttataaattaataatataCTTCGGCTCCACAGACTTTTGTCCCACATATATGATATATGTGTAAATCAAGGTTCTTCTATATGGACAGAATAAGAGTGACATTTCCAGAAATATTTTGCATACAATCGATATGGTGAAGAATGTAAAAATTTATCAAAACTAGATGGATTATTTT

>peak_D_133

AATCTATTATGCTTCTAATAAGACTCCATTGTCTCAGAAAAATGAGCCATGATCTATTTCAAAACAAATGGACATTGGGATAACCCAAGAACTCATGGAGGAAATACTGGCTGCAGTTAATAGATTTGTCAGACTAAAATGTCCCTGTCTGCATACGGAAAGATAAAAGACTCCAGATCACATGATTATCACCTTTCTGGAAGGATTGGTATCAGCACAATTTTGCAAGAGCAAAAGCATAGTGTGAAATGGACACCCTGGAAATACATCTAACAAATCATTTGATCTGCTCTGGGTGCACATCTCCACATCTGATGACACAAATTCATAACTTGGGTTCTCCCAAAGGAAATATTGAAAACTTCTTGTAACTTAATACACAGATACTCATTAACATCTAACAAATTTATAGACATCATCATTAACTATTAGAAGGTAAAAATAGAGATTTCCTCAGGGGATATATGACATTTTTTCTCTATCAGATGTATCCCAAATTATCCTGTAAGACACTGATACCACAGATGACACAGTAATGGAGTCTTTGAGGGATATTACTCCTGATACACTGTGCTTTCATGAGGATAAATATCCTGGTCGCGTTCAGTAATCTGCACAGCACTCTGTGGAAAATGTGGGAAGTATAAGCCTTTCCTTGGATCTGCTCAGGAAGTAAATGCCCAGAGAGTCTTCAAAGGAAGACAGTCATAGCTGTTTTTCTTCAGTAAATGCTAAGAAGAAGGCTTTCATCCCTGTGAGCCACCTGATCCTTGATGTGATTTGGCAGGTGAGATTCCTACATTTGTAGATTTCTGCTTAAAATTCTCTTGTTCGCATGCAACTCAAGAGCATCTTTTGAAGCTGACATTAATATTTATAGTGTTTGTTGTTTTGTCTCTCTAATTTATGGAGTTATTTCATTGATTTCTTTATTGCCCAAAAGTGTATGTTTCATGGGTTAGCAC

>peak_D_134

TTTCTTAATGATTGCTCAATCCAAATCTTTGCTTGTATGTTCAGAATTTCTTCCTTACAAAGCAGATTCTCTTGATACTAGCAATGTATATTAGGCCTCTGATAAGGATTCATGAGTATTTCAGAAAACAAGTCACAATCTACCCGAAAACAAGTGGATATGGGGATAACTCCAGGATTCAGATGGGAAAGATCAGCTTCGGTGAATAATTTTGTCAGAAGTCTGTGTCCTGGGACAAACATGGGAAAGTTGGAATGAATCAACTTCCTGGGCAATTACAGAATCGGAATGACATGTTGCCTCTTTTCTGGAAGGAGTTTGTGTCAGCATAATTTTGCAAGAGTAAAATACTAGGGAGGAACATAATCCATAGAAATGTTCATAATGAGTCCCTTGTTCTACTCTTGAGTGCCCCCAATTCACATCTGATTACAAGATCAATTCAGAACTTAGATTTCCCCAGAGGAAACATGAAAACTCTTTCATAACTTCTGCTCAGGTACTCATTAAAATTTCACAATCTCCTAGACATTATCATTAAGCATCAGAAAATAAAAATGGGGATTTTTCTCTGGGGATAAATGATTTTTCCTTTATAGGAAGTATCCGAAAGACATTATGCAGACAAATGCACTGACATCACAAATAACACAATAATAAACTCTGGAAGATGTCATGTCCAGGCACCCTATTATTTTATGAGAGTGGAAACCTGAACTCTTTTGGGTTACCTTCACAGAACTGCATGGAGAATAAGAAGCCATTCATAATTTTCTTGCTTTCTTTCTCAGGCTAAGAGTGACAAAGAGAAGTTCCTCAATGAAACAGACACTATGATATACCCTTCTATAAGAGCCGTTGTCTTCTTCTTCAGCCTGTGAGCATCCTTGCCCTAAATAAGGATCAGAAGGTGGGAATCTTCCAATCATAGACTTATGCTTAAAATTCTCTTCTCCCTTTTTTAGTGTAAATTGCCTTCTGAAGCCAACATGAATCATTAGTTCTTTTTGAAGCCTGCAGTTTAATTCAAATAGGTTAATATTTTTGCTTTCATTTCAAAAAGTTATCTGATTTTTTAGACTTTCACTCAGTAGAAAACAATATTCAGTGTGACATCTGGATGTTAGATGTTGAATTTTATCTGGTGGATGGAGGCCCTCCTGCTCCTACAGAAAACATATAAACAAGTAAAGTGCAGACTAAGCATCAGCTTGCAAAGGGTATAACAGTGATTTTGTTAATGATTTTGGAGGGAAGCTGGAGATGATGCAGGTAAGACTCTGGAACCTGACACTTTAAAAGAAAAG

>peak_D_135

ATAATGTGCCCTTTGTTTTACCCTTGTGTGTTCGAATTCACATATGACTACAAAATTAATTCAGAACTTAGATCCCCAAAGGAAACATGAAAACTCTTCCCTAACTTCTGCTAAGGTACTCATTAACATCTAACGAACTATTAGACATCATCATTAAGCATTTGAAAGTAAAAATGGAGACTTGCTCTGGGGATAAATGATTTTTCCTTTATAGAAAGTATCCTTAAGACAATATGCAGACAAATGCACTGACATCACAAATAACACAATAATTAACTCTCTGGAAGATGTCATGTCCAGGCACCCTATTATTTTATGAGAGTGGAAACCTGAACTCTTTTGGGTTACCTTCACAGTATTGCATGAAGAATAAGAAGCCATTCATAATTTTCTTGCTTTCTTTCTCAGGCTAAGAGTGACAAAGAGAAGTTCCTCAATGGAACAGACACTATGACATATGCTTCTATAGGAGCTGTTATCTGGAACGGGTCTTCAGACTGTGAGCACCATCATCCTGAATATGGATCAGAAGGTGAAAATCTTCCAACCAGAGATTTATGCTTAAAATTATCTTCTCCCTTCTTCAGTGTAAATTGTCTTCTGAAGCCAACATGAATCATTACTTCTTTTTGAAGACAAAAGTTTAAATCAAATAGGTTAATTTTTTTCTTTCATCTCAAAGGAATGACTATTTTCTAGATTAGCACTCAGCAAAACACAGCATTCAGTGTAATGTATATGAGATGTTGAATTTTATCTGGTGGATGGATGCACTCGTGTTCCTTTTGAAACATATAAAAAAGTAAAATGCAGACTAAGCAGCAGCTTGTGAAGAGAATAATAGTGATTGTGTTGATAATTTTTGAGGAACTGT

>peak_D_136

CTTAAGGATTGCTCAATCCATATCTTGCTTGTTTGTCCAGGATTCCTTCCTTACAAAGCAGATTCTCTTGATACTAGCAAAGTATATTAAAGTTTCTAATAGGGATTCATGAGTATATCAGAAAACAAGTCATGATCTACCTGCAAACAAGTGGATATGGGAATAAACACAGGATTCAGGTGGGAAAGGCCAGCTTCAGTTAATAAATTTGTCAGAACTATGTGTCCTGGGACAAATTAGGAAAAGTTGGAATGAAGCAACTTCCTGGGAAATTACAGAATCTGACTGACACATTGCCAGTTTTCTGGAAGGACTTGGTGTCAGCATAATTTTCCAAGGGAGAAATAGTAAGGAGGAAAGTACTCTATAGAAACTCACATAATGAGTCCCTTGTTCTACCTTGGAGTGCCCCAATTCACATCTGACTACAGGATAACTTTGGAACTTTAATTCTCCCAAAGGAAACATGAAAACTCATTTATAACTTCTATTCAGGTACACATTAGCATCTAACAAACTCCTATACATCATCATCAAGTATTAGAGAGTAAAAGTAGAGATTTGCTCTGGGGATAAATTATTTTCCTTCATTGGAGGTGTCCCAAAGTCTCCAGAGAGAAAAATACACTGACACCACAGATGATGAAATAATAAACTCTCTGAAGGATGTCATGTCCAAGTACCCTACTATTTTATGAGATTGGAACCCTGAACTTTGTTGGGTTATCTTCACAGCATTACATGAAGAATAAGAAACCATTCAGATTTTCTTTGCATTCTTTCTCAAGCAAGGTGTCACACAGAGAACATCTTCAATGTAACAGGTACTACAATATATGCTTCTATAGGAGTTGGGTTCCTAAGCAGGCCTTCAGATGAGCATCTTCATCCTGACTCTGGCTCAGAGGTGGGACTTTTGCAACCATAGACTTCTTCTTAAAATTCTCCTCTCCCTTCTTCAGTGTACATTGTATTCTAAAGCTAAAATGAATCATTATTTCCATTTGAAGACTGCAG

>peak_D_137

CTAAATGATTGCTCAATCCATATATCACTTATTTGTTCAGGATTCCTTCTTTGCAAAGCAGATTCTCTTGATACTAGTAAAATATATTAGGCTTCAGATAAGGATGCATGAGTATATCAGAAAACAAGTAATGATATGCCTGCAAACAAGTGGATATGGAAAATAACCTGAGGATTCAGGTGGGAAACAACAGCTTCAGTTAATAAGTTTGACAGAAGTATGTGTCCTGGGATTAAACATGGGGAAAATTAGAATGAAGCAATTTCCTGGAAAATTACAGAATCTGAATGACATGTTGCTGGTTTTCTGGAAGGATTTGTTGTCAGCATAATTTTGCAAGACTGAAATAGTAGTGATAAAGCACTCCATAGAAATGTGCATAATGAGTTCCTTGTTCTACTCTTGAGTGCCCAAATTCACATCTGATCACAAGATCAATTCAGAACTTATGTTTTCCCAAAGGAAGCATGAAAACTCTTCCCTAACTTCTGCTAAGGAACTCATTAACATCTAACAAACTAGACATCATTGTTAAGCATCAGAAAGTAAAAATGGAAATTTGCTCTGGGGATAAATGGCTTTCCTTCATCAGAGGTATCCCAAAGACACCATGCAGACAAATACACTTACACCACAGATGACACAAAAATAAACTCATGGAAGGATGTCATATCCAGGAACCCTACTATTTTATGAGAGTGGAAACCTGGACTTTGTTGGGTTACCTTCACAGCATTGAATAGAGATTAAAACCCATTCTGAATTTCCTCTCATTCTTTCCCAGGCAAAGAATCACACAGAGAACTTCTTCAATGTAGCAGGTACTACAAGATATACTTCTATAGAAGCTTGCATTCCTGAGCTGGACATCAGATGAGCATCTTCATCCTGTGTCTCAGAAGGTAGGATTCTTACAAGCATAGACTTCTTAGAATTTTCTTCTCCCTTCTTCAGTGTACAGTGTCTTCTAAAGTCAATATGAATCATTAATTCCTTTTCAAGACTGCGGTTTAACTGAAATAGGTTACTATTTTGCTTGCATCAAAGAAATGTCTGTTTTCTCGATTTGCTCTTAGCAGAAAACAGCAATCAATGTAACATCTGTATGTGAGATGTTGAATTTTATCTGGTAGATGAAGGCCTCTGTGCTCCTTTAGAAAACGTATAAACATGTAAAAAGAAGAGACTAAGCATCAGCTTGCAAAGGGAAAATAATGATTGTCTTAATAACTTTGAAGGGAAGATGGAGATGATGCAGGTAAGACTCTGTCACCTGAGTCTTTTTAGAACAAAAAATAAAACTGGGAAAAATATGAATAAATGAAACTCCTTAGCAAGTGGGATTTTTCACATGGAGGC

>peak_D_138

TGGGAAATTACAAAATCTGTGTGACATGTTGCCAGTTTTCTGGAAGAATTTTTGTCAGCATAATTTTGCAAGACTGAAATAATAGTGATAAAGCACTCCATAGAAATGTGCATAATGAGTTCTTTGTTCTAGCCTTGAATGCCCCAATTCACATCTACTACAAGATCAATTCAGAAATTAAATTGTCCCAAAGGAAACATGAAAAACTTTTCATGACTTCTGCTCAGGTGCTCATTAACATCTAACAAACTTTTAGACATCATCCTTAAGCATCAGAAAGTAAAAATGGAGACTTGCTCTGGGGATAAATGACTTTCCTTCATCAGAGTTGTCCCAAAGACACCACGCAGACAAATATGCTAACAGCACAGAGAACAAAATAATAAACTCTCTGAAGGATGTCATGTGCAGGTACCCTACTATTTTATGAGACTATAAACCTAGACATTGTCGGGTTACCTTTACAGCATTGGATAGAGATTAAAAACCATTCTGAATGTACTTCCATTCTTTTTCAGGCAAAGAGTCACACAGAACTTCTTTGCTGTAAAAGGCACTACAATATATGATTCTATAGGAGCTGTGTTCCTGATGTGGCCATCAGATGATCATCTTCATCCTGTGTCTCAGAAGGTGGGATTCTTGCAACCATGGACTTCTTAAAATGCTCTTTTCCCTTCTTCTGTGTACATTGTCTCCTAAAGCCAACATGACTGATTAATTTTTTGAAGACTGAAATTTTACTCAAATTGCTTAATATTTTCTTTTATCTCAAAGAAATGTCTGTTTTCTAGATTTACTCTTAGCAGAAAACTGCATTCAATGTCATTTATGTACGTGAGATGTTAAATTTTATCTGGTGGATGGAGGCCCTCATGTTCCTATAGTAAACATATAAATGAGCAAAGCGGAGACTGAGCATCAGCTTGCAAAGGGTATAATAGTGATTGTGTTAATAATTT

>peak_E1_35

CAATGATTTTTCgtgtgtgtgtgtgtgtgtgtgtgtgtgtgtgtgtgtgtgtgtgCAAATCATTACCACAAACACTTCAGCTGTTATTCCTGAATTTTTGACTGATTGTATTTGACCCAATGTGATAACAGTAGGCAGTGCTGGAATCTGATAATTAATAATAGACTAAACTTTCTATAGAAGAGTTAAGAGAAGGATTGAGTAGAATAAGTCACAGAAACCTCTTTGTAAAGTCCTCACACAGCAGACTTTAATATTCTCAGTATGTTCTGAAATAGTTATCCTTCAGATGTCATGAGAATATAATTGTTTTTAGTTCTTCAAACACTTTGGAATGTCTCTTTGAAGACACTCACataaatttaataattaaaaaaGTACCAGAAGAGTTATTGGCTCCCAAAGCAAGTGGTGTCATTGTCACAGTAGATGTAATGAAAACTGCAATTCATAAGGGAGATGAGGTCACAGATGTTGACCTTTATCTCACAGTTGAGATGCCTTGATTTTTGCTAATGCTGGATTTCACAAATTTCAGCAGATCAGGAAGTGCACACTGAGCTGAAGTTTGAGCAGGTGAAGTCAGGTGAGCTGTTTGTACTTGGTGTGGGAAGCAACACATCAAAGGAAGGAAGTCTGGAGTAAAGATTTTATAAGGAAAA

>peak_E1_36

AAAAGAAAGATGAATTCTAGTGAGAAACACAGGTTTCATTGTACATCTTCCATATATCAAACACTTATATTCCCAGCATGTTCCTTCTTCATCAGCCTTCAGTGATTATGAAATCAAGAGATTCTTCAAGCACTTTATCTCTTTTGAGACACTCATCCTATTGAGAAATCATCTTTAGTGAATTGAAATACCTAGAAAGTCTCAGGACAGTTATTGGCTCCCAAAGAAAGTTCTGTTATTGCCATGTGATGTGACATAAAAATTACAATTCTGAGGGAGTTGAAATCACATGTTCTGTTCCTTTATCTGAGGATTATGCTCCCTTTCTCTTTCACAGTGCTGTCATTTCAGGAGCTACACACCAAGCTGATAGTTCTGCTGAGAAAAGGAGTGAGGTATGTATGCAATTTGTACTGAGTCTCTGCATTTGCAGAGAAAAAATGATAGTTTTGGTAAATGCTCAAGACTGAAGAAGGGATTCTGGGGTGAAACACGTAGATGATTCTCGGCTAAGTGAGAAGAAAATTTTATGAGTATAAGTAGATTTGGTTAAGTAAACAGTGCTGCTGGCCTGCTGCTAATTTCTGACTTCTAAATAATGTATGTTAAAATCTTCTCTGATGATGAATTTAAATAAAT

>peak_E1_38

CAGGCTTCTTGGTCATGTTTAGGGTGGTATTTGTTACTGTTCCTAGCATGTTTAAGTACCTGCAACTCTAGTTAACTTGCAACATATTGTACAAAGTGGGCAATACTTTCCAGTGCAGATTATGATGTCCTGTTTTCCTCTTACTAATAAATACTTCATTAACTATTCCTAAATTCCTTAGTGACTCTTCTTTGTTCTATATTAGTGCTCTAGAGTGAAACAGCTGTGGGATCTGATGAACCCAAACAAGGTTAGGGTCCAATTAAACACACTGGCTTCCTTACAAGACTCTCACACATCCAAACCTGATGTTCACAGAACTTTCTGTAGAATCTTCAGTGATAATAGAATCCTAAGAGACAGTCCAAAGACTTTGACTCTTTTGAGACACTCACAAGAAAAAAATAATTCCACTTTGGTAATTAGAAGCTGTAAGAAAGTCTCAGAAAAGATCCTGACTCCCAAAGCAAGTGTTGTCATTGTCAGTTCTATCTGCAGTGTAGGAGAGAGAAGGAGAGAAGCTCAGAGATAAAGGCTCTATCACTCAGCTCCCTCGGAATGGAAAACCTCATCCACTTGAACTGACTTTACACTGAGCTGAAGGTTCTGCTGAAGAAAAGAGCGAGGTGTGCAATTTGTACTGAATCTGGTAAGCAGAGAAATAAGAGAGCTCTGCTAAGTGTGGAAATCTGGAAAAGGTTTTCTAGGGTAGAAACATGCCCAGAAAAGTGTCAGCTAAGGAAGAAGAAAATTTTCTGGATTATAAGCAGATTTGCTAAAGAGAAAAATGTCCTTGACCTGGTGCTAATCCATGTGGTTCCTAATAGCATGTGATAAAATCTTGTCTGATTTTGAGCATTGGTGAATTAACCCAGTGATATATTGAATCT

>peak_E1_39

ATTTTCCTCTTACTGATATTTCATTATATATCCCTGAATTCCTGACTTATTTTTTTTTGATGAGTATTACAAAGAGGGACAGCTATGGGATATGAAAATTAATACTTTCTGTAGAACAACACAGAGAAGGTTGAAATCCAATGAGACACAAAGGCTTCTTTGTAAGATTCTTACAAATCAAATCCCGAGGTTTCCTGCACCTTTCCTCCCTGTCAACCTTCAGTAATCATGAAATCCTAGGATGCTTTCCAAACATGTTGTCTTTTGAGACACTCACAAGAGAAAATAAATGGGATTTGGTGATTATTGATAGGAAAATGAGACAGAAAAGTTATTGACTCCCAAAGCAAAAGCTGTCATTGTAGCAAAAGCTGTCAAGTGCAGGTGAGATGAAAATTATCATTCTGAGGGAGGTTCATTTATGGAGTTATGAACCTTTATCTCATGGTTTCTCTTCCTCTCTCACTTACACTGCTGACCACCATGCCAGGAGCTGCACACTGAGATGTAGCTTCTGCTGAGGAAGAGTGAGCTGTGCAATTTTACTGAGTCTGGTAAGGAGAGAAAATAGGGGAGGAATGGTAAGTGCCCATGCCTGGAAAAGGGATTCTGGAGGGAAATA

>peak_E1_40

GACCAATGATTTCTAGTGAAGATAAAGATGTCACATTTTCCTAAAAGGGTTCCAAAAGCACTGGGATCCAAAAGTTAGTACTTCACATGGTTTTGTTTAGGAGATCCCAGAGAAGACTAACTGCATCCAAAATGACAAACAAACTTCCTTCCAGAACTCTTTCACATGAGACTTCAATATTCATGGCACACTATCTTCTAGTCAGCCATCAGGGATCATGAAACTGAAGAACTTCTCCAAACACATTGTCTCTTTGGATACATTTGCAAGAAACTCATCAGTGTCTTTAATAATAATCAACAGGAAATTTTTCAGAAATGTCTTTGGCTCCCAAAGGTAGTGCAATTATCACAACTGTAGGTGATATGAAAATTGTAGTTTTGAAGAGAGTTGAGGTCAAAGGTCTCTGAAATACTATCTAAGGGCTGTGCTGACCCCTCTTCCATAGTTTTTGCTGTCATTCCACGAGCTGCACACTGAGTTGAAAGTTCTACAGACTGAAAAATTTCAGGTGAGATTCTTGTTTTTCATCCTCAAAAGCAGTGAAATCATGTAACAAAGGTCTGGTAAATGTCCACCTCTGGAAAATAGATTCTTGGTAGAAACAGAAGCCTTGTGACTTTGTTAATGATCAGGAAGACTTTATAGACTGCAGACCAAATTACTAAGGAAAAGTGATTGTCTCTGACCTATACTAATTTGTATACTATCACATTATGCAGCAAAAGTGTAACAAAATCCTCTTTCAGGCTACCTACTGAAAACTGTAAAATCTAGTAATTTTACCTGATTTTGAAGGTGTTTCAGGTTAATTCCCTCATTCAGGTTTCCATTTATTGTACATATAGAGTAATTTTGCAGTGTGACAATCTAAAATTTTGGAAAATGACTTTGTGTTTACAAATAAACCTTAGAAATGGATTTATAAAACTCATCTAATCTTAGGAAGAAGCCAAGATTGGGACTTGCCAGGGAAGTAACATTTCTAGACAGCACTACATCTATGACTGGGCAGGAGCTTTATCTGTCAAAACAATCATAAGGTGACTACTGAGACTGTATTCTCTCTTGAACTTGAAAATATTGTGTTAAAGTCAAGGTGACAATGTCATTGTGAATCTGGTAGAATTCTGGTCTAGCATGAGTACAGTTTCTGTCATTTCCCCTACTTTAGTGATATGCTGACTGCACGGGCATCTCACAATGGGTTTTCCACTTTTCATAACTGAAGTTTCCAGAATATAAAGCCTTATACATAGCAGGAAAGAGTTTAAACTTAGGTGACACCCATAGCACATATTTATAACATTCCTTTTAAATATGTAGTTGATAAGATTTCATATTTTATAGATTACATATGCATATATATTTTCACTTAGGTGTCATTTATAAGTCTTGATTTATTACTAATAGTTT

>peak_E2_95

TGTATGAAACACTCAAATATTTTAACTAATACAATTGTAAAACTAAAAGTTAATTAAGTTGATTTGTGTTGGAAGAAGTAATTAAAGACTGTGATCCAACTCAATACAGAAACTTCTCTTACTTTGTCATTTTTTTTTTACCTCAAAATTTCTAACACTTTCTACAGTACACCTGGTCAGCATTCATGGATCTAAAAAAACACAATCACTACTAAAAACACCTTATGCCTTTGAAAAACTTCAAAATAAACTCTCTCTAAAGCATTTTGTTAATTAAAAACTGGAAAATTTGCACAGATGATCCTGTCTTCCCATGCCAATGCTGTCATGTGCATTGTAGGTGAAAAGAAAACTGCAGTTCTGGAGGAAGGTGAGGTCACACAGTCACAGACTTCTACCTCAGGCTTAAGCTGGCTCTCCTAATGAACTGCACATCAATGAGTCTGAATCTGCTCAATGATCCCAGCATTCTGCAGAGGCAAGGAGCCAGGTGAGTCCTTTTTATATCAGCCTGAGTAGCAGAGCCAACCAAGAACAAAATCCTAGGTAAATGTTCACATCTCCTAATAGGAAACTAAAATAGAAAGAGATGGATGGCATGGTCATAGAGGACAAGAAAAATTCTACAACTAAGACATGATTGAAAAGGTAAAAGTATTCCTGACCTTGTGCCAGTCCTTCTCATTTCTTACTGGAATATAATTCCCTACTAAGTGTAACTCTGGATAACACCAGGATAATATAACCTCTCTTTAAAGGAGCTGAAGTATTGTGAAAATAAGACAATGTGATAACATACCTTATCTTACACAATGGGTAGAATAATCTTAAAGTATTATTATTATTATTTAACTCATCAAGGAGTTATGGTACAGAAACCACTGGAATGGAAATATAAGTAGAACACAATCCTGAGAAGTGCAGAATGTATATGTTTGTGGATTGAAAAGTACTCATATGTGACACTACATCTTTGATTGGGTACAGAGACAGTAACTGTGTCTGTATTGGTTTGCCATTAATATAATGTTAGATTTGAAGCCTGTCCCCCTTAAATGTGGGAATGCAATATGCAGGAAAAGATACAGTGTGACTATTGAACAATGTCACCCAGGACGAAATGTGTCTTGTTTTCCTTGGTTTAATCACACTCTAAGCCTGACATGATGTCACATATTCTCTCTATGTGTTCTTCATGGAATACAGGTCCGTGTTCGTGATATGATCTCAGGGTTCTTTACCTCAGATAAATTTGTATCCACACTAAGTATCACACTGGGCAATGATTAAGCTTTCACATTTCTTCAGATTGCACTTTTTTTGAATGCAACTCTTCTATTGGATGTATCATAAATATCATTATAACATCATCATCAATATAGGAA

>peak_E2_96

TCAAAGGAAATAATCATTTATAGACTTGAGTAAAACATATTTGCCTATATGTAGCATTCAAACATTTTACataataaaaattttaaatataaaaataaattattttGATTAGTGTTGGAAGAAATCAGGGAAGACCAAAATTCAAATCACCACAGAGACTTCTTGAACTTTCTCATGAATCTACCCTCAAAGATTCTTGCACCTTCCACAAGAGACCTGGTGAGCATTCAGGGATCATGAAAATACTATTAAAAGCATCTTATTCCATTGAATAAATGAACAAGAAACTTTCTCTAAATTGTTTTGTTAATTAAGAACTGAATAATTGTATAGCTGTTCCTGTCTCCCAAAGCCAGTGCTGTCTTTGCCACTGAAGGTGAAATGTAAATTGCTGTTCTGGAGGGAGGTGAAGTCAGTCACAGACTTCTACCTCAGGTTTAAGCTGGGTTTCTTATGCAACTGCTTATTAATGTGCCTGAATATGCTCAATGAACCCACTGTCCTATATAGGCAAGAAGGCAGATGAGTATTTTGTGTTTCAGCCTGCAGAATCTGCAAAGAAGAGGGGATTAGGTAAGTGTCAGGTCTGATAATAGGAAACTGCTTTAGAAACAGATCCATGGCATAGAGAGAGAAAGCACAAGAGTAATTCCATACTTAAGAAATGACTGAAGAAGTTAAACGTGTTCCTTGCTATGTTCCAGTCCCGGTCATTTCTTACTATAAGTCCACAAATAAGTTTGACTCTAGGTAACACCAGGACAATGCAGCCCATCTCTGAAGGGTCTGCATAATTGTGGTAATGAACCAATAACCAATAACAGACAAAGCAGGATGTGTAGGCtaattttaatttttaaattatcatttaatttataatGGTTTATTATGGTTCC

>peak_E2_97

AATTGGAAGGAATAACCATTTTTAGACTTGCATATAACACATTTGCCTCAAACACTCAAACATTTTTTAGTACTAAGTGTTGTAAATCTTATAAAAAGATACTATTTTGATTGGTGTTAGAAGAAGTCAGTGAAGAGTGGTGTCTACAAAGCCTTCTCTAACCTTCATTATGAATCTGTCTCCCAAGTTTAAAACACCTTACACAGGACACAAAATCAGCCTTCAGGGAGCAGGATAATACAATCATTCCTTAAAACACATTATCCCTTTGGAAAAATTCACAAGAAACTTTCTCTAAAGTGTCTTTATTAACAGCTGGAAAACGTTTATAGATGATCTTGCCTTCCAAAGCCAGTGCTGTCATTGCCATTGCAGGAAAAATGAAAATTGCATTTCTGAAGGGAGGAGAGGCCACAGAGTCACAATCCCCTAACTCAGGCTTGAAAGGACTTTCTTCCATCTCTGATTATTAATGAATCTGGATGTTCTCAGTGAACTTCACTGTTCTGCAGAGGCCAGAAGCCAGGTTGATACTTTGTACTTCTGTCAGAGTGGCAGAGCCAGCAATGAAAAGAATCCTGTATAAGTATTCATGCATGCTAATAAGAAGCTGATATAGAAACAGATCCATGGAACAGACAGTGAACACCCAAGAATAATTTCATAACTAAGAGGTCATTGATAAGTTACAAGTGTTTCTAATCAGAAGCCAGTCCCTGTCATTCCCTACTATAAAATATTCCTCCACTAAGTAAAACTGTGAGTGACACCAGCGTACTGTGGCCTATCTTTCAATATGCCGAAGGATTGTGTAATAACTCAAGGTAATAATTCACTTCACATTACATGATGTGCAGAGTATCTTTGAAGTAATATTGTTGTTTAACTTATAATGGGTTCATACAATCATGAGAGTGGAAATATAAGTAGTTCAGAGTCCTGGAAAATGATGGGTGTTAATATTTTTGTAGATTGAACACTATTCATACATGACACTATAATCTAGTTACAGAGGCAGTAAAATAGCCTGTATTGAATGGCTTTTGGCATACTGACTTTAGAAGCCTCTTCATCTTGATCGTAGGAAGAGAACAAGGAATGAAAGGTTTAGTATTCATGTTCAGAAATGACAGCCACACAATGTGTCTTGAGTTATTTTACCTGGATTAATCATACCCTCATATTAAATGCATACCCATGGCAAACTTTACACTCTTTTTTATTGTTGTTGTTTATGGAACAGAGGCCCTTGGGCATGATATTCCTGGGTTCTATTGCTCAGATAAATTTGGACTCACACCTATTTATTATCTCACTGGAAAATGTTTAAGTTTTCTCAATCTTCAGATTGTACTTTCTTACTTGGCACGCATCTCCACTATTGAATGAATCATAAATTTCCGTAATAGAGTCAGCAATGTAGGAGTTAGTAAGTAACAAGACATGTGTTGAAAACAtttaatcccagtatttggaaggcaggagcagtcatatcttttttattttgtccccaacattgtctacatactgagttcagggacagctagagctacgtagtgagactcCACCATGATTAAAATACATTTTAAATGCAAATACATGCTGAATTTTAAGGGAAATACTTTAGTAACTAAACTTTCATTCCATGGTATTATTTGTTAAATACATGTTCATATTTTATGTTGTGTTTTGTAGAGGGTAGTAGTATTGGTAATTCATGTATACTTCCTCCTATAGCTATGACCTCTACAGTGACAGTAATCCATATTTGCAGTGGTATATTAAATATCAAGGC

>peak_E2_98

TTTATTAACAGCTGGAAAACGTTTATAGATGATTTTGCCTTCCAAAGTCAGTGCTGTCATTGCCATTGCAGGAAAAACGAAAATTGCATTTCTGGAGGGAGGAAAGGTCACAGAGTCACAAACCTCTAACTCAGGCTTGAGCGGACTTTCTTCCATCTCTGATTATTAATGAATCTGGATGTCCTCAGTGAACTTCACTGTTCTGCAGAGGCCAGAAGCCAGGTTGATACGTTGTACTTCTGTCAGAGTGGCAGAGCAAGCAATGAAAAGAATCCTGCATAAATACTCAGGTATGCTTATAAGAAGCTGATATAGAAACAGATCCATGGAACAGACAGTGAACACCCAAGAATAATTCCATAACTAAAAGGTCATGGAGAGGTTACACGTGTTTCTAATCAGAGGCCAGTACCTGTCATTTCCTACTATAACTAATTCCTCCATTAAGGAAAACAGTGGGTGACACCAGGGTAATGTGGCCTATCCTTCAATATGCTGAAGGATTGTATAATAACTCAAGGTAATAATTACTTTACTTTACATGATGTGTTGACTATCTTTGAAGAAAAGTTATTTAACTTATAATGGATTCATAGAAAACATTAGATTGGAAATATAAGTAGCTCAGAGTCCTGGAAAATGATGGGCGTTAATATTTTTGTGGACTGGACACTATTCATATGTGACACTATAATCCAGTTACAGAGGCAGTGAATGAGTCTGTAATTGAATGGTTATTGGCATACTGACTTTAGAAGCCGCTTCATGTTGATCATAGGAAGAGAGCAAAGAAAGAAAGGTTTAATATTTGTGTTCAGAAATGACAGCCACAATTGTGTCTTGAGTTATTTTACCTGGATTAATCATACCCTCATATTAAACACAAACCCATGGCAAACTTTACACTCTTGTTTATTGTTGTTGTTCATGGAACAAAGGACCTTGGGCATGATATCCCAGGTACTATTGCTCAGATAAATTTTGAATCACACTTATTTTCGCATTTCACTGGAAAATGTTTAAGTTTCCCCAATCTTCAGATTGTATTCTCTTACATTGCATGCATCCCCTCAATTGAATGCATCATAAATATTACTATAATAGAGTCAGCAATATAGGAGTTAGTATGTAACAAGACCTGTGTTGAAAACAtttaatcccagcatttgggaggcaggagcagttatatcttttttattttgacaccaacattgtctacatactgagttcagggacagctagagctacgcagtgagactcCACCATGATTAAAATACATTTTAAATGTAAATACATGCTGAATTTTAAGGGAAATACTTTAGTAACTAAACTTTCATTCCATGGTATTATTTGTTAAATACATGTTCATATTTTATGTTATGTTTTATAGAGGGCAATAGTGTTAGTAATTCATCTGTACTTCCTCCTATAGCCTTGGTAGCGACAGAAACCATATTTCTTCTGATATATTAAATATCACGCCCATTTTGTGTTTCTGAAATCAGCCTTGGACATGGATCATCTTTCAGTCACGGCAGACCACACTAAGAACACTGCATTTTATTAAAAGTTCAAAAGCATAAGCATTTGTTATTATCATATGAAATATAATTTTATATTAAAGTTTCAAGTTTTTTTAAATGGTTTATGTATTGatttttattttctctgtgtgtatgct

>peak_E2_100

AAGGGGATAATGAAGGATGAATTTGACTAAAATACATTTAGATGTATGAAATACTCAGATACATTTAAGTATTAATTGCTCAAAATATTTTCTTTTTTAAATTAAATTGATTTGGTTTGAAAGAAGTCAGAGAAGCCTGAATTCAATGGGACTTACAAACTTTTCTAAAGTTCTCAATCTGACCTCAAAGTTTCTAGAACCTTCTACAGGAGAGCTAGTCAGTCATCCTCCAGGGATCATGAAAACTTGGCATCTTTTCAAAGCACATAGTCCCTTAAGAGATATTCATGAGAAACTTTAATTTTAAGACTTTTTAACTCTTAACTTTAAGACTTTTAAGTGTCTTTTTTAATTAGCAACTGGGGAAAGATTCATGAATAATGTTGTCTCCCTAAGTCAGTGCTGTTATTGTCATTGCAGGTGAAATGAAAATTGTCATTCTGGAGGGAGGTTAAGTCACAGAGTCACAGACCTTTCTCTTAGGCTTCCGTTGTCTGTGTCTCCCATGATGCTGGTTATATATACTGAGTCAGGATATATACACTGAGCCCAACCTTCTGCACAGGTAAGAAGCCAGGCGAGGATTCTTTCTTTCAGAATTTGTTGTAGAGCCAGCAAAGAAGAAAAGCATGTGAAAGTCCTCATGTCTGATAATGGCAACCTGGGGTATAAACACATCTATGGAACAGACAGACAAGGCACAAGTATAATTCAACAGCCAACAGGTGAGTGAAAAAGACAGAAATGTTTATAAACTTGTGTCAGTCCTTGTTGGTTCTTACTATAATATAAGCCCCCCACTGAGGGTGACTGTGGGTAACACCAGGGAAATGCAGCCTGTCTGTGAATATGTTTAAGGACTGTGGTCATACATCAATGTGATAATTCAAGTTACATTACATTATGTGTAGAATAATGTTGCAGATATTACTATAGGTGAACTTATCCTGGATTCACAACATAGTAAACACTCAGATGAAAATATAAGAAGCATACAACCGTGGGAAGATACAGGATGTTCTGGAGTAAAATGTAttttatgtgaatttttatgtgaatttGTCAGTACTTTTATGAGTTATAGAGACAATAACTGTATTGTATGATCCCTGATACAATAACTAGTTTGAATCCTCTCTCTTAAATTGGGAAGAGATGACTGAATTGAAGTTGAAGACTTAGTATAACAGCAATGATATCAAATGTATGTTGATTTAGTCATACTCTAATGTTAATCCTAACCTCATTATGGTTTCCAGTGCTGTTTTGTTGTTGCTAATGGAATGCAGGGCAATATACATGGAAAGTTAAGGCTCTACCCCACAGCTATGTTCATCTCCACATGTAGTTCTTTCTTTCAGTTTGGAAAATATTTATCTTTTCACATATTTCCAAGGTTTGTTTCCAAGGTTTGTTTCTCTTCTATTTGGATGCATTGCCAATTTTATTATAATAGGTTCATTAATATAAGAAGTAGTTTGCATAGAATTTTAAATAAGATATTTTAGTAACATAATTTGATCTAAAGGCATTTCAGCACACTTTATTTTTCATGTTGTGCTTTGTACAAAATAATGGTGGTACTAATTCAACTGTAACACTAGAGCTTTTACTCCTAAA

>peak_F_43

CACATTATTTATCCAAGTTTAAAGGTGATATTTGGTGACCTCTGGAGAACTGAGAAAGTAGCAACCCCTCTGAAATCTACAATATTCTCTTAAGAACTGACAGCTGGAACTTGTAATTGTACTTTGCTAATAGTCACTAGGGGAGGAAACTTCAGAGCAACATCTTTACCTGCTCAACCCTACATGCAATGTCCATTGCTACTGCATGTGAAATAATAATTGCAGGCCTGAAGGGAAGGAAGTCACAGAATCATAAATATATATCTGAGACAAAGGGTGCTTCTTTCCATATGACCTTTCTGGGGCCATCATGCTGAGATCAGCATCCTAAAAAGAAAGAGTGAAGAGAGTGTAATAGACTGCAATTTGAAAGCAGAGGAAAAAAAGGTAAAATGTGTGAGTATTGGTAGGTGATCATAGGAGAGATTAGGACATTGGACTCGATG

>peak_F_44

TCTTGCTTTCCTCTCTGGATTAAGAATTACCAGATCAACTTCAACAGTAACCTCTTAATAGAGAAGAATATTAAATTGGCCCTTGTTTTCTATCATTGCTATCAGTGGCAAACAATTTTCAGCTAAGTTTTTTTGTTTCTCAAGGCTCTGTGTCCATTGCTATTGCAGATGAAATAATAATTGCAAGACTGAGGGGAGAGGAGATCAAAGATTAACAGATTCACATTTCAGGCACTGCCCACCATTTAAGGCAACAAACTTACCAAGACTAGAGCACTGAGAATACAATTCTAGAAGAGATAGAGATGTGATTTTCTAACACCTGTCTGGCAATCAGAGTTTATGAAGAAAATACACTAACAACATTCTGGTAAGTCATCACCTGAGTAAAACTGGATCTGAGACTTAAGCTGTTTCATAACAGAGAAAGCAGAATTGCTCCTGAGCAAAGGAAGCACAGCAGCAAGAGACATGTGATTCTAGACA

>peak_F_45

ACAGCCTCACATTTTCTGCAACATTCTCTTAAGGGATCAATCAATAGCTTGCAATTGCATTGTGAAGAATATCACACTAAGGGTGCCTTTGGAAAAGTTTTACAGCTCCACACAGCTGCATACTTGCTACATTCACTGGTGAAATGATAATTGTAGTTCTGAAGGGAGATGAGGTCATGGAATCACACAGCCATAAAACAGGTACCGTCTTACTCCCTATAGCACATAGCCTGTGCTGAAAAACTATGGAACTGAAATCAGTACCACTTAAAAGATCTAGTGCTGTGTGTTTTACATTGCAGTCTGCAAGCAGAGTTTGTAAGGGCAATTCTGTGTCAAGAATGTGGGTAAGTGACCCTATCAAAAAGGGAATGGGGAGAGAAACTGAGTGTATATTGATGCTAGCCATGACAGACAGACACTCCTGAGGAATAAGGTAAGTTAGCAAAGAAATATGAGGTTCTAGACA

>peak_F_46

ggaatctcagggttgtttGATAACAGTCTCTTTTAACTTTAAAATACTCCTTTAAGAGCTGATAGGAAGATTTGTAGTTCATTATTGAACTACATCATTGAACTATTAACAGGGGAGAAAAGTTCAGAGCTTATTTACTGCCTGGTCAAGTCTACCTAGAATATCTATTGCTACAACAAGTGAAATAGTAATTGTAAGTCTAAGGGGAGATGAGGTCACAGAATCACATATCCATATCTCAGACCCCGGTACCTCCCTTCAAATGACTCTTTGAATTTTGTGGCAAGGGCACTAATATCAGCATCCCAGGAAGCGAAATGGTGCAGAAAGTGGAACAGACTGCAACCTGAGAGCAGAGAAGATTAAATATTCCAGTCTTGGTAAGTAATCATAAGAGACATGTTAcatgtgtccttattacatagtgcagcatcttctgggtatatgaccaggagtggtattgctggatcttccagtagaactatatccaatt

>peak_F_47

CTGAATTTGGCTCCATGACACCTCAGGTAATCAGAAATGGATTAATGCAAGTGTGCCTTGCTCATGTTTGCAAGACATACCAAAGAATTTCATCAACCTTATCAGTCTTCAGGGACATGAGAATATAACAATAACAGCAAGCTGCTACATTTCATTGAGATGATAGCAAAAAGGGTTTTTGATGCAAGACACCTGGGGAAAAGATACTCAGAAAAGTTTTCCCCTGCTTCATGCAAACTTGGGTACCCACTACTACTGAAGGTGAAATTTTAATTGCATGTCTGAAGGGAGGTGAGGTCACAGAATTATATGTCCATATCTCAGATACTGGCTTGCTCCCTCTAGTATATTTCAAGGTCCATAAGACCTTTGCACTAAGATCAGCATCCTGGCAAGAGAAACAGCACTTTGAATGCATCCAGTGGAGTCTGTGAGTCAGAGTCCATAAAGAAAATCCATCGGAAGACTCTAGCTAAGCATAACTTCCATGAAACTCAGCTTGGAATTGCACTATGTCACATGACAACTAGCAATTGGAG

>peak_G_101

GGCTCAATagaggtttagggtaggggcctgaggtgagaatgctgatagtagccaggactctgtaggagctacttgcaatgctctgagagtctggcagctagcatcgctttcatatattaataggcacctaaattagccatttgtcctgagtGTCTTTGGGAGGATAAAATTTTGAGATGTAGGTGATAGGGAATGACCCTCCAGGAACAAGGCCACGCAACTTGCTTATCCTTCTCCTTGCCTTTTTGACATACCCTTGGTGCTCCAAATTCTGACAAAAGTGTTCCAATTCAAATTCCTAATTGCTTCCTTGGAGTAGTGCGTGTAATTTGCACATCTTGTTTTGTTTGCTTTTGTATTGTTTTCTGACAGGAAGGTATTTAAAAGTTACTGCAGATCCTTGTTGAGAGGCTGTCATCATGGTTCCAGAGTTGAGTTGAAAATGACCAATCTTCTGGGATACAATAATAGGAGATCAGGAATCTTTAAGTCAGCACAAGTGTTTCATCAGACTTTCAGCAGAGACTCACTTCTCATCGTGACAGGTATAACCATGGTGTCTTCAAGGACTGAGGTGAGTGATCAGAGCACAGGACTAGTAAAAACTAAAGATTTTAAAAAGCTATGTTCAGAGAAGCCACTTGTGTGTTGTCAAGTGCTTCATATTTTCTTCCTGAGTGCTTCTCTTAATCTTAATTGTGTTTAAAATCCGTAGTAAAAATTTGTTTTAAAATCGCTGCTGAAAAGAAGGATGATGGAGCAaaggaaagaaggaaggaaggaaa

>peak_G_102

TACAAGGATTAATGTACAAGGCTGGGGTGAGAAGTGTTGAGAGGAATCAAAACTCTGTAGAGGTACTTACAAGGCTCTGAGAGTGTGTCCAGCTAGTATCTCCTTTTAGTGTGTTAATAGGCACCTAATTTAGCAATTTGTCCTGTGTGTCTATGTAACCaatttttttttttttttttttaatGTGGAAACTATCTGAGAGGCTGGGTGATAGGAGAAGCCCCTCTAGGGAAAAGTCCATGAAACTTTCTTATACTTTAATTGCCTTGAAGTTCAGTCTGTGTTTCACTTTGCCGTAAGTTTTCTGCTGCAAGGACTGACAAAATAGTTCCTGTTCAGATTCCTAATTGCTTCCTTGTGATAGCTCATGTAATTTGTATATCTTGGTTTTTTAAGTTATGTATTTTTTTCCTGAGAGGAAAGTGATTAAAAGTTACTGAAGATTCATGTGGAGACGCTGTCATCAGGGGTCCAGAACTGAGCTAAAAATCACCAATCCTCTGGGAAAGAAACCCAGGAGAACTTTACCCCTTTAAGTGACGGGGAGTATTGCACCGTGGTTTCAGCAGAGATTCACTTCTCAAGGTTCCAGGCATGACCATGGTGTCATCAAGCACTAAGGTAAGTGGCCATAGCACAGGAATAGTAAACATGAAGGACCTTAGAAAGCAATCTTTGGAGAAAGCAGGCATGTGCCCTTACCTTCTTCCTGAGTCTCTGTAAGTCTCAGTTGTACTTAAAATATGTATTAGGAATATATTTCAAAACCTCTGCTGCAAACAAgaatgatggaagaaggaaagaaggtaagaaagaagaagtttttttaaaagaaagaaaaagagaggggaag

>peak_G_103

AGAGCAGCTAAAAGAAGCAAAAGACAAAGTGTCAAGACAAGAAAAAATAGAaggagtagcaaaagaagaaccaagaaacaaagagaacatggtgaaaatgtctgggcttatgagcgagaagcaagggaaaagaaatgaggcttataggcaacagaggtttagggtagggaactgggctgagaagtgcttagaggagccaggactctgtaagatggagttgcttgctgagatcctggcagccagtctctgctctgacatgttaataggcaaatcagttagccctttgtcctgaatttCTTTGGGATCAAAGGAATTTGGAATACTGAAATTGTCTGGGTTGCCTAGGTTATATGGAAAGGCCCTGCAGGGACAAGGCCATGTAACTTGCTTGTACTTTAATAGGAAAGAAGTTCTGTACACCTCTCTGCCACTAATCAGGACTGACGGAAAAGTTCCTGTCCAGATTCCTAATTACTTCCTTGAAGAAGTAGCTCATTTACTTTGAACTTCTTATTTTGTTTACTTTTAAAAATTATTTTCTGTTAGAAAGGTAATTAAAAGTTACTTCAGATCCTTGTGGAGACGCAGTCATAAGTATAGAGTTGAGTTGAAAATCACCATTCTTTTGGGAAAGAATACCAGGAGATCATAAACCTTTAAGTGAGGAACAGAGTCTTACAACAGTTTCAGTACACACTTACTTCTGATAGTGACCATCCTCATCATGGGGTCATCCAGGGCTGAGGTGAGTGTCCAGAGCATAGGGCTAATAAAAACTAAAGACATTAGAAAACAATCCTAGGGGAATCCATGTTCCTGCTCTTCATGAATGCTGCATaggagaagaaggaagaaagaagaagaaaagggggtaagaggaaaaagaagaagacagacagacagacagacagacagacagacagagacagagacagaGAagggagagaaagtagggagggagaaaggtaggaagggaaagatagaggcatggaaggagagacagaggaagggaaggaggATAGAAGGAAGGAAAAGATCTCAAAGCAACAAGGTAGGGAAAAGGTTCTCCAGTATTGTTTAGCAAGTGGGGATTGGAATGGACTTTTAGGTGGAATTGCAGCTGTTTTCAACCCAGAACAGATTAGACGTTCTCAAGTTCTCCCTAAGCTGTGTAAACCCTGATCCTCCAGTAAGAAGCTTCCGAGTCTCTTATCACTAATACAACCTCATTATCTTCTATAGTTCCTTCAATTAACCTCTGTCTCTGACTCCATCTTTCTCACCCTATGTACATCTCAGTTTGCatttttttttttttttagtttttatttttaaaaGAAGGAATTACTTGGAATCTTTCTCTTTAAAAAAAAAAAAAAACTTGGGCACTAGGGATCACACCTAGGGCCTTGCAACAACATGACAAGCCATGAGATACATGCCCATCTTATGCTAAGTAGTTTGAAGTGTTATTACTTTAAATCCTTCTTCTCACAGATTT

>peak_G_104

aaagagaagcaaggggaaatgtatcccagaggctacagaggtttaggataggaagcaggggtgagaagtatggagaagaatcaggactctgtaacaggtccttgcaatgctgagagagtctggcagctagcatatgctctgatatgttaatagacatctcagGAAGCCATTGATCCTCAGGGTCTTTGGGACCAAAGAAGGTTGAGTCATTTAAACTCTCTAGGATACTGAGTGATAAGGAATGGCCCTCCAGGGGCAAAGCCATGAAACTTGCTTATACTTCATAGCATTGATGTTCCTTCTGCATGCCTTTCTCTACCATACTCTTTGTACTCTAAAGACTGACAGAAAAGTTCCTATTCAGAGTCCCAATTGCCTTCTTGTAGTAGCTCATGTAATTTCCACTTTTTGTTTTGGAAAGTTCTGTCAGTAAGGTGATTAAAAGTTACTGCAGATCCTTGTGGAGAGGTTGTCATCATGGATACAGAGTTGACTTGAAAATCACCAATCTTCTGGGAAAGAATACTCAGAGATCATGAACCTTTAAGTGAAGAAGAGTGTCTCACCACAGTTTCATCAGAGTCTCATTTCTCATCGTGTCAGGCATAACCATGGTGTCATCAAGTACTGAGGTGAGTGACCAGAGCATCGGACTAGTAAAAACAAGATGTTAGAAAGCAATGCTTAGAGAAGCCATTCGTGTGCTCTCCAAAGCATCTTATTTTTTCCTGTATCTCTTAATTGTAGCTGTATTTAAAGTCTGTATGTATTAAGAACTTATTTCAAAATCACTGTTGCAGAAAAGAATGATAGAagagagaaaggaaggaagggagggaggaagggagggagggagggaggagggaagaaaaggagtggatgaaagagggaggaatggagggaagaagggagagaggaagcaagagagag

>peak_G_105

gagaaaaaacaaaagaaggaCCAAGAGACCAAGAGCACATGGTGAAAATGTCTGACTTTATAAGAAAGACATGCTGGGGGAAAGAAATGAGGCACAAGACTTACAGAGGTTTAGAATATGGGGATGGAATGAGAGGTGCTGAGAGGAGTCAGGACTCTGTTAGAGGTACTTGCAATACTGTGAGACTCTGACATACAGCATTTGCTCTTGTGAAAGGCACCTTATTTATCTCTTTGTCTCAAGTGTCTTTGAAAGAAAAGGACACTGGGATGCTGAAATTGTCTAAGATGCTTGGGTGATAAGGAAACTCCCTCCTGGGGCATGCCCTGCAACTTGCTTGTATTTTAATAGCTTTGAAGTTCCTTCTGCATGTGTCAGTGCCACAACCTTAGTGCTCCAAGGACTGGCAGAAAAGTTTCCTGTCCAGATTCCTAATTGCTTCCTTGGAGTAGCTCATTTACTTTGAACTTCTTGTTTTGTTTCATTTTGTATTGTTTCTGACAGGAATGCATTTAAAAGTTTCTTTGGACCTTTGTGGAGATGCTGTCATCATGGGTCTAGAGCTGAGTTGAAAATCACAATTCTTTTGGGAAAGAATACAAGGAGATCATGAATCTTTAAGTAGGGGAGAATGTCTGTCTCACCAGATTTTCAGCATACACTTCTCATGGAGACCATCTTCTTCATGATGTCATCCAGTGCTGAGGTAAGTGACCAGAGCATAGGACTAATACAAACTAAAACATTTGAAAGCAATCCTTGGGGAATCCATGCTCTTGTTTTTCATGCATTTTGCTCTCTTCCTGAGTGTTGCTCTTAATCTTAGTTGTACTTAAAACCTTAATTAACAATGTATTTCAACAGCTCTGTTGAAAAGAAGGAAAGACTGAGAAGAGgaaagaaagaagaaaagaagaaagaaagaaaagaaggaagaaagaaggaa

>peak_G_106

caagaatacatggtcaaaatatctgggcttatagaaaagagaaactgggagaagaaaagacaaattaagaaaagtttagaatagcaggagcaggagagagaagtgctaagacaagccaggactctgtaagaggcacttgtaatgctatgagagtctggcagccagaacctgctttgatatgttaataagcaccagagttagtcatttgtccagagtGGTTTTGGGACAAAAACATTTTGAGACTTTTAAACTGTGTATGATGCTGAGTAGGGGGAAAGGCTCTTCAGGGGCAAGGCCATGCAACTTGCTTATAAGTTAATAGCAGTGAAGCTCTGTCCCTTCTACACACTTCTCTGCCATAAACTGTGCCCCAAGGACTGACAGAAAAGTTCCCTTTCTGCTTCTTAATTGAGTCCTTGAAATTGCTTGTGTAATTTGCACTACTTTTTTGTTTCCCACTAGGAACTCACAACCAACGTGTTTAAATTTTACTTCATACCCTTGTGGAGATGCTGTCATTAATAGATTGTTAAATTGATAATCACCAATCTGCTGGGAAGAAACAAAAGGAGGTCATGAACTTTTAAATCTGGACTAGTGTCTCAGTACAGTTTTAGCAGATCTTTGATGGTGACACACACCATCGTGGTGGCATTGAGCAATAAGATGAGTGACCACTGCATAGAAATAGTAAGAACTAAATACTTTTTTTAAAAAGTCATTGAAGAAGCCATGCTCCTGTTCTTTGATGTATATTATTTTCTTCACCAAGTACCTTTCTTTATATTAATTTCACTTGAAATCTGTATTATGAAATACTTACAAAAAACTCTGCTGAAAAGAAGGAAGTAAGAACAATAGAAGGAAAGCAGAAAGTGAAACCAGGGAAGAGAAAAAGAgagggagggtaggaaggaaggaaggaaggaaggaaggaaggaaggaaggaaggaaggaaggaagACCAAAGGTGACAGAAAAGGCAGGATAGGAAAACAGGGCTCTGGTATTCGTTTACGGAGTGGGAAGTTGAATGGGTTTTTGATGAAGTTGAGTATTTACAACTCTGGGCAAAGTCCACTGTCTCCAGTCCTCCCTAAGCTGTGTGACCCTCTTCTTCACATAAACTGCTACTGATTCTCTCTTATCACTGATATCACCTTATTTTCTTCTTTCCTTTCATCACTTTGCCTCTGTCTCTTTTTCATCATCTCCATATCAATTATCTTATCACTGTCTTTTTTTTTTTTTTGCACAATTCTATCTAGTTCTTAGTTTTAAAAGAAAGATTTATTTGGATTCTTCCTTTTTCTTT

>peak_G_107

atttgaatggcaggggttgtgctgaaaggaaccaaaccctgtaagaggttgtttgcaatgctgtgagaatctggcagacagaatctgctgatatgttaataggcccctcagttagccatttgtcctgagtatctttgggactaaacaCACTTGAGATGTTGAAACTGTCTAAGATTCTGGATGACAGGGTAGGACCCTCCAGAGTCAAGGTCATGAAATTATACTTTCATAGCAATGAAACCATGTGCCTTCTGCATGCCTCTATGTCATCCTGTGTTTCAAAGGCTGAGAGAAAAGTTTCTATTCTAATTCCCAGTTGCTTCCTTGGAGTTGTTCCTGTGATCCATACTACTATTTGTTTTGTTTTCTGACAGGAACTCAGGACCAAGGTCTTTAAAAGTTACCTCAGGCTCTTGTGGAGGTGCTGTCAACATGGTTCTAGATCTGAGTTTAAAATGACCTGTCCTCTGGGAAAGAATATCAGGAGATTTTGAACCTTTAATTGAGGGATAGTGTCTCACCACAGCTTCAGCTGACTCACTTCTCACAATAGCTACTACAGTGGTGTCATCCAGCATTGAGGTAAGTGACTAGTGCACAGGAGTAGTAAGAACTGAAGACATTAGGAAGCAGGTTTGGGAAAAGGCATGCTAGTGCTACTGGGAGCATCTTAACTTTTTCACAACAGTTTCTCTTAATTTTAGTTGTAGTTAAAATCTATATTAAAAATTACGTCAGCTCTACTgaaacgaaggggaaaaggaagggaataaggaaggaagcaaaaaaaagggaaagaaaaggaaggaagaaaggaaggaaggaaggaaggaaggaaggaaggaaggaaggaaggaaggaaggaagaaggaagaaggaaggaaagaaggaaggaagagagggagggagggaagaaggaaaaaggaagggggaagaaagaaataaagtaagtaaggaagaaaggtgaaggaaagaaagagagaaagaaCTCAAGGCATACGTAGTAGGAAAAGGGGACTGTGGGTTGTTTTAGGCATTGGAACTTGAATGGGCTTTTGGATGAAATTGGACTATTTACAACTTGGGGCAAAGCAGACTGTCTCCAATACTCCCCACATTATGTGAACCCTTTTCCTTTTGTAGAAGGCTACTGAGTTTCTCTTATCACAGATATCATCTCATtttcttctttcctttcttcacttagcctttttctctttctcttctctctgtttgtctctgtttccctttacctctttctatttttttttTGCATGTCTCCTTCTAAATTTTAGACTTTCAAAGAAAGGTTTAATTGGATATTTTTATTTTTATTTACTGATGACAATGGGATC

>peak_G_108

ggaaagagaaacaagggaaaataaatgaggcttgtggactacagaagtttagggtaagggactaggaagagaagtgctgagaggagccaggactctgtaagagggaattgcaatgctgttagagtctggcagccagaatctgctttgtttaatgaataggcatttcagctagccatttgtcctgagtGTCTTTGGGAACAAAGGAGTTTGAAGTGCTGAAACAGTCTAGAATGCTTGGGTGATAGGGAATGGCCCCCTAGGGGCAAGGCCATGCAACTTGCTCATACTTTAATGACACCAAAGTTCATTCTGCATGTCTATCTGACTTAACTTTTCTGCTCCAAGTACTGTCATAAAAGTTCAGCCTTGATTCCTAATTGCTTCCTTGGATAGTTCATTTAATTTTAGCTTCTTGTTTTGTTAAGTTTTGTATTGTTTCTGACAGGAAGATGATTACAAGTTACTTCAGACCCCTGTGGAGATGCTGTCATCATAGGTCAAGTGTAGAGTTAAAAATCACCAATCTTTTGGGAAAGAATACTAGATGGTGGCCCTTTTAAGTGAGGGAGAGTTTGTCACCAAAGTTTCAGCAGAGACTCATTTGTGGCGGTGACTCTCTTCATCATGGGGTCATCTAGGGATGAGGTGAGTGACCAGGGCATAGAACTAGTAAAAGTTAAAGACAACAGAAAACAGTCCTTGGAGAATCAATGCTTCTGCTCACCATGTATCTTATTTTCTTCCTGAGATCCTCTTTTAATTGTATGTAAAGTATGTATTAAGAATTTATTTAAATATCTTTGCTGAAaaggaaagacagaggggaggaaaaaaagaagaaaaatagaaaacaggaagaaaaggaggaagagaagaaggaaggaacaatataaggaaggaaAACAGTTTGAGGCATAGATAAGGAGGGCAAAAGGGACACACACGTGGTTCAGGAAGTGGGAATTAGCATAGAGTTTTGTGTGAAATCATGGCTATTTTCAACCCAAGACAGAGTAGACTGTCTCATGTGAACACTGTTCCTCCTGTAAGCTACTACTGAGTCTCTCTTTTCACTGATACCACCTGATTGTCTGCTTTCCTTTCTTCATTta

>peak_G_109

ATTAAGTATGGGGAAAGGAAGTAAAACTCATGAGCTAGAGGGGTTTAGGGTAGGTGGCAGGGACAAGAAGCAGAGAGAAGCTAGGACTCTGTGAGAGGCATTTGCTATGCTAAGAGTCTGGCAGTCAGAAAATGCAGTTGACTTGGCATCTTAATTAGCCATTTGTCCTGAGTATCTTTGGGACTGAACAATTTTGAGGTATTGAAACTGTCTCAGATGCTGGGTGATAGAGAAATGATCTCCAGAGGCAAGGCCATGCAACTTGATTATATTTTGATAGCAATGAAGTTTCTTCTGCATGCCTCTCTGTTCTCTAAAGGATGACAAAAATGTTTCCATTGTGATTCCTAATTGCTTCCTTGGAGTTGCTGATGTAACTTGCACTTTTTTGTTTTGCTTTATTTCCTTCCAGAAACTCAGGTCCAAGGTGTTTAAAAGTTACTTCACACCCTTGTGGAGATGCTGTCATGAACTTAGAGTTGAGTTGAAAATCACGAATCTTCTGGGATGATCATGAGCTTTTACATCAGGGTAAATATCTCTCCACAGTTTAGCAGAGACTCACTTGCCATTGCAACACACACCATCATAATATCTTTGAGAACTAAGATAAGTGACAAGAGCATAGAAGTAGTATGAGCCGAAGGCTTTAAAAAACACTCCTTGAAGAAGCCACACTCCTGCTCTTAGATACATCTTCATTCCCCCCCCAAACACCTTTATTAATGTTAGTTGTACTTAAAATGTATATTACAATTCTTCTCCTACAGCTCTGCTGAAATgaagaaagaaaagaaggaaggagggtaggaagaaagagaaagaaaagaaagaaCTCAAAGTAGAGAAGGGGACTCTAGTATTGATTTAAGAAATGGATACTTGCTTGAATAGGATTTTGCATGAAATTGGAATGTTTTAACTCAGAGCAGAGTTGACTGTCTCCAATCCTTCCTAAGCTGTGAACCCTGCACCTCCCATGAGCTGCTACTGAGTCTCTCTTACCACTGATATCACCTCATTTTCAGGTATGTTTGACTTACTCTGTTTCTATTGTCATTGCCACTGTCAAGAGTTTCACAAGCTATCATATATCTGCTTGATGTTAGGGATTTCTTTTAATACATGAAACCCAAATTAAAAGAACAAAGCATGGCCCTACCACATAGTACACCTTTTCAATACCCTTCCAATTCTTCCTTATAGACAAAAAGCAAACTCATCATTTTGACTGTAAAATTGATATTACTAACCTAAGAGCCCATGACAGTTATGGCTTTAGTTGACTGTGATTACCATGCAACATAGCCTGAACTTCTTATATATTAACCACAATCCCATTACATTTAAGATTAGAACTAATGTCAAACTACAGACATTTATTATGATGATCACTGTGagtgtacagttcagtaatgtgaaaaataatcctaatgtactacagctggtctccagaagatttcatcacaaacccaaatctatttgcccatgaaTAATTTTCTCCTTGCATCCCTGTCAATCATCATTCTAGTGTCTTCTGAAATTTCATTACATAATTAATATTTTGTTGGGATGGCATGCCCACCTTAAATAGAATACATGTGTAGAGATGAGAAAATATATTTTGGAA

>peak_G_111

AAGAAAGGAAATGAAGCTCATGAGCTAGAGAAGTTTAGGGCCAAGAAGCAGAGAGAAGCCAGGACTCTGTAAGAGGTATTTACTATACTGTAAGATTCTGGCAGTCAGAAAATGCAGTTAATTTGGCTTCTCAATTAGCCATTATCCTGAGTATCTTTGGGACCAAACAATTTTGAGGTGTTGAAACTGTCTCAGATGCTGGGCGATAGAGAAATGATCTCCAGAGGCAAGGCCATGCAACTTGATTATATTTTGATAGCAATGACTTTTCTTCTCCATGCCGCTCTGCCATACCCTTTGTTCTCTAAAGGATGACAAAAATGTTTCCATTGTGATTCCTAATTGCTTCCTTGGAGTTGCTGATGTAACTTGCACTTTTTGGTTTTGTTTTATTTCCTTCCAGAAATTCAGGTCCCAGGTGTTTAAAAGTTACTTCACACCCTTGTGGAGATGCTGTCATGAATTTAGAGTTGAGTTGAAAATCACCAATCTTCTGGGATGGAATACCGAGGAATCATGAGCCTTTAAGTCAGGGAAAATAACTCATCATTGTTTAGCAGAGACTCACTTGTCATTGCAGCATGCACCATCATGACATCTTTGAGCACTAAGATAAAGTGACCAGAACATAGATGTAGTAAGACCTAAAGAATCTAAAAAACACTCCTTGGAGAAGCCACACTCCTGTTCTCAGATACATTTTATTTTTTTCCCAAATGTCTTTTTCAAATACTAGTTGTACTTAAAATGTTTATTATTAATTTCTTTCTACAGATATGCTGAAAGGTCGAAAGAAAAGGAAGGAGTAAAGGAAGAAAGAAAGAAGTCAAGGTGGGCAAGAGTAACTTGATTTAAGGAGTGGGAACTTGAATGAGTTTTTGTATGAAACTGAGATATTTCAACGCAGGGCAGAGTAGACTGTCTCTAATCCTCCCTAAACTGTGTGAATCCTGTTCCTCTGATAAGCTGCTACTGAATCTTTATCACAGATAACACCTCGTTTTCTTCTCTCCTTTCTTCCCTTAgcctctgtccctcatctctgtcttgtctctgtatctttctacctctctccatctctttctgtgactcttttcctAAATGTT

>peak_H_1

GGAGATGTGGGAAGCTCAGTACACAGAAACTTGGTAGGTAGTTAATTTTCTCACATCCTTCCTCTCAAAATAGTTAAACCCTAATTAAGCTGGAAATAACTTCAGAGCAAAACAGTTGCTGTACCTGTGGGAGAGAGGGCAGCTATACTTCAGGGACTGAGGCCCTGTGGCCTTCCTTCACCCTGACACTGTGACAACATGAAACCTGAAGTCTGCAGTACCTGATGGCAGCCAAGAGCCCCACAGATCCAGACAGTACAAATATGACCTGGCAAGTGTCACCTTCCCAGAGCTGCTCCAGAGCCTGTCTGCAGCACAGAGGTCCAGGTGAGTACATGGAGGTAAGATCTTAGATGCCCTCAGCTGTGCCATGGAGACTAAAGCTGAAGTCTCTCTCTGACTAAGACAGAGAGGAGAGGAGAGGCTCTATGAGCCTTGGGTCCTCCACTTTCCTTCTGACCCTTACAAGGCTCATCCCTGGGTTTAGAAAGATCAAAGGAGATGAGTTCCTGGAGATACAGAAATGGGACGGAAATGCTGAGGCTTTGCTCTCCATCCTCAGGCTGGAGTCAGCACAGAGCTTTGGGTGAGTCTTTGC

>peak_H_2

ggagatatGGGAAGATCAGTTGGTTACTTGTTCATTTTCCCACCTCACATCCTTCCTCTCAAAATAATTAAACACTAATTAAGGTGGAAATAACTTCTGAGCAAAACAGATTCTGTAACTGTTGGAGAGAGGGAAGCTATACCTCAGGGACTCAGGCCCTGAAGTGACCTTCCTTTCCCCTGACACTGTGACAATATGAAACCTAAACTTTGCAGTACCTAATAGCAGCCAAGAGCCCCACAGATCCAGACAGCACAAATATGACCTGGCAAGTGTCACCTTCCCAGAGCTGCTCCAGAGCCTGTCTGCACCACAGAAGTCCAGGTGAGTACGTGAAGGTAAGATCTTAGATGCCCTCAGCTGTGCCATGAAAGCTGAAGTCTCTCTCTGACTAAGACAGAGAGGAATTGAAAGGCTCCATGAGCCTT

>peak_H_4

TCAGGAGATGCAGGAAGTTGGTTGGTTGTTCATTTTCTCATCTCACATCCTTTCTCTCAAAATAATTAAACCCTAACTAAGCTGGAAATAACTTCTGAGCAAAACAGATACTATAACTGTGGGAGAGGGGTAAGCAGTACTTCGGGGACAGAGGCCCTTCTGTGACCTTCCTTCCCCCTGACACTGTGACAACATGAAACCTAAAATCTGCAGTACCTGATGACAGCCAAGAGTCCCACAGATCCATACAGTACAAATATGACCTGGCAAGTGTCACCTTCCCAGAGCTGCTCCAGAGTCTGTTTGCACCACAGAGGTCCAGGTGAGTACATGGAGAGAAGATCTTAGATGCCCTCAGCTGTGCCATAGAGAAATTCATTCAGGCTAAAGCCTCTCTGATTAAGCCAGGGAAGAGTTGAGAGGCTCCTGCACATTTCTTCTGACCCTGGCAAGGCTGGTCTCTGACTCCAGAGATCTCAAAGAAGGATAGCACTGGAGATAGATAAGAGGGACAGAAATGCTGAGGCTTTGCTATCCATCCTTAAGCTGGATTCAAGACAGAGCCTTGGGTGGGTCTTTGATTAAGGTTACCTCCTTCGGCTTTGTTTGTTTTTTTTAAGGAGTCAGTAAGAAAAAGAAGATTTTCAGATGTCTATCAAAACT

>peak_H_10

AATTTATCATGTCCTCTACAGAAAGAAGGACTTGAGAAAGAATGGATCTCTTTCTGTATCAGAAGCAGGACTCTATTCTGATCAATACATTTGGTTAAGCAGTGTAAAGAGAGGACCAGGAACATGGGGACTCTAGATATAGAAGACTTACTGTTTTTCACTTTGTCCCTTCATATGTTTCTATCATACAATGAAAATGTAATTAATCCAGAGGCAACTCTTGAGGCAAACAAATGTAGTCATTGAGGAGGGAGAGGGAAAGTCATCCTTTAGGGACTGAGGTTCCACAGAGGGCTTCCTTCTCCCTGACACAGTGATGTGTTAACATCTTGAGGATTCTTTATGGTGACAAGTGCTCTGCAAACCCTAAAGGTACAAATGTGACCTGGGAAGGCTCACTTTCCAGAAGACCCAGAATCTGTCTGCCCTGATCACTGTGGTGAGTAGACATCTCAGTTTCTTTCACTCAGGTGTGCATGGAATATTTTATTTAAAGATTCACATCTTGCTCTCTTTGACAGAGCTAAGGTATAGAGGAGAGATGTTGAGCCTGAACCCCTCCACTCTTCTTCTGACACTTAAAGCTCAGAATAACTGGGGCCAGTTCCAGGAAACCTGATTCCAGGATGCACAGCTACATCACTCTCTGTGTTTAGGGTCTGCTGTCATGACAGAGTTTCAGGGTGGATCTCTGTTTGATGCTCCCTCTTTTCTTCCAGCAACTAGAACTAAATAGAAGTTTCTTAATTTTCTGTGGAAAATAACTATTT

>peak_H_12

gGAGATTCAGGAAGCTCAGTACACAGAACTCAGTTAGTTGTTCATTTTCTCACCTCACATCCTTCTTTTCAAAATAATTAAAGCCTAATTAAGTTGGAAATAACTTCTGAGCAAAACAGATGTCGTAACTGTGGGAGAGAGGGCAGCTGTACTTCAGGGACTGAGTCTCTACAGTGGCCTTTCTTCCCCCTGACACTGTGACAATGTGAAGCCTGAAGTCTGCAGTACCTGATGGCAGCCAAGAGCCCCACAGATCCAGATAGTATAAATATGACTTGGAAAGTGTCACTTTCCCAGAGCTGCTCCAGAGCCTGTCTGCACCACAGAGGTCCAGGTGAGGACGTGGAGGTAAGATCTTAGATGCCCTCAGCTGTGCCATGGAGAGATTCATTAAAGCTGAAGTCTCTCTCTGACTAAGCCAGGAAGTATTTGAGAGACTGTGTGAACCTTGGGTCCTGTACTTTCTTCTGATCCTAACAAAACTGATCCATGCGACCAGAAAGATCAAAGGAAACAAGTTACGGGAGGTACAGAAATGAGACAGAAATGCTGAGGCGGCATCAGTATCCATCCTCAGGCTGGAGTCAGGACAGAGCTCTGGGTTATCTTTCCCTAAGGTTATATCTTTTCCATTT

>peak_H_13

gGAGATGCGAGAAGCTCAGTACACAGAAACTTGGTTAGTTGTTCACTTTCTCACCTCACAGTCTTCCTCTCAAAATAATTAAAGCCTAATTAAGCTGGAAATAACTTCTGAGCAAAACAGATGTTGCAACTGAGAGAGAGAGAGAGAGAAAAGGAAACTGTACTTCAGGGACTGAAGCTCTGCAGTGGCGCCTTCCTTCCCCCTGACACTGTGACAGCAGGAAACCTGATGGCAGCCAAGAGCCCCACAGATCCAGACACTACAAATATGACCTGGCAAGTGTCACCTTCCCAGAACTGCTCCAGAACCTGTCTGCACCACGGAGGTCCAGGTAAGTACACAGAGGTAAGATCTTAGATGCCCTCAGGTGTGCCATGGAGAGATGCATTAAAGCTGAAGACCCTGTCTGATTAAGACAGGGAGTAGTTGAGAGGCTCTGTGTACCTTGTGCCTTCCACTTTCCTTCTGACTCTAACAAGGCTGATCCCTGGCTCCAGAAAGATCAAAGGAGATGAGTTCTAGGGGATACAGAAATGGGACAGAAATGCTGAGGCACTGTTGTCCATCTTCAGGGTGGAATTGGAACAAAGCTCTGGGTAGGTCTTTACCTGCTTACCATTCATTAGAGGAATCAGTATGAT

>peak_H_16

ACAGAACATGGTTAATTGTTAATTTCTCATCTCACATCCTTCCTATCAAAATAATTAAAACCTAATTTAGCTAGAAATAACTTCTGAACAAAACAGATGTTGTAACTGTGGGAGAGAGTACTTCAGGGACTGAGACCCTTAGAGGCCTTCCTTCCTCCTGACACTGTGACAACATGAAACCTGAAGTCTGCATACCTGATGGCAGACAAGAGCCCACAGATCCAGAGAGTACAAATATGACCTGGAAAGAGTCACCTTTCCAAAGCTGGTCCAGAGACTGTCTGCAGCACAGAGGTCTAGGTGAGTACATGGAGGTAAGATGTTAGATGTCCTTAGCTGTGTCATGGATTCATTAAAGTTAaaatctctctctga

>peak_H_17

AGCTCAGTTCACAGAACTTAGTTGTCCATGTTCACACCTCACATCCTTCCTCTCAAAGTAATTGAACCCCAATTAATCTGGAAATAACTTCTGACCAAAACAGATGTCATAACTGGGGAGAGAGAGAAGCTGTACTTCAGGGACTGAGGCTCTGCAGTGGCCTTCCTTCCCCCTGATACTGTGACATGAAACATGAGGTCTGCAATGGCAGATGGCAGCCAAGTGCCCCAGAGATCCAGGCAGTACAAATATGACCTGGCAAATGTCACCTTCCCAAAGTTGCTCCAGAGTCTGTCTGCACCACAGAGGTCCAGGTGAGTACATGAAGGTAAGATCTTAGATGCCCTCATCTGTGTCATAAAGTTTCATTACAGATGAAGTCTCTCTCTGACTAAACCAGGTAGTAGTTGAAAGGCTCTCTGTGCCTTGCGTCTTTGACTTTCCTTCTGACCCTAACAAGGCTGGTCCCTGTGTCCAGAGAGATCAAAGGAAACAAGTTCTAGGAGACACAGAAATGCTGAGGCATTGCTGTCCACTCTCAGGCTGGAGTCAGGACAGAGCTCTGGGTGGAGCTGTGCCTAAGG

>peak_H_18

AAACTCATTACACAGAAGCATGGTTAGTTGTTACTTTTCTCACTTCACATCATTCCTCTCAAAATAATTAAACCCTAATGAAGCTGCAAATAACTTCTGAGCAAAACAGATGATGTAACTGTGGAAGAGAGGGCAGCTGTACTTCAGGGACTGAAGCTCTGCAGTGGCACCTTCCTTCCCCCTGACACTGTGACAACATGCAACCTGAAGTCTGCAGTACCTGATGGCAGCCAAGAGCCCCACAGATCCAGACACTACAAATATGACCTGGCAAGTGTCACCTTCCCAGAGCTGCTCCAGAGCCTGTCTGCACCACAGAGGTCCAGGTGAGTACATGGAGGTAAGATCTTAGATGTCCTCAGCTGTGCCATGGAGAGATGCAATAAAGCTGAAGTCTTTCTCTGATTAAACAGGGAGTAGTTGAGAGGCTCTGTGAGCCTTGGGTCCTCCACTTTTCTTCTGACCATATCAAGCCTGATCCCAGGATCCAGAAAGATCAATGAAGACAAGTTCTTGGAGATACACAAATGGGACAGACATGCTGAGGC

>peak_H_19

gGAAATGCGGGAAGCTCAGTACACAGAAACATGGTTAGTTGTTACTTTTCTCCCTTCACATCATTCCTCTCAAAATAATTAAACCCTAATGAAGCTGCAAATAACTTCTGAGCAAAACAGATGATGTAACTGTGGGAGAGAGGGCAGCTGTACTTCAGGGACTGAGACTCTGCAGTGGCTCTGAGGCTCTTCCCACTGACATTGTGACAATAAGAAACTTGAAGTCTTCAGTACCTGATGGCAGCCAAGACCTCCACAGATCCAGGCAGTACAAATATGACCTGGCAAGTGTCACCTTCCCAGAGCTGCTCCAGAGCCTGTCTGCACCACAGAGGTCCAGGTGAGTACATGGAGGTAAGATCTTAGATGTCCTCAGCTGTGCCATGGAGAGATGCATTAAAGCTGAAGTCTCTCTCTGATTAAACAGGGAATAGTTGAGAGGCTCTGTGAGACTTGGGTCCTCCACTTTTCTTCTGACCATATCAAGCCTGATCCCTGGATCCAGAAAAATCAATGAAGACAAGTTCTTGGAGATACACAAATGGGACAGACATGCTGAGGCAACTGTTGTCCAT

>peak_H_20

ggagatgcattaAGCTCAGTACACAGAATTTGGTTAGTTGTTCATTTTCTCACCCCACATCCTTACTCTCAAAGTAATTAAACCCTAATGAAGCTTCAAATAACTTCTGAGCAAAACATATGTTGTAATTGTGGGAGAGAGGGAAGCTGTACTTCAGGGAGTGAGACCCTGCAGTGACTGTCCTTTCCCCTGATCCTGTGACATGAGACCTGAAGTCTGCAGTACCTGATAGCAGCCAAAAGCCCAAAAGACCCAGGCAGTACAAATATGACCTGGCAAGTGTCACCTTCCCAGAGCTGCTCCAGAGCCTGTCTGCACCACAGAAGTCCAGGTGAGTACATGGAGGTAAGATCTTAGATGCTCTCAGCTGTGCCATAGAGGGTTTCATTAAAGCTGAAGTCTCACTCTAAGACAGGAAGTAGTTGAATGACTCTGTGAGCCTTGGGTCTTTATTTTCCTTCTGACCCCAGCAAGGCTGATTCCTGGGTCCAGAGAGATCAAAGGAAACAAGTTTTAGGAGATATAGACGTGCTAAGGCATTGCTGTCCATCCTCAGGCTGGAGTCAGAACAGAGCTCTGGGTG

>peak_H_21

gGAAATATGGGAAACTCAGTACACAGAAACATGGTTAGTTGTTACTTTTCTCACTTCACATCATTCCTCTCAAAATAATTAAACCCTAATGAAGCTGCAAATAACTTCTGAGCAAAATAGATGATGTAACTGTGGGAGAGAGGGCAGCTGTACTTCAGGGACTGAGGCTCTGCAGTGGCTCTGAGGCTCTTCCCACTGACACTGTGACAATATGAAACTTGAAGTCTTCAGTACCTGATGGCAGCCAAGACCTCCACAGATCCAAAGAGTACACCTTCCCAGAGCTGCTCCAGAGTCTGTCTGCACCACAGAGGTCCAGGTGAGTACATGGAGGTAAGATCTTAGATGTTCTCACCTGTGCCATGGAGAGATGCATTAAAGCTGAAGTCTCTCTCTGATTAAAACAGGGAGTAGTTGAGAGGCTCTGTGAGACTTGGGTCCTCCACTTTTCTTCTGACCATATCAAGCCTGATCCCTGGATCCAGAAAAATCAATGAAGGAAAGTTCTTGGAGATACACAAATGGGACAGACTTGCTGAGGCAACTGTTGTCCATCTTT

>peak_H_22

AGTCAGGAAGCTCAGTACAGAGAACTTGGTTAGTTGTTCGTTTTCTCACTTCACAGCCTTCCTCTCAAAATAATTAAACCCCAATTAATCTGGAAATAACTTCTGAGCAAAACAGATGTCATAACTGAGGGAGAGAGGGAAGCTGTACGTCAGGGACTGAGGCTCTGCAGTGGCCTTCCTTCCCCCTGACACTGTGACAACATGAAACCTGATGTCTGAAATACCTGACGGCAGCTAAGAGCCCCACAGATCCAGACAGTACAAATATGACCTGGCAAGTGTCACCTTCCCAGAGCTGATTCAGAGCATGTCTGCAGCACAGAGGTCCAGGTGAGTACATGGAGGTAAGATCTTAGATTCTCTCAGCTGTGTCATGGATTCATTAAAGTTGGCTGTCTCTCTCTCTGACTAATCCAAGGTGTAGTTGAGAGGCTCTGTGAGCCTTGGGTCCTTGACTTTCCTTCTGACCCTGACAAGGCTGGTCCTGTGTCCAGAGAGATCAAAGGATATGAGTTGTAGGAGATAAAGAAATGCTGAGGCATTGCTGTCCAACCTCATGGTGGAGTCAGGACAGAGCTCTCAGTGGAGCT

>peak_H_23

GTAGAGTGTTGGATGTGACTCATAATGTTCTCCATAGAAAGAAGGACTTGAGAAAGAGAACAGATCTTTATCTGGATCAGAAGCAGGCCTCTATTCTGACTCATACATTTAGTTTAGCAATGTAAAGAGGGGTCCAGGAACATGGGGACTCTAGACATAGAAGATTGGTTGTTGTTCATTTTGTCTCTTTATGTGTTTCTATCATATAATGAAAATGTAATTAATCCAGAGGTAACTCTGGAGGCAAACAATTAAGGAGGGAGAGGAAAAGTCATCCTTTAGGGACTGCAGTTCTGCAGAGGGCTTCCTTCTCCCTGACATGGTGATAAGTTAACAACATCTTGAAGATACATTATGGTGACAAATTCTCTGAAAACGCCTAAGGTACAAATGTGACCTGGGAAGGCTCACTTGCCAGAAGACACAGAGCCTGTCTGCCCATGATCACTGTGGTGAGTAGATGTTTCAGTTTCTTTTACTCACATGCACATTTTGCTTAACGACACACATCTTCCTGTCTTTGACTAAGCTTAAGTGTAGAGGAGAGATGCTGAGTCTGGTCACCTTCACTCTTCATCCTATGCTTAAAACACAGAATAACTAGGGCCAGGGCCAGGAGAACTGATCCCACAAA

>peak_H_24

TCAGGAGATGCAGGAAGCTCAGAACAAAGAAACTTGTTTAATTGTTCATTTTCTCACCTCATATCCCTTCTCTCAAAATAATTAACATTAATTAAGCTGGAAATAAGTTCTGAGCAAAACATGCTGTAACTGTGGAAGAGAGGGAAGCTGTACTTCAGGGATTGAGACCCTGCAGTGGCCTTCCCTCTCCCTGACATTGTGACAACATGAAGCCTAAAGTCTGTAGTATCTGATGGAAGCCAAGGCTTCCACAGATCCAGACAGTACAAATATAATCTGGCCAGTGTCACCTTCTCAGCGCTGCCTCTGAGCCTGACTGGAACACAGAGGTCCAGGTGAGCACGTGGAGGTAAGAGCTTAAATCCCCCTCAGCTGTGCCATGGAGAGATGCATTAAAGCTGAAGTCTTTCTCTAAGACGGCGAGTAGTCGAGAGTCTGAAGGACTCAAGAGTCCTCCAGTTTCCTTCTGATCCTGACAAGGCTGATCCATGAGTCCAGAAAGATCAAAGGAGATGAATTCCTGGAGATATAGAAATGGGACAGAAATGCTGAGGGATTAATATTCATTCTCAGACTGTAGTCGTGGCAGACGTCTATGTGGATCTTTGCCTTAGTTATATTTTTAAAAGAAATCAAGATGACAGAGAAGATTTTTAGATGTCTATCAAA

>peak_H_25

gGAGATGCAGGAAGTTTACTATACAGAAACTTGGTTAGTTGTTCATTTTCTCACCTCACATCTTTCATCTCAAAAATAATTAAACATTAATTAAGCTTGAAATAACTTCTGAGCAAAACAGATGTCGTAACTGTGGAAGAGAGGGAAGCTGTACTTCAGGGACTGAAGCCCTGCAGTGGCCTTCCTTCCCCCTGACACTGTGACAACATTAAACCTGAAGTCTGCAGTACCTGATGGTAGCCAAGAGCCCCACAGATCCAGACACTACAAATATGACCTGGCAAGTGTCACCTTCCCAGAGCTGCCCCAGAGCCTGTCTGCAGCACAGAGATCCAGGTGAGTACATGGAGGTAAGATCTTAGATGCCCTCAGCTGTGCCATGGAGAGAGTCATTAAAGCTGAAATCTCTCTCTGATTAAGCCAGAGAGGGGTTGAGAGGCTCTGTGAGTCTTGTGTCCTTCACTTTCCTTCTGACCCTGACAAGGTTGGTCTCTTGGTCCAGAAAATTCAAAGGAGATGAGTTTTCGGAGATACAGAAATAGGACAGAAGTGCTGAGGCGTTGCTGTCCATCCTCAGGCTGGAGTCAGCACAGAGCTTTGGGTGGGGCATT

>peak_H_26

tgGAGACTCAGGAAGCTCAGTACACAGAACTTGGTTAGTTGCTCATTTTCTCACCTCACATCCTTCCTCTCAAAATAGTTAAACCCTAATTAAGCTGGAATAATTTCTGAGCAAAACAGATGCTGTAACTGAGGGAGAGAGGGAAGCTGTACTTCAGGGACTGAGGCTCTGCAGTGGCCTCCCTTCCCCCTGACACTGTGACAACATGAAACCTGAAGTCTGCAGTACCTGATGACAGCCAAGAGCCCCACAGATCCAGACAGTACAAATATGACCTGGCAAGTGTCACCTTCCCAGAGCTGCTCCAGAGGCTGTTTGTGCCACAGAGGTCCAGGTGAGTACATGGAGGTAAGTAAGACCTTAGATGCCCTCAGCTGTGCCATGGAGAGATGCATTAAAGTGGAAGTCTCTCTCTGGTTAAGATGGGGAGTAGTTGAGAGGCTCTGTGAGCCTTGGGTCCTCCATTTTCCTTCTGACCCTGACAAAACTGATCCCTGGGACCAGAAAGATTAAAGGAAACAAGTTACGGGAGGTACAGAAATGAAACAGAAATGCTGAGGCATCAATATCCATCCTCAGTCTGGAGTCAGGACAGAGCTCCAGGTGGATCTTT

>peak_H_29

TCAGGAAATTGGGGAAACTCAGTACACAGAAACTTGGTTAGTTCTTCATTTTCTCACCTCACATTCTTCTTCTCAAAACAATTAAACTCTAATTAAGCTGGAAATAACTTCTGAGCAAATCAGATGTCGTAACTGTGGGAGAGAGGGCAGCTGTACTTCAGGGACTGAGGCCCTGTGGCCTCCCTTCCCCCTGACACTGTGACAACATGAAACCTGAAGTCTGCAGTACCTGATGGCAGCCAAGAGTCCCACAGATCCAAACAGTACAAATATGACCTGGCAAGTGACACCTTCCCAGAACTACTCTAGAGCCTGTCTGCAGCACAAAGGTCCAGGTGAGTACATGGAGGTAAGATCTTAGATGTCCTCAGCTGTGCCATGGAGAGATGCATTAAAGCTGAAGTCTTTCTCTGATTAAGACAGGGAGTAGTTGAGAGGCTCTGTGAGACTTGGGTCCTCCACATTCCTTCTGACCCTTACAAGGCTCATCAATGGGTTTAGAAAGATCACAAGATACAGGAATGGGACTGAAATGCTGAGGCATGGCCATACATCCTCAGGCAGAGTCAGGACAGAACTCTAGGTAGGTCTTTGAGGTTACCTCCTTACCTTTTTAAAAGGATTCAGTACGACAGAGAAGATTTTCAGTTG

>peak_H_31

TCAGGAGACTCAGGAAGCTCAGTACACAGAACTTGGTTAGTGGCTCATTTTCTCACCTCACATCCTTTCTCTCAAAACAATTAAACCCTAATCAAACTGGAAATAAGTTCTGAGCAAAACAGATGCCGTAACTGTGGGAGAGAGGGAAGCTGTACTTCAGGGACTGAGGCTCTGCAGTGGCCTCCCTTCCCCCTGACACTGTGACAACATGAAACCTGAAGTCTGCAGTACCTGATGGCAACCAAGAGCCCCACAGATCCAGACACTACAAATATGACCTGACAAGTGTCACCTTCCCAGAGCTGCCCCAGAGCCTGTCTGCAGCACAGAGGTCCAGGTGAGAACATGGAGGTAAGACCTTAGATCCCTTCAGCTCTGTCATGTAGAGATACCTTAAAATTGAACTCTATCTCTGATTAAACCAGAGAGAGATTGAGAGGCTCTGTGAGCCTTGGTCCTCCACATTCTTTCTGACCTTGACAAGGCTCATCCCTGGGTTCAGAAAGATCAAAAGAGACAAGTTCCTGGAGATACAGAAATGAGACTGAAATGCTGAGGCATGGCTCTCCTTTCTCAGGCTGGAGTCATGACAGAGCTCTGGGTGGGTCTTTGGCTGAGATTACCTCCTTACCTTTTTTAAAGGATTCAGTAAGATAAAGATTTTCATATGTCTATCAAGCAATGGAGACA

>peak_H_32

TCAGGAAATTGGGGAAACTCAGTACACAGAAACTTGGTTAGTTCTTCATTTTCTCACCTTACATCCTTCCTCCCAAAATAATTAAACTCTAATTAAGCTGGAAAAAACTTGTGAGCAAAACAGATGTAGTAATTGTTTTAGAGAGGGCAGCTTTACTTCAGGGACTTAGGGCCTGCAGTGGCCTTTCTTCCCCTTGACACTGTGACAACATGAAACCTAAATTATGCAGTACCTAATGACAGCCAAGAGCCCTACAGATCCAGACAGTACAAATATGACCGGGCAAGTGTCACCTTCCCAGAGCTGCTCCAGAGTCTGCCTGTACCACAGAGGTCCAGGTGAGTACATGGAGGTAAGACCTTAGATGCCCTCAGCTGTGTCATGAAGAGATGCATTAAAGATGAAGTCTCTCTCTGATAAAGACAGGGAGTAGTTGAGAGGCTCTGTGAGACTTGGGTCCTCCATATTCCTTGTGACCCTTACAAGGCTCATCCATGAGTTTAGAAAGATCAAAAGAGACAAGATCCTAGAGATACAGGAATGGGACTGAAATGCTGAGGCATGGCCATACATCCTCAGGCAGAGTCAGGACAGAACTCTAGGTAGGTCTTTGAGGTTACCTCCTTACCTTTTTAAAAGGATTCAGTACGACAGAGAAGATTTTCAGTTGTCTATCAAACCCTGGCGAT

>peak_H_33

ggagatatGGGAAGATCAGTTGGTTACTTGTTCATTTTCCCACCTCACATCCTTCCTCTCAAAATAATTAAACACTAATTAAGGTGGAAATAACTTCTGAGCAAAACAGATTCTGTAACTGTTGGAGAGAGGGAAGCTATACCTCAGGGACTCGGGCCCTGAAGTGACCTTCCTTTCCCCTGACATTGTGACAATATGAAACCTAAACTTTGCAGTACCTAATGACAGCCAAGAGCCCCACAGATCCAGACAGAACAAATATGACCTGGCAAGTGTCACCTTCCCAGAGCTGCTCCAGAGCCTGTCTGCACCACAGAAGTCCAGGTGAGTACGTGAAGGTAAGATCTTAGATGCCCTCAGCTGTGCCATGAAAGCTGAAGTCTCTCTCTGACTAAGACAGAGAGGAATTGAAAGGCTCCATGAGCCTTGGGTCCTCCACATTCCTTTTGACCCCACAAG

>peak_H_34

GGAGATGTGGGAAGCTCAGTACACAGAAACTTGGTAAGTAGTTAATTTTCTCACATCCTTCCTCTCAAAATAGTTAAACCCTAATTAAGCTGGAGATAAGTTCTGAGCAAAACAGATGCTGTAACTGGGAGAGAGGGCAGCTGTACTTCAGGGACTGAGGCCCTGCAGTGACCTTTCTCCCCCCCTGACACTGTGACAACATGAACCCTAAAGTCTGCAGTATCTGATGACAACCAAGAGCCCCACAGATCCAGACAGTACAAATATGACCTGGCAAGTGTCACCTTCCCAGAGCTGCTCCAGAGCCTGTCTGCAGCACAGAGGTCCAGGTGAGAACATGGAGGTAAGATCTTAGATGCCCTCAGCTGTGCCATGGAGACTAAAGCTGAAGTCTCTCTCTGACTAAGACAGAGAGGAGAGGCTCTATGAGCCTTGGGTCCTCCACTTTCCTTCTGACCCTGGCAAGGATGGTCTCTTGGTCCAGAAAGATCAAAGGAGATGAGTTCCTGGAGATACAGAAATGGGACAGAAATGCTGAGGCTTTGCTCTCCATCCTCAGGCTGGAGTCAGCACAGAGCTTTGGGTGAGTCTTTGCCTAAGGTTATCTTTTAAAAAGGAATCAATAAGATACAGAACATTTTCAAATATCTATAAAAAAT

>peak_I_3

TCCTTACTCACAGACACTGTAATTGGAATCATAAAGTTTCTGGGTTCAGCAAATGACAGAGCACCAGGGAAAAGGCAGTGCTGGCCTTGAGGGTGGAGACAGAAAAGTCTCTTCCCCTGACAAAGCCAAACTCCTGGTGGTGACATCCTGTCACCTTGCTTGAACACAAGGACTTGAGGTTGATAAACAAGGCCTGGCTGTGCTGTGAGGACACCGACCCTGAGCCCTGCACCGGCTGCACAGGTGAGTGCTGCCATGTCATGGGAGATGGGGCTGCATTTGACCTTGATAGATATCTGTGGAGGGCTGATGTGTGGGAGAGAAGGATGTGGGCTCCTTGTCACTCTGAGAGGGGACAGTGGCAGGATTCAGGGAGAGGATGGTCTATCATTTCCTAAGTGCGATCCAGAATTGTCTCAGTGTCCACAAGGACAGTCCCAGCTCCTTACACAGGACAGGCAGGGGTAGGAGACAGAGGCACCTGCAGTGCAGCCTGGAGTAGGAAGCTCTTGCTCCTACTGCTTCTCTTGGTTGTGTAACTTCTGGTGACTTCAGTCTTCTGTGCATGGAACATTCTTTCTTTCTTTATGGTGATTTCATTTATTGTAATTTCTCCTTTTCTAGATCAAATGTAAACCATCGAATATGAGAATTGCGGATAAAACAGTCATGAGGCTAAATACTGGTAGTAACATCTGATTTTAGAAAATGCATTTTTAGTGCATAGTGGGAACCTCACAAGGTACTATTCCTATCAGTTCATTGACTCCCTAAATTATACCTGTATTACATAAAAGCATactttacatatgaaaagtactttttaaagaagccaaataagtatgctcaaggtgatgaacatatgaattggaaagccagaatttaaatctactttgggtggtcacaaaggatgttcttGTGAATACTGAGCTTTCTA

>peak_I_5

CAGGTACTGCAGTTAAAAGCAGAAAGTCTCTATCTCAGCAAATGACAGGACACCAGGGAGAACACAGACCTGACATATAGGATAGAGATGTGCAGAAGGAGCCTGTTCCCTGGAGAAGCCAAACTCCTGCTGTTGACATCCTGGCAGCAGTAAAATTTCTTGTGAGCTCAGGGCTCAAGGATTATAAATAGGGCCTGGATATGCTCTAAGGACAACCCTGAGCCCTCCATAGACTCTGCAGGTGAGTGTCCCATTATGTCACAGTGGATTACAGCAGCCTTTCACGTTGACAGAGACACTTTGAGGTAGGGATAGTTAGGGGAGGTGGTTGAAGAGAAAGTTTTGGTTTCTCTGTAACTGTGAAAGAAGAAGGTGATAAGATCCAGAAAGAAAGGAGTCTCTAATTTCCCACCTGCCATCCAGAATTATTTCCATGACCATAAGGGAGCTAAGTGCAGCTGTGTGTGATGCACTGAACCAGAGATGCTCATTACAGCTCAGGCAGGTACAGGAGACATGGGCTGAAGTTTCAGCTTAAGTGTGAGGAAGCTTTTTTTCTTTGCAGTTTCTATAGTTTGTATGTTTGTCCTCACAGGAGGGAAAGGTTCTCCTCAACAGTGTCTTATATCAGGTGCCCTTTAGCTTTCTCACAGTGGGAGACTTTGCCCTTCTTGTCATGGTTTTATTGTTCCTAAAGTTTCTCCTGATTCAGAACATAACATAACTATGCAGGAAACTGACATGACATAAAATCCtagagcaacggctattagagcatgtttaacacggggcagacactgttctaagcactatgtgcctctgctcctctgaaacctcacaatacacctgaatggtacaaagcaacttccctacattagtgagtcttaaagggaccaagtaaagtattctaagtccagcacataacaattgggaaagacagcctttaacagatagaataactggttgcaaaac

>peak_I_6

GCCAGAGCCCCTTGGGCACACTTCTCCTTACTCACAGACACTGTAATAGGGATCATAAATCTTCTGGGTTCAGCAAATGACAGAGCACCAGGGAGAAGGCAGAGCTGCCCTCAGGGATGGAGACAGAAGAGCCTTTCCCTGCTTTGGGGAACATCCAGACACCTTGCTTGAGCACAGAGATTTGAGCTTCATAAGCAGGGCCTGACTGTGCTGTGAGCACACCCACCCTGAGCCCTGCAGACTGCACAGGTGAGTGCTGCCATGTCATGGGGAATGGGGCTGCAGTCAACACTGAAAGAAATCATGGAGGGCTCATGTATGGGAGAGAAGGATGTGGGCTCCCTGTCACTCTGAGAGGGAACAGCGACAGGGTTCTGAGAGAAGATCATTTCCTAAATGCAATCCAGAACTGTTTCTGTGTCACTGAGCACGAGCTGCTCCTTACACTGGACAGGCAGGAGTAGGAGACAGAGGCACCTGCAGTGCAGCCTGGAGTAGGAAGCTCTTACTCCCATAGCTTCTTCACTATCTGTGTGATTTTCCCTCTAAAGACTGAGAACTTTAATCTGATTTGATTAACTTCTGGTAACTTCAATCCTTTGTGGCTAGTGCACTCTTTCTTTCTTTTGTCATGATTTCATTTATTGTCAATTTCTCCTTTCCTAGTTCCAATGTAAACCATGAAATATGAGAATAATATAAAACAGATATGAGACTATATACTAACACTAACAACCAATTTTAGAAAATGGATCTTTAAAGCATGCTGGGAACTTTCCTAAGTATGTCAGCTCATTGACTCCCTAAAATATACCTATATTGCATAAAACATAATTTACATATAAAAATACTTTTTATAGGAATCAAATAATATACTGAAACTTATGAATACATGTAGTAGGGAAAACAGAATTTACATCTACATCTGGGTGTTCATAAAACATCTTGTGAATCCTGAACTTTTTATTACTAATGTTTGGTATCACTCAAGCTCCTGGA

>peak_I_7

CAGGAACTGTAATCACACTCATAATGATTTTAGATCACGAATGTCAGAGAACTAGGGAGAAGGCTGACCTTGGGGACGGAGATGTGCAAAAGGAGCCCTTTTCCCCTGACATCAGTGTGAGCTGCTGTGAACATGGGGAATAAGGAATGAAAACCAGGGTCTGGCTGTGCTGTGAGGATACCCACCCCATTCCTGCACAGACCAAGCAGGTGAGTGCTGTCCTATAGTTGTGGACTGAGGCTGCCTTTGACTTTGGAAGGAGAGCTGTGAGGCTGGGAGGCTCTAGGTAGGGGAAAGGAGTGTCCCAGTCATCTGTCACTGTGCAAAAAGAAGGAGGCAGGACCCGGGGAGAAAACAGTTTATCACTTCCCAGCTGTCATCCAGGACCTGTTTTAGGGAGTGTTTGCAGCAGTTTGGGGTGCACTGTACCAGAGCTGCTCATCAAGTGGGCCAGGCAGGGACAGGATAGGGGCCTGGTGGCTTCTCAGTCTGGTTGTGAGATAAGACTCATTTTCTCCACAGCCTTACACACCTTACCCT

>peak_I_11

TCCAGGAAACTCAGGCACTGTAATTACAACCAGAAACTTTCTGGCTCAGCAAATGACAGGGCACCAGAGAGATCACAGACCTGACATCAAGGATGGAGATTGCAGAAGGAGCCCTTTCCCCACGGAGAAGCCAAACTTCTAACAGTGACATCCTATTAGCAATGACAGTTGCTGTGAGCCCAGGGCTCAAGGACAATAAACAGGACCAAGCTATGTTGTGGGGGCACCAACCCTGAGCCCTGCACAGACTCTGCAGGTGAGTGCTTCATCATGTCACAGTGAACTGAAGCTGTCTTTCACATCAACAGAGATGCTGTGAGGTAGGGATGGTTAGGGGAGGTGATTGAAGAGAAGGTCTGGGTTCCTCTCTCACTGTGAAAGAGGAAGGTGACAAGATCCAGGAAGACAGCAGTCTCTCATTTCCCACCTGCCATCCAGAAATTTTTTCATGTCCATGAGGGAGCTGAGTGCAGCTGTGTTGGGTGCACTGAGCCAGAGATGCTCATTACAGCTCAGGCAGGTACAGGAGACATGGTGTGAAATCTCAGCTTGAGTGTAGGGAAGTTTTACTTCTTTGCAGTTTCTCCAGTTTGTGTGTTTGTCCTCACAGGAGGGAGAGGTTCTCCACAACAGTGTCTTATATCAGGTGCCCTTTAGCTTTCTCACCGTGGGGGACTTTGCCCTTCTTTGTCATGGTTTTATTGTTCCTAAAGTTTTTCCTGATTCAGAACATAACACAACTATGCAGGAAACTGACATGACATAAAATCCTAGAGCAATGGCTATTAGACCATGTTTAACACTGGGcagacactgtcctaagcaatatgtgcctctcctccactgaattctcacaatacacctgaatggtacagaaccatcttcccgacattagtgagtcttaaagggtccaagtaaaacattcaaagctaggcatgtagcacctaggaaatgcagaactggcagacagaattgc

>peak_I_14

GAGGAAAAAGTCATATTCTCGAGGAAACTCAAGGTACTGTTTGTCCTCACAGCCCTCCTTGACAACACAGGCACTGTAATTAGAACCAGAAAGTTTCTTGCTCAGCAAATGACAGGGCACCAGGGAGAGCAGAGAGCTGACATCTAGAATGGAGATTGCAGAAGGAGCCATTTCCCCAGGAGAAGCCAAACTCCTGCTAGTGACATCCTATTAGCAGTGACAGTTGGTAGGAGCACAGAGCTTGAGGATGATAATAAACAGGGTCTGACTGTTCTCTGAGGGCACTTCTGAGTTGCACAGGTTCCACAGGTGAGTGCCCCATCATGTCACAGTGGACTGAAGTTGCCTTTTGCCTTCATAGAGGTGCTGTGAGGTACGGATGGTTAGGGAAAGTGGTTGAGAGGAAGGTGACAAGATCCAGGAAGAAAGCAGTCTCTCATTTCCCACCTGCCATCCAGAATTATTTCCATGTCCATGAGGGAGCTGAGTGCAGCTGTGTGTGATGCACTGAGCCAGAGATGCTCATTACAGCTCATGCAGGTACAGGAGACATGGGCTGAAGTTTCAGCTTGAGTGTAGGGAAGCTTTTCTTTGCAGTTTCTCCAGTTTGTGTGTTTGTCCTCACAGGAGGGAGAGGTTCTCTCAACAGTGTCTTATATCAGGTGCCCTTTAGCTTTCTCACAGTGAGGGACTTTGCCCCTCTTTGTTGTGGTTTTATTGTCTCTGAAGTTTCTCTCTCCTGATTCAGAAAATAGCACAATTGTGCAGGAGATGGACATGACACAAAATCCTAGAGTAATGGCtattagagcatgcttaacactgggcagacactgtcctaagcactatgtgcctctgctccattgaatcttcacaacacctaaatgatacaaagcactttctctacattaatgagtcataaagaggccaagtctgatgttcaaagcccaccatacaacaagtagaaaagacagcattTACGAGAGAGGTTACAAAGCTTAAACTTGTCAA

>peak_I_15

GCACTGTTCACAGCCACTGCAATTAAGATCGAAAGTTTCTGGGCTCAGCACCAGGGAGAAGGCAGAGCTGGCCTCAGGGATGGAGACAGAAGAGTCTTTTCCCCTGATGAAGCCAAACTCCTGGTGGTGGCATCTTGACACCTTGCTTGAACACAAGGACTTGAGGTTGATAAACAGAGCCCGGCTGTGCTATGAGGACACCCATCCTGAGTCCTGCACTGATTGCACAGGTGAGTGCTGCCATGTCATGGGTCATAGGGCTGTGTTTGTCCTTTATAGAAATCTGTGGAGGGCTGATGTGTGGGAGAGAAGGATGTTCCTTCCTTTTCACTCTGAGAGGGGACAGTGGCAGGATTCAGGGAGAAGATGGTCTATCATTTCCTAAGTACAATCCAGAATTGTCTCAGTGCCCACAGGGACAGTATGTGTGGTCACTGGGCATGAGCTGTTCCTTACACAGGACAGGCAGGGGTAGGAGACATAGACACCTGCAGTGCAGCCTGGGGTAGGAAGCTCTTGCGGCCACAGTTTCTGCACAATCTGTGTTATTTGCTTCCAATGACTTAGTACTTTGATCTGAGCAAATTTCTGTTGACTTCAGTCCTTTGTGGATAGAATAGTCTTTCTTTCTCTCTTTATCATGATTTTATTTATTGTGAATGTCTTCTTCTATAAACCTAATGTAAACCATGGAGTATGAGAATTGTGGATAAAACAGCCATGAGGTTACATACTAACTCTTACAACTTATTTTAGAAAATTGATTTTTAAAACATTCTGAGCACTTCATTAGGTATTATGTATGCTAGTTCATTGACTCTCTAAAATATACATGTATTGTATAAAACATAATTTACATACAAAAATAGTTTTTAAAGAAGTCAAATAAATAATATGCTCAAAGTGGTGAATATATGAATTGGGAAATCAAAAACTTTAATTCACAGTGGGTGGTCACAAAGCA

>peak_I_27

AATGCTTCTGTTTCTGAGAAGTTTCTCCCAATTCAGTTCCTAGGAGAGAAAAGTCCCAGGGACCAAGAAACAGAGGTACTACAGTTAGAAGCAGAAACTTTCTGTCTCAGCAAATGGCAGGGCACCAGGGAGAACACAGACCTGACATCTAGGATAGGGATATGCAGAAGGAGCCTGTTCCCTGGAGAAGCCAAACTCCTGATGATGACGACATCCTAGCAGCAGTGAAAGTTCTTGTGAGCCCAGGGCTCAAGGATTATAAATGAGGTCTAGTTGTGCTGTGAGGGACACCCACCCTGAGCCCTGCACAGACTCTGCAGGTGAGAGTTCCATCATGTCAGAGTGGACTGAAGTTGCCTTTCACCTTGACAGAGACACTGTGAGGTAGGGATAGTTAGGGGAGGTGACTGGAGTTTCTGGGTTCCTTTGTCACTGTTAAAGAGGAAGGTGACAAGATCTAGGGAGAATCTTTCATTTCCCACCTGCCAATCAGAATTATTTCCATGACCATGAGGGAGCTGAGTGCAGCTGTGTGTGGTGCACTGAGCCAGAGATGCTCACTACAGCTCAGGCAGGTACAGGAGACATGGGCTGAAATTTCAGCTTCATATGAGGAAGCTTTTCCTCTCTGCAGTTTCTCCAGTTTGTGTGTTTGTCCTCACAGGAGTAAGAGGTTCTACTCCACAGTGTCTTAAATCAGGTATCCTTTAACTTTCTCATAATGGGGGACTTTACCCTTTGCCTTTCTTTGTCGTTGTTTCATTGTTCCTGAAGTTTCTCTCTCCTGACTCAGAATATAACACAATTGAGCAGAAAACTGACATGACACAAAATCCTAGAGCAATGGCTATTAGAGCATGTTTAACACTGGGcagacactgtcctaagcactatgtgcctctgctccattgaatcctcacaatacgcttgaatggtaCAAAGTACCTCTGTACATTAGTGAGTCTTAAAGGAGCCAAGAAAAATGTTCGAAGACCAGCATATAACAATTAAGAAAAACAACATGTAACAGACATGTTACAAAGCTTGA

>peak_I_28

AACCCTTGGGCACTGTTCATCAACCTTCTCCTTACTGAAAATACTTTAATTAGGATCATAGTGTTTCTAGGTACAGCAAATGACAGAGCACCAGGGAAAAGGCAGAGCTGCCCTCAGAGATAAAGACAGAAGAGCCTTTTCCCCTGATTAAGCCAAATTCACAGTGGGTACATCCTGGCACCTTGCTTGAGCACAAGGACTTGAGGTTAATAAACAGGGGTTGGCAGTACTTTGAGGACACCCACACTGAGCCCTGCATATACTGTACAGGTGAGTGCTGCCATGTCATGGTGGATGGGGCTCCTTGAGACTGAAAGAAATGTGCGGAGGTCTGATGTATCAGGGAGAAGGATGTGGACTCCCTGTCTACTCAGTGACAGGATTCATGGAGAAGATGGTAAATCATTCCCTACATGCAATTTAGAATTGTCTCAGGGTTCACAAGGACATTCTATGTTTTCACTGGCAGTGATCTGATCCTTACATAGGACAGGGAATGGTAGGAGGCAGAGGCACCTACAGTGCAGCCTGGAATAGGAAGATCTTGCTCCCTTAGCTTCTGCACAATCCGTGTGATTTGCCTTCAAAGACTGAGGACTTTGGTTTGGACTTTGTAACTTATGGTGACTTCAGTCCTTTGTAGATAGAATAGACTTTCTTTCTTGACCATGATTTCATTTATTGTGAATTTCTTCTTTTCTACACCTAATGTAAACCATGGAGCATGATGATTGTGGATAAAACAGCTATGAGATTACTTACTAACTCGAACAACCTATTTTAGAAAATTGACACTTCTCTAATTATTATGTGTGCTAGATCATTGGCTCTTAAAAATGTACATATATTGTACAAAACATAATTTATATAAAAACAGCTTTTAGTGAAGCCAAAATAATACGCTCAAAG

>peak_I_30

ACCACAGGGACTGTAATTAGGACCAGAAAGTTTCTGGCTCAGCAGAAGAAAGGGCACCAGAGAGAGCAGAGAGCTGACATCTAGAATGAAGATTGCAAAAATAGCATTTTCCCCAGGAGAACCCAAACTCCTGCTAGTGACATCCTATTAGCAGTGAAAGTTGTGAGCACAGAGCTCAAGGATAATAATAAACAGGGCCTGTCTGTGCTCTGAGGACACCCCTGAGCCCTGCACAGACTCTGCAGGTGAGTGTCCCGTCATGTCACAGTGGACTGAAGTTGCCTTTTTCGCCTTCACAGAGATGCTGTGAGGTAGGGATGGTTAGGGAAGGTGGTTGAAAAGAGGGTCTGGGTTCCTCTGTCACTGTGAAAGAGAAAGATAACAAGATCCAGGGAGAAAGCAGTTTCTCATTTCCCACCTGCCATCCAGAATTATTTCCGTGTTCATGAGGGAGCTGCATGCAGCTGCCTATGGTGCACTGAGCCAGAGATGCTGATTATAGCTCAGGCAGGTACAGGAGACATGGTGTGAAATTTCAGCTTGAGTGTAGGGAAGCTTTTCTTCTTTGCAGTTTCTCCAGTTTGTGTTTGTCCTCACAGGAGGAAGAGGTTCTACTCAACAGTGTCTTATATCAGGTGTCCTTTATCTTTCTCACAGTGGGGGACTTTGCCCTTCTTTGTTGTGGTTTTACTGTTCCTGAAGTTTCCATCACCAGAGTCAAATGTAAAACTGTGCAACTATAGAGTATTGTGCAGGAAACAGACATGACACAAAATCCTAGAACAACGGCtattggagcatgattaatgctgggcagacactgttctaagcactatgtgcctctgctccactgaaacctcacaatacacctgaatggtacaaatcacattccctacattagcaagtctcaatggggccaagtcagatgttcaaagtccagcatataacaattaggaaagacagcatttaagagactggttacaaagcttgatcttgtcgattttacaatt

>peak_JK_113

TATGCTAACAGCTGTGATAATTCCCACCACTGTTTTCAAACTTTTAATGACTTCTTCACTCCTCAAGTTTACTTTCTGATATGTATCATCTTGGGGAAATGCAAATATTCAGAAGTAGATTTTGCACTCTTAGAATGCTAAAGTTATCTGGGTGAGCAATGTTTTCCCCTGAGCATATCTAAGTAATGCGCAGTAACAAGCTCATTGATGCCTTGGGTGCCAAGCTGGTCCATGTCCACTGGGAGGAAGAGTCCTGTCATCCTGTCCTTGATTTAGCCATGCCACCTGCTAGGCAGCCTTTCCAAGACACATCCTATCCGTGGGATTCACACAGCCCTGAAGGGCTCCCAGTGCTCAGCGCTCAGAGGCAGAGGAAGGTATGATGAGCTTCACACTGATCCTCTTCCCCGGTCTATCCTGTCTTAGACTTGCACTGTTTTCCTTCAGATTTCTTTTCTCCTATTTTTCTTTCCAGATAACTAAATTGTATCTTTCTCCTTTTTTTGTACCAAATCTTGTCTTTAGCTTATAATATTCTCACTATTTTCCTACTtttatttgcttatatatttacttatttgtttattgatttatttGCATCTGT

>peak_JK_114

AAGATAATGGCTGTGATAATTTCCACATCTACTTGAAAACTTTTAATGATTTTCTAAATTGTAAAGTTTTCTTTCTGATGTAGTTCAACTTAGGAAAAAGCACATCTCAGACAGAGAATGAGCTCTTATTTACTTGCTTAATCAATTGCCATGAGAATCACACTGTATCTGGGAGAGTAATGTTTTCCTTGGAGTGCATTAAAATGATGCCCAGTAAACAAGGTCATTGACACCATGGGAGGGAGAGTCCTCCTATCCCATCCCTTATTTAGTCCTGTCTCCTATGAGGCAGCCTTTCCAAGACACTTTGTCCCTGTGATGCTTCATGTCTGTGGATACACACTGCCCTGAACCAGGGTTTGAAGCACTCCACACTCAGAGCAAGAGGAGGTATGGCAACCTTCCCCTGTTTCTCTCTTCTCCCTTAGTCTACCATTCTATTTACAATACTTTATAGTACCTTACACTTTCGGGGGTTTGTTTTTCAGATGAGTTTTTCTCCTATTTTTCTGTCAAGTTAGGATATCCTTTTCTTATTTTTTGCACAATACTCCTCTTTGTCTTCATAAATGTATTTTCTTCTTTCATTTtttattcatgtattttatgtg

>peak_JK_115

CAGAGAGTGTGCTCTCTGGAGTCCTGTTCACTGCTCAGCCTGCTTGAATGGCTGTGATGACATCCACCACTACAGAAGAACTAACTTTTAATGACTTTTCAGTTCCCAGAGTGGACTCTCTGGTGTAGCTCAACTTGGGGAAAGGTACGTCCTCAAAGGCACCTGGCTTAGTCTGATTAATAAGTTCACCAGGAGACTCATTTTAAAGGTGGAAGACTGATATATTATTCCTCTGAGTGCCCCAAAAGGATGCACTAAAACAAGGCCATCAATACCTCAGGTAACATTGAGGATTGGGGCAAGTCAATTGGAAGGATACATTCTGTGGGCAGTTTAGCTATGGGTGTGTTTAGGAAGCCTTCCCAGCACACCTGTCTGTGGGATGGAGTCACATCCAATGAGGATGCCACAACACCCTAGCCTGAGACTGACAGAAAGGTATGAAGACCCTGTCAATGTCCATCTCCCAGCCTCCTTGTGCAGACCAGGATCTTATCAGATATTTTGTCTCCTGTCTCCTACCAAATATATTCCCTCATTTTTATTTTCTCTACATCTCTTTACGTTCACAAACATTTACCATTTTCTATTCTTTATTTTAGTATGTGTTCTAGCATGTATTCTAGATGTACTCGTGTAGAACCCCATCTTCTCATCTTTTCACAAGTATTTGCATTGGCCCTCTGCCATCTA

>peak_JK_116

ATGATAATGGCTGTGATAATTTTCACATCTATTTGAAAACTTTTAATGATTTTCTAAATAGTAAAGTTTTCTTTCTGATGTAGTTCAACTTAGGAAAAAGCACATCTCAGACAGAGACTGAGCTCTTATTTACTTGGCTTAATCAATTACCATGAGAATCACACTGTATCTGGGAGAGTAATGTTTTCCTTGGAGTGCATTAAAATGATGCCCAATAAACAAGGTCATTGACACCATGGGAGGGAGAGTCCTCCTATCCTGTCCCTTATTTAGTCATGGCTCCTGTGAAGCAGCCTTTCCAAGACACTTTGTGTCAATGATGCTTCATGTCTATGGAATTTACAATGCCCTGAATGAGGGTTTGTAGTACTCCACACTCAGAGCAAGAGGAGGTATGGCAACCTTCCCCTGTTTCTCTTTTCTCTCCTAGTCTACCATTCCATTTACAATACCTTATAGTACCTTACACTTTGGGGGTTTTGTTTTTCAGATGAGTTTTTCTCCTATTTTTCTGTCAAGCTAGGATATCCCTTTCTTATTTTTTGCACAATACTTCTCTTTGTCTTCATAAATGTATTTTCTTCTTTAAATTTATTCATGTATTTTATGTGTATGTTGGTTTTCCTACATGTATGtctgtgaagactggccagtg

>peak_JK_117

ataaaagtagacataagtagaaagagaaattggacaaagatttgaagacttgacaatacaattttgaatgaacagtggatcattgcagaaatcaacagggaaatgtagaaaTTTCTACATTTCATTTCAGATGATAATGGCTGTGATAATTTCCACATCTACTTGAAAACTTTTAATGATTTTCTAAATTGTAAAGTTTCCTTTCTGATGTAGTTCAACTTAGGAAAAAGCACATCCCAGTCAGAGACTGAGCTCTTATTTACTTGGCTTAATCAATTGCCATGAGAATCACACTGTATCTGGGAGAGTAATGTTTTCCTTGGAGTGCATTAAAATGATGCCCAATAAACAAGGTCATTGACACCATGGTAGGGAGAGTCCTCCTATCCCATCCCTTATTTAGTCCTGTCTCCTGTGAGGCAGCCTTTCCAAGACACTTTGTCCCTGTGATGCTTCATGTCTGTGGATCCACACTGCGCTGAACGAGGGTTTGAAGCACTCCACACTCAGAGCAAGAGGAGGTATGGCAACCTTCCCACTGTTTCTCTCTTCTCCCTTAGTCTACCATTCTATTTACAATACCTTACAGTACCTTACACTTTGGGGgttttgttttgttttgttttCAGGTATGTTTTTCTTCTATTTTTCTGTCAAGCTAGGATACCCCTTTCTTATTTTTCTGTACAATACTTCTCTTGATCTTCATAAATGtattttattttttaaatttttattCATGCTATATATATATATATGTACACACATATATGTtggttttcctgcatgtatatctgtgaagcacatcagttcaggactccaggaagccaggaaaaggtgtcagatccttt
